# Postoperative analgesia for upper gastrointestinal surgery: a retrospective cohort analysis

**DOI:** 10.1186/s13741-023-00324-0

**Published:** 2023-07-18

**Authors:** Katrina P. Pirie, Andy Wang, Joanna Yu, Bao Teng, Matthew A. Doane, Paul S. Myles, Bernhard Riedel

**Affiliations:** 1 Department of Anaesthesiology and Perioperative Medicine, Alfred Hospital, Melbourne, Australia; 2 Central Clinical School, Monash University, Melbourne, Australia; 3 Sydney Medical School (Northern), Faculty of Medicine and Health, The University of Sydney, Sydney, Australia; 4 Department of Anaesthetics, Royal Prince Alfred Hospital, Sydney, Australia; 5 Chris O’Brien Lifehouse, Sydney, Australia; 6 Department of Anaesthesia and Perioperative Medicine, Royal North Shore Hospital, Sydney, Australia; 7 Kolling Research Institute, Sydney, Australia; 8 Northern Sydney Anaesthesia Research Institute, Sydney, Australia; 9 Department of Anaesthesia, Perioperative and Pain Medicine, Peter MacCallum Cancer Centre, Melbourne, Australia; 10 Department of Critical Care, University of Melbourne, Melbourne, Australia; 11 Department of Oncology, Sir Peter MacCallum, University of Melbourne, Melbourne, Australia

**Keywords:** Thoracic epidural analgesia, Intrathecal morphine, Upper gastro-intestinal surgery, Pain control, Opioids, Laparoscopic, Laparotomy

## Abstract

**Background:**

Thoracic epidural analgesia is commonly used for upper gastrointestinal surgery. Intrathecal morphine is an appealing opioid-sparing non-epidural analgesic option, especially for laparoscopic gastrointestinal surgery.

**Methods:**

Following ethics committee approval, we extracted data from the electronic medical records of patients at Royal North Shore Hospital (Sydney, Australia) that had upper gastrointestinal surgery between November 2015 and October 2020. Postoperative morphine consumption and pain scores were modelled with a Bayesian mixed effect model.

**Results:**

A total of 427 patients were identified who underwent open (*n* = 300), laparoscopic (*n* = 120) or laparoscopic converted to open (*n* = 7) upper gastrointestinal surgery. The majority of patients undergoing open surgery received a neuraxial technique (thoracic epidural [58%, *n* = 174]; intrathecal morphine [21%, *n* = 63]) compared to a minority in laparoscopic approaches (thoracic epidural [3%, *n* = 4]; intrathecal morphine [12%, *n* = 14]). Intrathecal morphine was superior over non-neuraxial analgesia in terms of lower median oral morphine equivalent consumption and higher probability of adequate pain control; however, this effect was not sustained beyond postoperative day 2. Thoracic epidural analgesia was superior to both intrathecal and non-neuraxial analgesia options for both primary outcomes, but at the expense of higher rates of postoperative hypotension (60%, *n* = 113) and substantial technique failure rates (32%).

**Conclusions:**

We found that thoracic epidural analgesia was superior to intrathecal morphine, and intrathecal morphine was superior to non-neuraxial analgesia, in terms of reduced postoperative morphine requirements and the probability of adequate pain control in patients who underwent upper gastrointestinal surgery. However, the benefits of thoracic epidural analgesia and intrathecal morphine were not sustained across all time periods regarding control of pain. The study is limited by its retrospective design, heterogenous group of upper gastrointestinal surgeries and confounding by indication.

**Supplementary Information:**

The online version contains supplementary material available at 10.1186/s13741-023-00324-0.

## Background

Opioids are a mainstay of perioperative pain management, but opioid-related side effects may limit dosing and negatively impact on recovery. Furthermore, in the context of an opioid epidemic and abuse crisis, measures to minimise opioid use following hospital discharge is a priority (Glare et al. 2019).

Epidural analgesia is the traditional gold standard for pain management following major abdominal surgery and continues to be recommended in Enhanced Recovery After Surgery (ERAS) protocols following upper gastrointestinal surgery (Popping et al. 2014; Hughes et al. 2014; Shi et al. 2014; Melloul et al. 2020; Low et al. 2019). Amongst Fellows of the Australian and New Zealand College of Anaesthetists surveyed, epidural analgesia is experiencing a decline in popularity relative to intrathecal morphine (Pirie et al. 2020). Difficulty with thoracic epidural insertion, high failure rates, side effects in the context of limited high-quality evidence of patient benefit, have likely contributed to this decline (Rigg et al. 2002; Groen et al. 2019; Virlos et al. 2010). Intrathecal morphine is an attractive alternative (Tang et al. 2020; Koning et al. 2020). A reduction in measured postoperative pain and an opioid-sparing effect of intrathecal hydrophilic opioids in abdominal surgery has been reported, thereby warranting a comparative analysis of its efficacy and utility relative to epidural analgesia (Koning et al. 2020; Dichtwald et al. 2017; Roy et al. 2006; Ko et al. 2009). Direct comparisons of intrathecal morphine and thoracic epidural analgesia in upper gastrointestinal surgery have reported lower opioid consumption and improved pain control in the epidural analgesia groups, but at the expense of greater hypotension, vasopressor and intravenous fluid use (Lee et al. 2014; Pietri et al. 2006; Kasivisvanathan et al. 2014; Sakowska et al. 2009; Koea et al. 2009).

In laparoscopic abdominal surgery, intrathecal morphine has demonstrated opioid-sparing effects and effective postoperative analgesia (Kong et al. 2002; Wongyingsinn et al. 2012; Pirie et al. 2022) whilst epidural analgesia has not demonstrated clear benefits compared with systemic analgesia (Liu et al. 2014; Khan et al. 2013). As traditionally open major abdominal operations transition to laparoscopic approaches, an assessment of the benefit in transitioning analgesic techniques should logically follow and is merited. We therefore explored the impact of intrathecal morphine, thoracic epidural analgesia and non-neuraxial analgesia techniques on opioid consumption and postoperative pain scores in contemporary upper gastrointestinal surgical practice, with both laparoscopic and open surgical approaches. We hypothesised that compared to parenteral opioids, intrathecal morphine will be opioid sparing in open and laparoscopic UGI surgery and demonstrate a comparative and acceptable pain profile when compared to epidural analgesia.

## Methods

Following ethics committee approval (Northern Sydney Local Health District Human Research Ethics Committee 2020/ETH02058) we undertook a retrospective cohort analysis in patients who underwent upper gastrointestinal surgery over a 5-year period (between November 2015 and October 2020) at Royal North Shore Hospital, a 900-bed tertiary facility, in Sydney, Australia. Billing codes were used to identify patients within the electronic medical record to include those who had elective open or laparoscopic upper gastrointestinal procedures including: hepatectomy, gastrectomy, pancreatectomy, oesophagectomy, pancreatoduodenectomy, splenectomy, diaphragmatic or hiatus hernia repair, or bariatric surgery.

### Definitions

For our primary endpoints, pain scores were recorded using the 11-point numerical rating scale where zero indicates no pain, and 10 the worst pain imaginable (Hawker et al. 2011). Oral morphine equivalent doses were calculated using the Australian and New Zealand College of Anaesthetists and Faculty of Pain Medicine opioid equianalgesic calculator (Faculty of Pain Medicine A. 2019). The intraoperative opioid calculation did not include morphine administered intrathecally.

Length of hospital stay was represented in days and measured from the day of surgery to the day of discharge. Length of high dependency care admission was also recorded to the nearest whole day. Hypotension was defined by the requirement for a vasopressor infusion to maintain blood pressure parameters postoperatively. Use of anti-emetics was used to identify the occurrence of nausea and vomiting. Respiratory depression was defined as a respiratory rate less than eight breaths per minute, pulse oximeter oxygen saturations less than 92%, initiation of non-invasive ventilation or intubation, or administration of naloxone. Opioid induced sedation was assumed if naloxone was administered. The presence of an ileus or urinary retention was determined by free-text assessment of the discharge summary.

In recognition of the broad inclusion criteria for “major” upper abdominal surgeries, the authors classified procedures into three groups based on perceived extent of surgical insult: type 1: extensive major (liver resections, splenectomy, distal pancreas resection, gastric resection), type 2: most extensive major (pancreato-duodenectomy, oesophagectomy) or type 3: other major (radical gallbladder resection, diaphragmatic and hiatus hernia repair, bariatric surgery).

### Key outcomes

The primary outcomes for this study were differences in pain scores and daily cumulative oral morphine equivalent dose until 72 h postoperatively for the patients grouped into either thoracic epidural analgesia, intrathecal morphine, or non-neuraxial analgesia cohorts. A difference of 10 mg oral morphine equivalent consumption was considered clinically relevant. Pain scores of four or less were considered to reflect adequate pain control (Serlin et al. 1995).

Secondary outcomes explored the difference across the analgesic groups with regards to incidence of hypotension, urinary retention, nausea and vomiting, respiratory depression, sedation, duration of intensive care and overall hospital stay, opioid prescription at discharge, and in-patient mortality.

### Data extraction

Data were extracted from the hospital electronic medical record (Cerner Millennium and MetaVisionSuite databases) and entered into a REDCap database hosted at Northern Sydney Local Health District. Preoperative data collected included: age, sex, American Society of Anesthesiologists (ASA) physical status, timing of surgery, and surgery type. Intraoperative data collected included: surgical approach (open or laparoscopic), analgesic technique, dose of intrathecal morphine (if used), ease of neuraxial placement (if used and indicated), dose and types of intraoperative analgesia, and extubation/ventilation status at conclusion of the surgical procedure. Postoperative data included: patient disposition, duration of high dependency unit stay, worst resting and dynamic pain scores in the recovery unit as well as on postoperative days 0 to 3 recorded by the ward nurses, oral morphine equivalent dose administered while in the recovery unit, daily cumulative oral morphine equivalent dose until postoperative day 3, obtained from the PCA and medication chart, duration of hospital stay, opioid prescription on discharge home, and occurrence of opioid related side effects including: itch, nausea and/or vomiting requiring treatment, sedation or respiratory depression requiring naloxone or airway intervention, urinary retention, ileus, hypotension requiring fluids or vasopressor, or death having occurred prior to the date of data extraction.

### Statistical methodology

Our detailed statistical analysis approach is included in the additional material. In brief, post-operative morphine requirements, resting pain scores and dynamic pain scores over the 4 post-operative time points were modelled separately with Bayesian generalised mixed effect models. In this retrospective cohort sample, each patient can have up to 4 data points across the post-operative period. With 427 patients, the data set had 1664, 1548 and 1252 data points for postoperative morphine requirement, resting pain score and dynamic pain scores, respectively, that are potentially highly correlated. The generalised mixed effect model was chosen as the modelling approach as it allows within-patient, across time-points correlations to be accounted for.

Our modelling approach allows bespoke specifications to the models according to what we understand about the properties of a particular outcome that we are interested in. For example, for postoperative morphine requirements, we would expect it to be highly skewed and its distribution to be not normal. We would also expect the morphine requirements over the postoperative period to be non-linear. From clinical experience, with different intra-operative analgesic techniques and with different surgical extent and approaches, we would also expect that some patients may not need opioid at all postoperatively. This may give rise to a bi-modal distribution of postoperative morphine where there is a second peak of an excess of zero opioid dosing. These more realistic properties of morphine requirements have been incorporated in our modelling by specifying a hurdle-lognormal link and using natural splines.

Pain scores, on the other hand, are arguably not numerical scores that can be added, subtracted or averaged between different patients. They are ordinal values where the interval between a pain score of 0 to 3 is not necessarily the same as a pain score difference between 6 and 9, but a pain score of 0 is considered less than a pain score of 1, and a pain score of 1 < 2 < 3…. < 10. We have thus chosen to specify our resting and dynamic pain score mixed effect models with an ordinal cumulative link.

The adjusting variables were collected a priori*.* Two kinds of adjusting variables were included—those intrinsic to the patient such as age, gender, and their ASA physical status classification, and those extrinsic to the patient such as the surgical procedure conducted, and the surgical approach chosen. We are interested in how these “extrinsic” variables may affect the opioid requirements or the pain scores over the postoperative period, hence in our modelling approach we have included additional interaction terms against time.

In addition to the modelling specifications, we chose to analyse our data within the Bayesian statistical framework. Bayesian statistics is a particular approach to applying probability to statistical problems. It is a different approach to the more commonly seen “Frequentist” or null hypothesis significance testing (NHST) framework that use a number of tools for interpretation such as *p* values, confidence intervals and the concepts of type I/II errors (Greenland et al. 2016). In light of the recent concerns and controversies regarding mis-interpretations from null hypothesis significance testing approaches (Amrhein et al. 2019; McShane et al. 2009), and with advances in computation, Bayesian analysis has increasingly gained popularity (Bittl and He 2017; Frost et al. 2021; Goligher et al. 2018; Sidebotham et al. 2021; Zampieri et al. 2020). In short, with the model structure we developed from our understanding of the data generating process (the likelihood), such as that discussed above for morphine use across the postoperative time-period, a Bayesian approach can further incorporate what was already known (the prior distribution), combining with current evidence as support (the data we collect), to arrive in posterior probability distributions that we can then use to interpret the results. There are a number of benefits of using this approach (Dunson 2001; Kelter 2020; Kruschke and Liddell 2018). For our analysis in particular, this allows modelling with realistic opioid requirements and pain score values, provide direct probability interpretations. Recent advances in Bayesian software also allowed easy computation for these complex models. The *brms* package in R and *Stan* software provided the necessary computation. Our model development and evaluation approach were consistent with contemporary Bayesian workflow and reporting practice (Gabry et al. 2019; Kruschke 2021). Further details of the analysis, software packages used, and their versions are included in Additional file 1a, b, and c.

For postoperative morphine requirements (Additional file 1a), our analysis was interested in the differences in postoperative oral morphine equivalent dose requirements between the different analgesic techniques in the upper gastrointestinal surgical cohorts. To assess this difference, we first predict from the model the posterior predictive distribution of their oral morphine equivalent requirements for patients receiving a particular analgesic technique. We then set out to predict a posterior predictive distribution of oral morphine requirements for the same group of patient characteristics with a different analgesic technique. The difference between these two posterior predictive distributions yields a distribution reflective of the morphine requirement difference between the two analgesic techniques. We compare different surgical approaches (open and laparoscopic) across the specific operation categories (type 1 to 3) for an effect correlation and considered a difference of more than 10 mg of oral morphine equivalents to be clinically significant.

The oral morphine equivalent dose difference is calculated as the analgesic requirement for the original technique minus the opioid consumption from receiving an alternate analgesic technique. The posterior predictive differences for “non-neuraxial to intrathecal morphine” and “intrathecal morphine to non-neuraxial” will not be just the reverse of the sign, because the baseline characteristics and other covariates of those that received non-neuraxial analgesia may differ from the baseline characteristics and covariates of those who received intrathecal morphine.

We followed the same procedure in making predictions from the models separately for the postoperative resting and dynamic pain control (Additional file 1b and c).

## Results

Data were extracted from the electronic medical records of 427 patients who underwent open (*n* = 300) and laparoscopic (*n* = 127) upper gastrointestinal surgery at the Royal North Shore Hospital between November 2015 and October 2020. Most patients were ASA physical status 2 or 3 across all cohorts (Table 1).

**Table 1:** Patient characteristics

	Non-neuraxial (*n* = 168)	Intrathecal Morphine (*n* = 79)	Thoracic epidural Analgesia (*n* = 180)
Age (years)	58 (44 – 67)	62 (50—71)	65 (56 – 71)
Female sex	94 (56%)	41 (52%)	76 (42%)
American Society of Anesthesiologists Physical Status			
1	5 (42%)	3 (25%)	4 (33%)
2	51 (44%)	18 (16%)	46 (40%)
3	101 (36%)	57 (20%)	121 (43%)
4	11 (52%)	1 (5%)	9 (43%)
Surgical approach			
Laparoscopic	102 (85%)	14 (12%)	4 (3%)
Open	63 (21%)	63 (21%)	174 (58%)
Laparoscopic converted to open	3 (43%)	2 (29%)	2 (29%)
Surgery Type Based on Extent			
Type 1: extensive major surgery	75 (34%)	59 (27%)	88 (40%)
liver resections, splenectomy, distal pancreas resection, gastric resection			
Type 2: most extensive major surgery	5 (5%)	12 (12%)	87 (84%)
pancreato-duodenectomy, esophagectomy			
Type 3: major surgery	88 (87%)	8 (8%)	5 (5%)
diaphragmatic hernia repair, bariatric surgery, hiatus hernia repair, resection gallbladder fossa			

Median (IQR); n (%)

Neuraxial techniques for postoperative analgesia were more common in open surgical approaches, thoracic epidural (58%) and intrathecal morphine (21%), versus non-neuraxial (21%). By contrast, 85% of patients who underwent laparoscopic surgery had non-neuraxial analgesic management. The predominance of patients undergoing pancreatoduodenectomy (84%) or oesophagectomy (96%) received thoracic epidural analgesia. Most patients (87%) undergoing less extensive procedures, such as hiatus hernia repairs, were more likely to receive non-neuraxial analgesia as their primary analgesic modality, and rarely received intrathecal morphine (8%) or thoracic epidural analgesia (5%) (Table 1).

For thoracic epidural analgesia, 0.1% bupivacaine was co-administered with 2 mcg/mL of fentanyl and adrenaline administered as an adjunct. Epidural infusion used a combination of programmed intermittent epidural boluses and patient controlled epidural analgesia. For spinal analgesia, intrathecal morphine was co-administered with local anaesthetic without other additives.

Intraoperative oral morphine equivalent dose was highest in the intrathecal morphine group, median, interquartile range (IQR) 98 (60–120) mg, compared to the non-neuraxial 50 (IQR 30–100) mg and thoracic epidural 20 (IQR 20–60) mg analgesia groups (Table 2). More than 90% of patients who received a neuraxial technique had a planned high dependency unit admission postoperatively (92% intrathecal morphine, 93% thoracic epidural analgesia) compared to 57% of patients in the non-neuraxial cohort.

**Table 2:** Perioperative outcomes

	Non-neuraxial (*n* = 168)	Intrathecal morphine (*n* = 79)	Thoracic epidural analgesia (*n* = 180)
Intraoperative oral morphine equivalent dose, median (IQR) mg	50 (30–100)	98 (60–120)	20 (20–60)
Intraoperative intrathecal morphine dose, median (IQR) mcg	–	350 (150–500)	–
Local anaesthetic wound infusion devices (e.g. Painbuster™)	30 (18%)	17 (22%)	
Regional local anaesthetic block	13 (8%)	1 (1%)	
Intraoperative NSAIDs (COX-1/2 inhibitors)	71 (42%)	22 (28%)	21 (12%)
Intraoperative ketamine	55 (33%)	32 (41%)	12 (7%)
High dependency unit admission			
No	71 (42%)	5 (6%)	10 (6%)
Planned	95 (57%)	73 (92%)	168 (93%)
Unplanned	2 (1%)	1 (1%)	2 (1%)
Admitted to high dependency unit intubated	24 (14%)	13 (16%)	14 (8%)
High dependency unit length of stay (days)	1 (0–1)	1 (1–2)	3 (2–4)
Adverse events			
Respiratory depression	9 (5%)	7 (9%)	10 (6%)
Postoperative nausea and vomiting	93 (55%)	49 (62%)	123 (68%)
Sedation	1 (1%)	6 (8%)	4 (2%)
Hypotension	34 (20%)	24 (30%)	113 (63%)
Ileus	2 (1%)	8 (10%)	13 (7%)
Urinary retention	3 (2%)	0 (0%)	2 (1%)
Mortality			
In hospital	0 (0%)	1 (1%)	3 (2%)
At time of data extraction (up to 5 years following surgery)	22 (13%)	13 (16%)	35 (19%)
Hospital length of stay (days)	5 (3–8)	8 (6–12)	13 (9–22)
Opioid on discharge	90 (54%)	42 (54%)	79 (44%)
Neuraxial details			
Thoracic epidural attempted but unable to insert	2	9	
Epidural inserted first pass			129 (72%)
>1 attempt to insert epidural			50 (28%)
Epidural used as planned			100 (83%)
Epidural discontinued early due to poor function			45 (25%)
Epidural discontinued early due to other reasons			12 (7%)
Intrathecal injection > 1 attempt to site		15 (19%)	
Intrathecal injection attempt but unable		(0%)	

Median (IQR); *n* (%)

Inability to site an epidural catheter occurred in 11 patients planned for thoracic epidural analgesia, 9 of whom subsequently successfully received an intrathecal morphine injection (Table 2). More than one attempt at siting the thoracic epidural catheter occurred in 28% of cases. All patients planned for an intrathecal morphine injection had such, however, 19% of patients required more than one attempt to access the intrathecal space. The median (range) dose of intrathecal morphine was 350 (150–500) mcg. Epidural analgesia was ceased earlier than planned in 32% of cases, mainly because of inadequate epidural function (25%). Analgesic adjuncts, including non-steroidal anti-inflammatory agents (COX-1 or -2 inhibitors), ketamine, regional blocks or elastomeric local anaesthetic wound infusion devices (Painbusters™), were utilised more frequently in the non-neuraxial and intrathecal morphine groups than in the thoracic epidural group (Table 2).

### Primary outcome: oral morphine equivalent dose

#### Non-neuraxial versus intrathecal groups

Simulation of patient allocation from the non-neuraxial group to the intrathecal morphine group predicts no *overall* clinically significant difference in oral morphine consumption for both laparoscopic (− 5.0 mg [− 18.3 to 9.3]) and open (− 6.0 mg [− 31.9 to 22.4]) surgeries (Table 3 and Additional file 1a Figure S18), reflecting a probability of reducing the oral morphine equivalent dose requirement by at least 10 mg (Pr(Diff $\le -10 \mathrm{mg})$) of 22% and 38%, respectively.

**Table 3:** Predicted median oral morphine equivalent dose (mg)

Direction of change in analgesia	Subgroup of surgery	Median difference in oral morphine equivalent dose (mg)	95% Credible Interval	Probability	
				Increase in median OMED $\ge 10mg$	Reduction in median OMED $\ge 10mg$
Non-neuraxial to intrathecal morphine	Laparoscopic	-5.0	-18.3 to 9.3	0.03	0.22
	Open	-6.0	-31.9 to 22.4	0.14	0.38
	Type 1 surgery	-6.2	-31.1 to 20.0	0.11	0.38
	Type 2 surgery	-2.1	-51.3 to 47.5	0.29	0.36
	Type 3 surgery	-4.6	-18.6 to 9.5	0.03	0.22
Intrathecal morphine to non-neuraxial	Laparoscopic	3.5	-22.8 to 33.8	0.31	0.17
	Open	4.4	-23.2 to 37.5	0.37	0.16
	Type 1 surgery	4.1	-21.4 to 30.4	0.33	0.14
	Type 2 surgery	7.1	-53.2 to 71.5	0.46	0.28
	Type 3 surgery	3.9	-51.6 to 64.3	0.41	0.28
Non-neuraxial to thoracic epidural analgesia	Laparoscopic	-38.5	-47.2 to -29.4	0.00	1.00
	Open	-67.2	-86.3 to -47.2	0.00	1.00
	Type 1 surgery	-63.1	-82.0 to -46.4	0.00	1.00
	Type 2 surgery	-48.5	-99.6 to -11.8	0.00	0.98
	Type 3 surgery	-37.6	-46.2 to -28.6	0.00	1.00
Thoracic epidural analgesia to non-neuraxial	Laparoscopic	27.1	5.8 to 58.6	0.94	0.00
	Open	66.3	41.5 to 97.3	1.00	0.00
	Type 1 surgery	61.3	38.8 to 90.4	1.00	0.00
	Type 2 surgery	68.6	37.1 to 107.7	1.00	0.00
	Type 3 surgery	51.8	5.1 to 128.0	0.97	0.00
Intrathecal morphine to thoracic epidural analgesia	Laparoscopic	-41.8	-66.2 to -20.1	0.00	1.00
	Open	-66.4	-87.9 to -47.0	0.00	1.00
	Type 1 surgery	-56.6	-74.4 to -38.5	0.00	1.00
	Type 2 surgery	-84.3	-141.2 to -35.2	0.00	1.00
	Type 3 surgery	-61.1	-115.4 to -15.6	0.00	0.99
Thoracic epidural analgesia to intrathecal morphine	Laparoscopic	21.7	0.4 to 50.4	0.87	0.00
	Open	62.7	40.5 to 92.5	1.00	0.00
	Type 1 surgery	57.8	35.0 to 86.2	1.00	0.00
	Type 2 surgery	64.8	35.5 to 100.7	1.00	0.00
	Type 3 surgery	48.9	-2.9 to 128.0	0.96	0.00

Probability $\ge$ 10 mg refers to the probability of a difference in the predicted oral morphine equivalent dose of more than or equal to 10 mg occurring had the alternative analgesia option been utilised, where 0.00 denotes almost no chance of a difference, and 1.00 indicates almost certain chance there will be such a difference i.e. in the first row, if a patient had been administered intrathecal morphine instead of a non-neuraxial technique, they would have reduced their opioid consumption by a mean difference of 5 mg within a range (credibility index) of -18.3 to 9.3 mg and a 3% probability of increasing their opioid consumption by $\ge$ 10 mg and 22% probability of reducing it by $\ge$ 10 mg

However, specific to postoperative day 0, the probability of a clinically relevant reduction in opioid consumption for laparoscopic surgery was 92% (− 20.4 mg [− 35.3 to − 5.9], Pr(Diff $\ge -$ 10 mg) = 0.92) and 87% for open surgery (− 20.5 mg [− 40.0 to − 3.6], Pr(Diff $\ge -$ 10 mg) = 0.87). By postoperative day 2 and 3, the trend appeared to have reversed in favour of non-neuraxial analgesia, although the median doses and their 95% credible intervals remained clinically not significant (Table 3).

Similar patterns were observed in the subgroup analysis by surgical types (Table 3 and Additional file 1a Figure S19). There was no *overall* clinically relevant reduction in oral morphine equivalent consumption if one were to have received intrathecal morphine instead of non-neuraxial analgesia, being − 6.2 mg (− 31.1 to 20.0, Pr(Diff $\le -10 \mathrm{mg}$) = 0.38), − 2.1 mg (− 51.3 to 47.5, Pr(Diff $\le -10\mathrm{ mg}$) = 0.36) and − 4.6 mg (− 18.6 to 9.5, Pr(Diff $\le - 10 \mathrm{mg}$) = 0.22) for type 1, 2 and 3 surgeries, respectively. Clinically relevant reduction in opioid requirement was evident on postoperative day 0 for type 1 [− 21.5 mg (− 38.5 to − 3.5, Pr(Diff $\le -10 \mathrm{mg}$) = 0.90] and type 3 [− 19.8 mg (− 36.1 to − 5.9, Pr(Diff $\le -10 \mathrm{mg}$) = 0.91] surgeries.

#### Thoracic epidural analgesia versus non-neuraxial groups

The probability of clinical significance in favour of thoracic epidural analgesia was greater than 97% for all analyses. *Overall*, movement from non-neuraxial to thoracic epidural analgesia resulted in a median oral morphine equivalent opioid reduction of 38.5 mg (− 47.2 to − 29.4, Pr(Diff $\le -10\mathrm{ mg}$) = 1.00) in laparoscopic surgery and 67.2 mg (− 86.3 to − 47.2, Pr(Diff $\le -10 \mathrm{mg}$) = 1.00 in open surgery (Table 3 and Additional file 1a Figure S18). This effect was sustained for all postoperative time points, with postoperative day 1 predicting the greatest reduction in opioid consumption if one were to receive thoracic epidural analgesia instead of a non-neuraxial technique for both laparoscopic (− 59.5 mg (− 80.2 to − 43.6), Pr(Diff $\le -10 \mathrm{mg}$) = 1.0) and open (− 105.5 mg (− 145.0 to − 67.4), Pr(Diff $\le - 10 \mathrm{mg}$) = 1.00) surgeries.

Subgroup analysis based on the extent of surgery determined an *overall* clinically relevant reduction in median oral morphine equivalent dose across all groups if one switched the analgesic technique from non-neuraxial to thoracic epidural analgesia (Table 3 and Additional file 1a Figure S19), specifically − 63.1 mg (− 82.0 to − 46.4, Pr(Diff $\le - 10\mathrm{ mg}$) = 1.00), − 48.5 mg (− 99.6 to − 11.8, Pr(Diff $\ge \le - 10 \mathrm{mg}$) = 0.98) and − 37.6 mg (− 46.2 to − 28.6, Pr(Diff $\le - 10 \mathrm{mg}$) = 1.00) for type 1, 2, and 3 surgeries, respectively. Again the effect was evident at all postoperative time points, with the greatest impact on postoperative day 1 (type 1: − 101.0 mg (− 138.7 to − 67.3), Pr(Diff $\le -10 \mathrm{mg}$) = 1.0; type 2: − 65.9 mg (− 152.5 to − 6.5), Pr(Diff $\le - 10\mathrm{ mg}$) = 0.98; type 3: − 56.2 mg (− 74.3 to − 37.1), Pr(Diff $\le - 10\mathrm{ mg}$) = 1.0) with the effect gradually attenuating by postoperative day 3 (type 1: − 42.7 mg (− 78.5 to − 9.3), Pr(Diff $\le - 10\mathrm{ mg}$) = 0.97; type 2: − 37.5 mg (− 132.1 to 57.4), Pr(Diff $\le - 10\mathrm{ mg}$) = 0.79; type 3: − 25.2 mg (− 39.8 to − 11.0), Pr(Diff $\le - 10\mathrm{ mg}$) = 0.98).

#### Intrathecal versus epidural groups

Simulation of patient allocation from the intrathecal morphine to epidural analgesia group found an *overall* reduction in median opioid requirements in both laparoscopic [− 41.8 mg (− 66.2 to − 20.1, Pr(Diff $\le - 10 \mathrm{mg}$) = 1.00)] and open surgery [− 66.4 mg (− 87.9 to − 47.0, Pr(Diff $\le -10 \mathrm{mg}$) = 1.00)](Table 3 and Additional file 1a Figure S18). This effect was least prominent on postoperative day 0 for both surgical approaches, laparoscopic [− 9.3 mg (− 28.8 to 10.8, Pr(Diff $\le - 10 \mathrm{mg}$) = 0.47)] versus open [− 16.1 mg (− 31.3 to − 2.6, Pr(Diff $\le - 10 \mathrm{mg}$) = 0.83)], the former of which was not clinically significant. From postoperative day 1 onwards, the prominence in the opioid reductions were sustained, possibly reflecting the increased requirements when the intrathecal morphine wears off. This pattern is again observed with subgroup analysis by surgical extent (Table 3 and Supplementary Material 1a Figure S19).

### Primary outcome: rest and dynamic pain scores

**Fig. 1:**
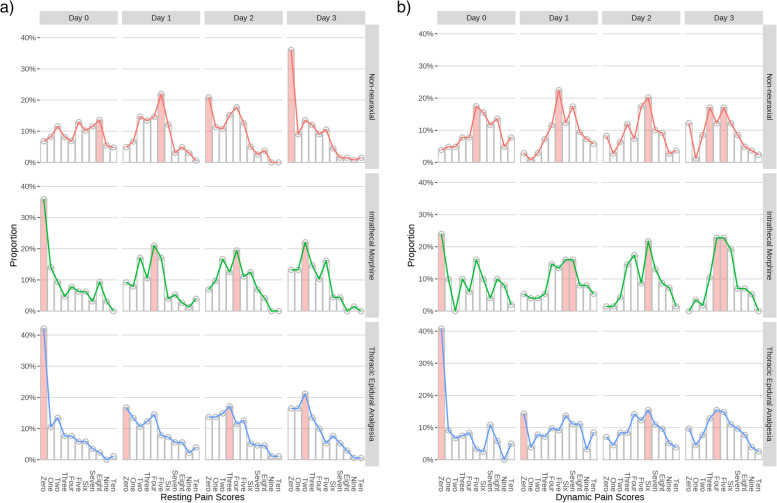
Frequency of recorded **a** rest and **b** dynamic pain scores. Frequency of recorded pain scores over different postoperative days (0 to 3) across three analgesic techniques. The *X* axis denotes the numerical rating scale (0–10), *Y* axis denotes proportion of patients in that group. The red bars represent the pain score with the most counts

The *rest* pain score most frequently measured was 4 or less at all time points in the intrathecal morphine and thoracic epidural analgesia groups (Fig. 1). *Rest* pain scores of greater or equal to 5, were more frequently measured in the non-neuraxial group up until postoperative day 1, after which, the highest frequency pain score in this group was 0. *Dynamic* pain scores were more frequently greater than 5 at all time points in the non-neuraxial group. Early *dynami*c pain scores (postoperative day 0 and day 1) were more frequently 0 for intrathecal morphine and thoracic epidural analgesia, the latter remaining 0 until postoperative day 2. Thereafter, *dynamic* pain scores were more frequently greater than 5 in both the intrathecal morphine and thoracic epidural analgesia groups.

#### Non-neuraxial versus intrathecal morphine group

**Fig. 2:**
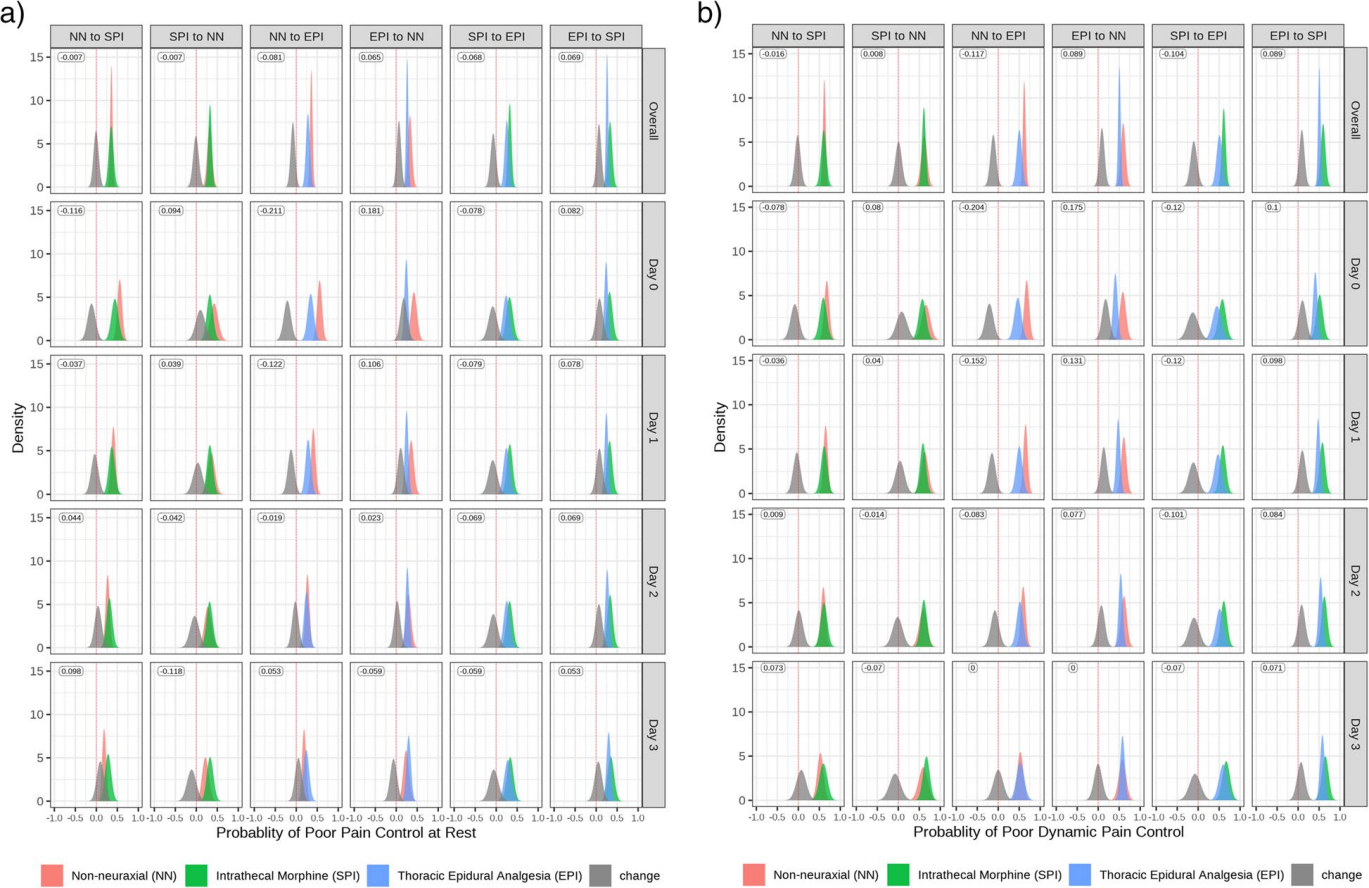
Probability of poor *rest* (**a**) or *dynamic* (**b**) pain control if one were to have a different analgesic technique. The plot shows the posterior predictive distribution of the probability of poor pain control of the original analgesic technique, the posterior predictive distribution of the probability of poor pain control if one were to have a different analgesic technique, and the distribution of the differences in the probability of poor pain control (grey). The black error bars reflect the 95% credible intervals

Overall, simulation of allocation from a non-neuraxial technique to intrathecal morphine, or vice versa, had minimal impact on the probability of poorly controlled *rest* (median change − 1%, 95% credible interval (CrI) − 0.09 to 0.08) and *dynamic* (− 2%, 95% Crl − 0.11 to 0.08) pain, according to extent of surgery or surgical approach (Tables 4 and 5, Fig. 2). A 12% (− 0.26 to 0.01) reduction in the probability of poor *rest* pain control was evident on postoperative day 0 and 4% reduction (− 0.16 to 0.09) on postoperative day 1. Reversal in favour of the non-neuraxial group was evident by postoperative day 3. Subgroup analysis by surgical approach and type of surgery had similar results. An early benefit of intrathecal morphine at reducing the probability of poorly controlled *dynamic* pain scores (0–20% reduction) is evident across all types of surgery and approaches, with reversal in favour of non-neuraxial analgesia by postoperative day 3.

**Table 4:** Overall change in probabilities of poor *rest* pain control

Direction of change in analgesia	Subgroup of surgery	Change in probability of poor pain control, median	95% credible interval
Non-neuraxial to intrathecal morphine	Overall	− 0.01	− 0.09 to 0.08
	Laparoscopic	− 0.01	− 0.10 to 0.07
	Open	0.01	− 0.10 to 0.12
	Type 1 surgery	0.00	− 0.10 to 0.10
	Type 2 surgery	0.00	− 0.31 to 0.25
	Type 3 surgery	− 0.01	− 0.10 to 0.08
Intrathecal morphine to non-neuraxial	Overall	− 0.01	− 0.11 to 0.08
	Laparoscopic	0.00	− 0.14 to 0.14
	Open	− 0.01	− 0.11 to 0.09
	Type 1 surgery	− 0.01	− 0.11 to 0.09
	Type 2 surgery	0.00	− 0.20 to 0.20
	Type 3 surgery	0.04	− 0.19 to 0.23
Non-neuraxial to thoracic epidural analgesia	Overall	− 0.08	− 0.16 to -0.01
	Laparoscopic	− 0.09	− 0.16 to -0.01
	Open	− 0.06	− 0.18 to 0.02
	Type 1 surgery	− 0.07	− 0.16 to 0.01
	Type 2 surgery	− 0.06	− 0.31 to 0.19
	Type 3 surgery	− 0.09	− 0.16 to 0.00
Thoracic epidural analgesia to non-neuraxial	Overall	0.06	0.00 to 0.14
	Laparoscopic	0.04	− 0.13 to 0.30
	Open	0.06	− 0.01 to 0.14
	Type 1 surgery	0.06	− 0.03 to 0.15
	Type 2 surgery	0.07	− 0.02 to 0.15
	Type 3 surgery	0.05	− 0.21 to 0.26
Intrathecal morphine to thoracic epidural analgesia	Overall	− 0.07	− 0.16 to 0.02
	Laparoscopic	− 0.05	− 0.20 to 0.07
	Open	− 0.07	− 0.16 to 0.03
	Type 1 surgery	− 0.06	− 0.16 to 0.02
	Type 2 surgery	− 0.07	− 0.28 to 0.10
	Type 3 surgery	− 0.08	− 0.27 to 0.15
Thoracic epidural analgesia to intrathecal morphine	Overall	0.07	− 0.01 to 0.15
	Laparoscopic	0.09	− 0.17 to 0.30
	Open	0.07	0.00 to 0.15
	Type 1 surgery	0.07	− 0.02 to 0.16
	Type 2 surgery	0.07	− 0.01 to 0.16
	Type 3 surgery	0.05	− 0.21 to 0.26

**Table 5:** Probability of poor *dynamic* pain control

Direction of change in analgesia	Subgroup of surgery	Change in probability of poor pain control, median	95% credible interval
Non-neuraxial to intrathecal morphine	Overall	− 0.02	− 0.11 to 0.08
	Laparoscopic	− 0.02	− 0.14 to 0.08
	Open	0.00	− 0.10 to 0.09
	Type 1 surgery	− 0.01	− 0.11 to 0.08
	Type 2 surgery	0.00	− 0.30 to 0.30
	Type 3 surgery	− 0.02	− 0.14 to 0.09
Intrathecal morphine to non-neuraxial	Overall	0.01	− 0.10 to 0.11
	Laparoscopic	0.02	− 0.19 to 0.17
	Open	0.00	− 0.10 to 0.11
	Type 1 surgery	0.00	− 0.10 to 0.12
	Type 2 surgery	0.00	− 0.19 to 0.16
	Type 3 surgery	0.02	− 0.27 to 0.27
Non-neuraxial to thoracic epidural analgesia	Overall	− 0.12	− 0.21 to − 0.02
	Laparoscopic	− 0.14	− 0.24 to − 0.03
	Open	− 0.09	− 0.20 to 0.02
	Type 1 surgery	− 0.10	− 0.20 to − 0.01
	Type 2 surgery	− 0.10	− 0.50 to 0.20
	Type 3 surgery	− 0.13	− 0.24 to − 0.01
Thoracic epidural analgesia to non-neuraxial	Overall	0.09	0.01 to 0.17
	Laparoscopic	0.08	− 0.25 to 0.42
	Open	0.09	0.01 to 0.17
	Type 1 surgery	0.09	0.00 to 0.18
	Type 2 surgery	0.08	− 0.01 to 0.18
	Type 3 surgery	0.12	− 0.19 to 0.38
Intrathecal morphine to thoracic epidural analgesia	Overall	− 0.10	− 0.22 to 0.00
	Laparoscopic	− 0.12	− 0.29 to 0.06
	Open	− 0.10	− 0.22 to 0.01
	Type 1 surgery	− 0.10	− 0.21 to 0.01
	Type 2 surgery	− 0.11	− 0.27 to 0.11
	Type 3 surgery	− 0.14	− 0.36 to 0.14
Thoracic epidural analgesia to intrathecal morphine	Overall	0.09	0.01 to 0.19
	Laparoscopic	0.08	− 0.25 to 0.42
	Open	0.09	0.00 to 0.17
	Type 1 surgery	0.09	− 0.01 to 0.18
	Type 2 surgery	0.09	− 0.02 to 0.19
	Type 3 surgery	0.12	− 0.19 to 0.38

#### Non-neuraxial versus thoracic epidural analgesia group

Where a non-neuraxial technique is chosen, simulation of movement to thoracic epidural analgesia is predicted to reduce the median probability of poorly controlled *rest* pain overall by 8% (-0.16 to -0.01), with the greatest impact on day 0 (− 21% [− 0.35 to − 0.10]) (Fig. 2 and Table 4). No difference in the probability of poorly controlled *rest* pain was calculated from postoperative day 2 onwards following pancreato-duodenectomy and oesophagectomy, and by postoperative day 3, an increase was calculated following laparoscopic (5% [− 0.06 to 0.17]) and open (6% [− 0.14 to 0.22]) surgery. This finding is similar when the movement between analgesia types is considered in reverse.

A 12% reduction (− 0.21 to − 0.02) in the overall probability of poorly controlled *dynamic* pain was found when moving from non-neuraxial to thoracic epidural analgesia (Fig. 2 and Table 5). The impact is most substantial on postoperative day 0 (20% reduction [− 0.35 to − 0.08]) and predicted to continue until postoperative day 2. Similar findings in favour of thoracic epidural analgesia are evident when moving from thoracic epidural analgesia to non-neuraxial management, and when separating groups by surgical approach. Analysis based on extent of surgery found that by postoperative day 3 there was no difference in the probability of poorly controlled *dynamic* pain evident between the analgesic techniques for type 1 surgeries. There is an early reduction in the probability of poorly controlled *dynamic* pain when moving from non-neuraxial to thoracic epidural analgesia (33% [− 0.67 to − 0.33)] following pancreato-duodenectomy and oesophagectomy, however, there is no difference from postoperative day 1 onwards. When calculating the reverse transition from thoracic epidural to non-neuraxial, the benefit remains until postoperative day 2. Similar findings are seen for type 3 surgery.

#### Intrathecal morphine versus thoracic epidural analgesia groups

Simulation of allocation of patients from the intrathecal morphine to thoracic epidural analgesia is predicted to reduce the probability of median poorly controlled *rest* pain by 7% overall (− 0.16 to 0.02), whilst increasing it by 7% when the reverse is considered (− 0.01 to 0.15) (Fig. 2 and Table 4). In the least extensive category of major surgery (classed as type 3), there was no predicted difference in the probability of poorly controlled *rest* pain when moving from thoracic epidural analgesia to intrathecal morphine. A consistent benefit (ranging from 0 to 17%) in favour of thoracic epidural analgesia was seen until postoperative day 2 when considering the reverse movement between analgesia groups.

The probability of overall median poorly controlled *dynamic* pain is − 10% (− 0.22 to 0.00) when simulating movement from intrathecal morphine to thoracic epidural analgesia, with consistent findings until postoperative day 3 and when simulating the reverse movement, from thoracic epidural analgesia to intrathecal morphine (Fig. 2 and Table 5). Subgroup analysis shows the benefit in favour of thoracic epidural analgesia is consistent in magnitude across all types of surgery.

### Secondary outcomes

The incidence of respiratory depression, sedation, and ileus were highest in the intrathecal morphine group (9%, 8%, and 10% respectively) compared to the thoracic epidural analgesia (6%, 2%, and 7%) and non-neuraxial groups (5%, 1%, and 1%) (Table 2). Postoperative nausea and vomiting were prevalent across groups; non-neuraxial (55%), intrathecal morphine (62%), and thoracic epidural analgesia (68%) (Table 2). Hypotension occurred more frequently in the thoracic epidural analgesia group (63%), compared to intrathecal morphine (30%) and the non-neuraxial group (20%).

A greater proportion of patients in the neuraxial cohorts were admitted to the high dependency unit postoperatively, thoracic epidural analgesia (94%), intrathecal morphine (93%), non-neuraxial (58%), where they also remained for longer. The median (IQR) high dependency unit length of stay for thoracic epidural analgesia was 3 (2–4) days, intrathecal morphine was 1 (1–2) day, non-neuraxial analgesia was 1 (0–1) days (Table 2). Overall hospital length of stay was also longest in the thoracic epidural analgesia cohort (epidural analgesia 13 (9–22) days versus intrathecal morphine 8 (6–12) days versus non-neuraxial 5 (3–8) days). In-hospital mortality was less than 2% across all cohorts. Discharge home with opioids was required in 44% (thoracic epidural analgesia) and 54% (intrathecal morphine and non-neuraxial groups) of patients (Table 2). At the time of data extraction, mortality over the 5-year period was highest in the thoracic epidural analgesia group (19%), compared to the intrathecal morphine (16%) and non-neuraxial groups (13%).

## Discussion

In this single institution experience, thoracic epidural analgesia was the preferred technique for open surgical approaches and was utilised in the vast majority of pancreatoduodenectomy and oesophagectomy cases. Intrathecal morphine and non-neuraxial techniques were more common in hepatic resections compared to thoracic epidural analgesia, likely the result of concerns regarding coagulopathy in the postoperative period despite recent evidence of normal or prothrombotic parameters on thromboelastography following hepatic surgery (Weinberg et al. 2011; Oo et al. 2020). Nearly all patients in the neuraxial analgesia groups had a planned admission to the high dependency unit postoperatively, which likely reflects the more extensive surgery they underwent compared to the non-neuraxial cohort, in addition to institutional requirements for managing thoracic epidural analgesia or monitoring for side effects following neuraxial techniques. Overall, the co-primary outcomes of opioid consumption and pain control favoured the use of thoracic epidural analgesia.

The disproportionate use of a thoracic epidural analgesia for more extensive surgery and patients with poorer ASA physical status has obvious implications for correlating differences in outcome, including but not limited to duration of high dependency unit stay, overall hospital length of stay and mortality.

A similar percentage of patients in the non-neuraxial group and intrathecal morphine group were admitted intubated to the intensive care unit, both of which were greater than evident in the thoracic epidural analgesia group despite the heightened complexity of cases in this cohort. The potential reasons for this difference are numerous, including oversedation due to opioid side effects, lack of confidence in the analgesia provided in an institution where epidural analgesia is strongly preferred, patient haemodynamic instability or even inability to adequately control pain. Patient and health economic related flow-on effects need to be considered given this disparity.

Consistent with previous research (Groen et al. 2019; Hermanides et al. 2012), practical concerns associated with the use of thoracic epidural analgesia were evident in this study. Inability, or multiple attempts, to successfully site a thoracic epidural catheter, as well as early cessation due to epidural malfunction or clinical concerns were apparent. Furthermore, hypotension affected more patients with thoracic epidural analgesia compared to those in the intrathecal morphine or non-neuraxial groups, which is likely due to the vasodilatory effect of sympathectomy secondary to the epidural local anaesthetic infusion. Defining hypotension as blood pressure management requiring intravenous fluids and/or a vasopressor ensured that we identified a clinically significant degree of hypotension that involved escalation in care.

Unexpectedly, the amount of intraoperative opioid equivalent dose was higher in the intrathecal morphine group compared to the non-neuraxial group. The greater proportion of open procedures and complexity of surgery requiring postoperative admission to the high dependency unit in the intrathecal morphine group likely serve as a confounder and could account for this finding. Lack of confidence in an equivalent efficacy from intrathecal morphine in an institution which favours the use of thoracic epidural analgesia for major upper gastrointestinal surgery, combined with a planned transfer of patients to the high dependency unit intubated and ventilated, may influence the amount of intraoperative opioids administered, with more liberal doses subsequently given. Aside from this confounding by indication, the analgesic adjuncts (e.g. ketamine and Cox 1 or 2 inhibitors) were used more frequently in the non-neuraxial group, which may also have contributed to this observed difference in opioid requirements.

Simulation of the impact of movement to thoracic epidural analgesia, from either intrathecal morphine or non-neuraxial analgesia, resulted in a clinically relevant reduction in oral median morphine equivalent dose with narrow credible intervals. This finding was consistent irrespective of surgical approach, postoperative day, or extent of surgery and similarly increased oral median morphine equivalent required when we simulated movement in the opposite direction, from thoracic epidural analgesia to the alternative analgesic techniques. The overall probability of reaching a predefined clinically significant difference in median oral morphine equivalent dose greater than 10 mg in favour of thoracic epidural analgesia was consistently high, ranging between 88–100%, and greater than 75% when analysing based on extent of surgery at all time points. The only variation from this was during the early postoperative period following laparoscopic surgery, where the probability in favour of intrathecal morphine finding a difference in median morphine equivalent dose requirements greater than 10 mg ranged between 46 and 47%.

Whilst overall there was no clinically significant difference in opioid consumption between non-neuraxial analgesia and intrathecal morphine, the probability of reaching pre-defined clinical significance in the early postoperative period (up to the end of postoperative day 1) was more than 88%, reflecting the efficacy of intrathecal morphine, albeit its finite duration of action, and warrants consideration of mechanisms or analgesic techniques to extend its duration of effect. The change is similar regardless of the surgical approach and surgery categories.

We found patients most frequently had adequate *rest* pain control until postoperative day 3, and *dynamic* pain control until postoperative day 2 in both thoracic epidural analgesia and intrathecal morphine groups. Despite multimodal analgesia being utilised more frequently in the non-neuraxial group, poorly controlled *dynamic* pain (more than 5 on numerical rating score) was most frequently found at all time points in this cohort (Fig. 1).

From postoperative day 3, both neuraxial analgesia groups had poorly controlled *dynamic* pain potentially attributable to the finite duration of action of intrathecal morphine or thoracic epidural analgesia failure at this stage. Given the disproportionate use of epidural analgesia in the more extensive surgery, higher *dynamic* pain scores at this stage might be reflective of slower recovery due to greater surgical insult. Improving the function of thoracic epidural analgesia is unlikely to be a solution, given consistent evidence describing functional issues with its use (Salicath et al. 2018). However, mechanisms to increase the duration of effect of intrathecal morphine via adjuncts such as alpha-2 agonists, e.g. clonidine, or combined spinal epidural techniques merit ongoing research.

Overall, the advantage of thoracic epidural analgesia over intrathecal morphine and non-neuraxial cohorts in reducing the probability of poorly controlled *rest* and *dynamic* pain was evident regardless of surgical approach and extent of surgery. The benefit over intrathecal morphine (4–8%) was smaller compared to non-neuraxial (2–18%) analgesia, but generally more consistent until postoperative day 3. Compared to non-neuraxial analgesia, the probability of poorly controlled pain on postoperative day 0, favoured intrathecal morphine across all subgroup analyses, again likely reflective of the pharmacodynamics of neuraxial delivered morphine. Data extending beyond 72 h following surgery was not collected; hence, the incidence and impact of rebound pain on pain scores or opioid consumption following cessation of thoracic epidural analgesia in those patients with functional epidurals is unknown.

It is noteworthy that no difference in the probability of poorly controlled *rest* or *dynamic* pain between thoracic epidural analgesia and non-neuraxial analgesia was determined from postoperative day 3 regardless of surgical extent. This likely relates to the lower-case complexity in the non-neuraxial group with faster recovery following surgery, however, an alignment with thoracic epidural analgesia failure commonly occurring on day 3 is another potential cause.

Pain scores and opioid consumption alone are inadequate surrogate markers for patient comfort and quality of recovery, which is ultimately what we are trying to optimise. Patient satisfaction or levels of distress related to pain or side effects need to be incorporated into future clinical trials for a more encompassing outcome assessment beyond simple numeric rating scales. These various effects include things as simple as pruritus and extend to a broader analysis of patient comfort in line with the Standardised Endpoints in Perioperative Medicine initiative (Myles et al. 2018), designed to evaluate analgesia related interventions including: pain intensity (at rest, during movement), postoperative nausea and vomiting, quality of recovery scale, time to gastrointestinal recovery, time to mobilisation, and sleep quality.

Respiratory depression and sedation are often regarded as barriers to the widespread use of intrathecal morphine, which occurred more frequently in this group compared to the other two comparator groups. Whilst no morbidity was correlated to these adverse events, the incidence is higher than that reported in a recent meta-analysis (Koning et al. 2020). The large dose of intraoperative opioid and intrathecal opioid administrated to this group may have contributed to this finding. More than 55% of patients across all analgesia groups experienced postoperative nausea and vomiting.

Overall, most patients were discharged home on an opioid. This surrogate metric for analgesic requirement is limited in its utility to accurately assess opioid need whereas opioid consumption in the 24 h preceding discharge, or requirement of prescription to refill opioid would be more informative. Unfortunately, longer-term follow-up data were not available to ascertain the incidence of persistent opioid use, shown to be as high as 10% in opioid naïve patients following surgery (Stark et al. 2017; Clarke et al. 2014).

The large number of patients included is a strength of this study. However, it is limited by the retrospective design, heterogenous group of upper gastrointestinal surgeries with variable surgical insult and recovery profiles, and inability to measure quality of recovery or patient comfort outcomes. Furthermore, confounding by indication was apparent, with the chosen analgesic technique heavily influenced by the procedure, surgical approach (open or laparoscopic) and institutional preference. The strength of predictions relies on the data points entered, with more uncertainty when there are fewer supporting data points.

## Conclusions

With a lens of identifying strategies to reduce opioid requirements and perioperative complications, our analysis of a large retrospective cohort observed potential positive impact with improved postoperative pain by integrating neuraxial techniques across varying major abdominal surgeries and operative approaches. Prospective, multicentre studies are required to provide further, evidenced-based, guidance on the appropriate management of these patients undergoing major surgery with direct comparisons of thoracic epidural analgesia and intrathecal morphine justified amongst patients undergoing similar surgical procedures. Measurement of patient comfort related parameters, methods to extend the duration of intrathecal morphine effect, and utilisation of alternative regional techniques within a multimodal analgesia strategy would also be informative.

## Supplementary Information

**Additional file 1.** a Post-operative morphine requirements. b.  Post-operative rest pain. c Post-operative dynamic pain.

## Data Availability

Datasets used and/or analysed during the current study are available from the corresponding author on reasonable request. Analysis of data during this study are included in this published article (and its supplementary information files).

## Abbreviations

ASA: American Society of Anesthesiologists
ERAS: Enhanced Recovery After Surgery
IQR: Interquartile range
NHST: Null hypothesis significance testing
Pr(Diff…): Probability of difference
