# Supplementary material for: Postoperative analgesia for upper gastrointestinal surgery: a retrospective cohort analysis
Source: Perioper Med (Lond). 2023 Jul 18;12:40. doi: 10.1186/s13741-023-00324-0 (PMC10355044; doi:10.1186/s13741-023-00324-0)
Supplement: Supplementary file 1 — Additional file 1. a Post-operative morphine requirements. b. Post-operative rest pain. c Post-operative dynamic pain. [file 13741_2023_324_MOESM1_ESM.zip › Additional file 1a.pdf]

Supplementary Material 1a

Code ▾

Post-operative Morphine Requirements

1 The Data

Here, we can see

1. Each individual's OMED requirement trajectory (thin lines), for the different analgesic technique groups over the post-operative days.
2. The group's median and IQR are also shown (thick lines).
3. The group's trend and distributions can be seen appreciated with the boxplots.
4. The density plots show more clearly the distributions of the OMED with the black lines showing the 25th, 50th and 75th quantiles.

It can be appreciated that the trends over the 4 days post-op aren't all linear.
It can also be appreciated that there are quite a lot of inter-individual variabilities within each group.
The density plot of OMED shows that the OMED requirements are highly skewed and its log OMED density shows there seems to be bimodality in its distribution.

NN: Non-neuraxial
SPI: Intrathecal Morphine
EPI: Thoracic Epidural Analgesia

Table 1

Group	Days	mean_omed	se_omed	q0_omed	q25_omed	q50_omed	q75_omed	q100_omed
NN	0	46.78	3.74	0	8.25	32.0	72.75	237
NN	1	94.52	8.09	0	23.00	66.0	129.00	636
NN	2	65.30	6.55	0	9.00	38.5	83.25	486
NN	3	46.28	5.48	0	0.00	25.5	60.00	426
SPI	0	25.89	4.53	0	0.00	12.0	31.50	237
SPI	1	115.89	14.94	0	27.00	72.0	155.00	771
SPI	2	117.19	14.41	0	33.50	65.0	196.50	648
SPI	3	84.76	10.51	0	12.75	55.0	126.75	366
EPI	0	7.99	1.78	0	0.00	0.0	0.00	189
EPI	1	29.06	6.70	0	0.00	0.0	10.00	856
EPI	2	32.28	6.51	0	0.00	0.0	17.50	750
EPI	3	38.51	6.83	0	0.00	0.0	30.00	747

Figure 1

Analgesic Requirements Post-op by Analgesic Groups
Data

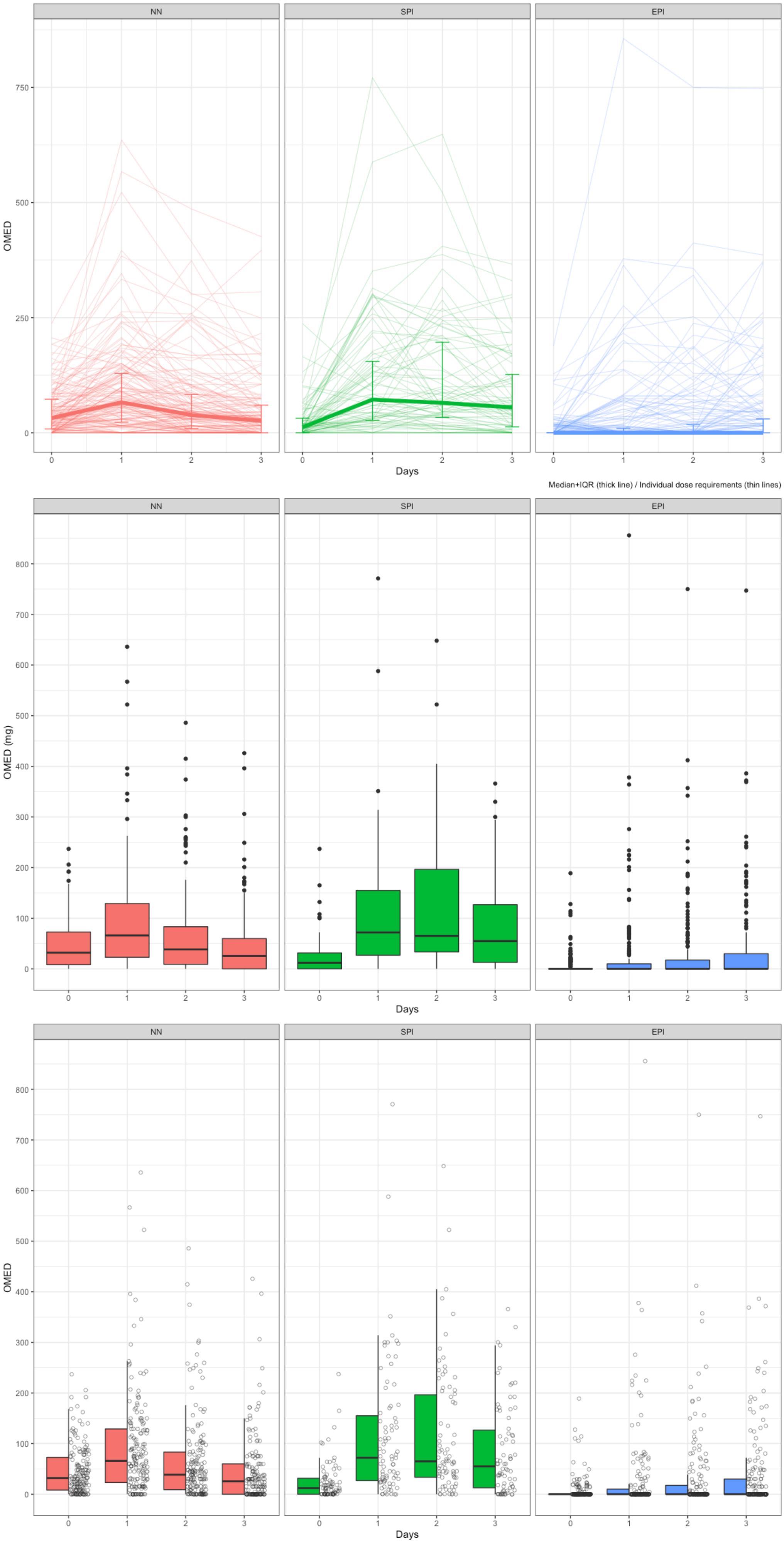

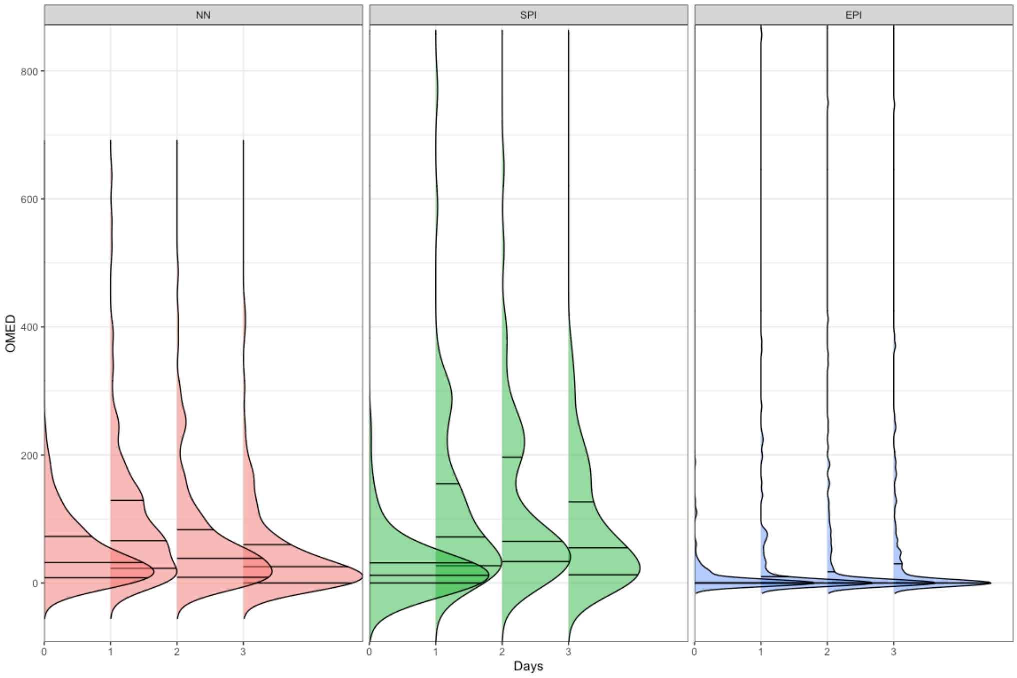

2 Goals of the analysis

We are interested in

1. OMED requirements with different analgesic techniques over the post-operative period
2. Difference in OMED requirements if one were to have an alternative analgesic technique
3. We want to see how they compared between different surgical approaches and surgical techniques

We consider a difference of >10mg is clinically significant.

3 The Model

The outcome variable OMED is highly skewed, as would be expected with medication use.
Taking the logarithm of (OMED+1), it can also be seen that there is bi-modality with an excess of zero OMED use.

Figure 2

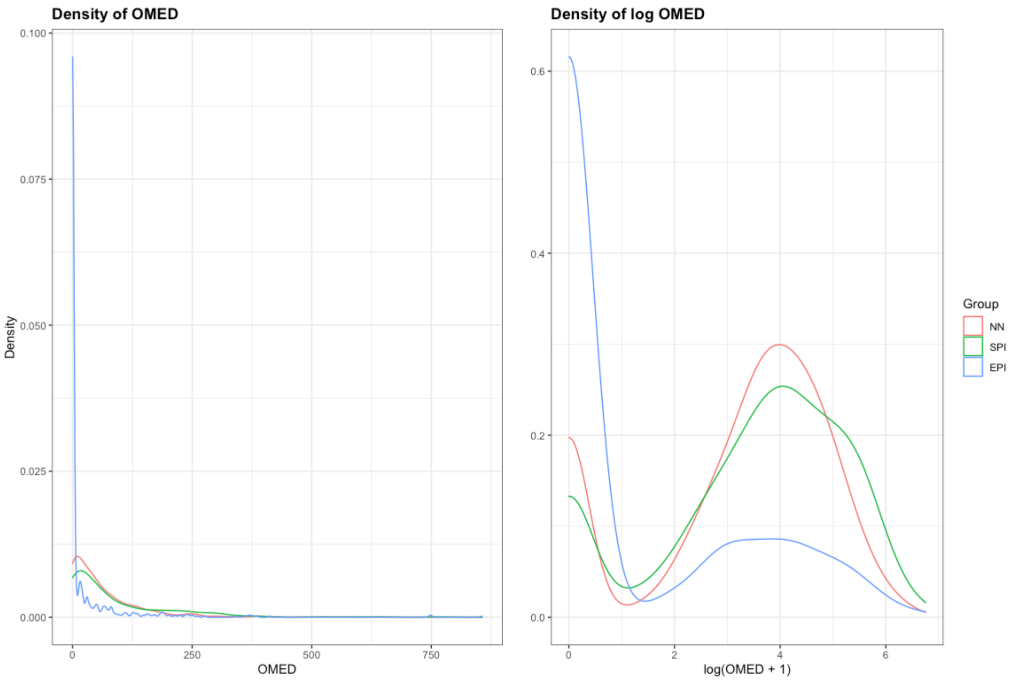

The next question is if there is any need for a multi-level structure.
If we first fit a linear model, with log(OMED+1) as y, and examine the within-patient residuals, we can see that there is considerable variations in the residuals corresponding to the same patient. They do not hover around 0, and many of them have the same sign within the same patient.

Figure 3

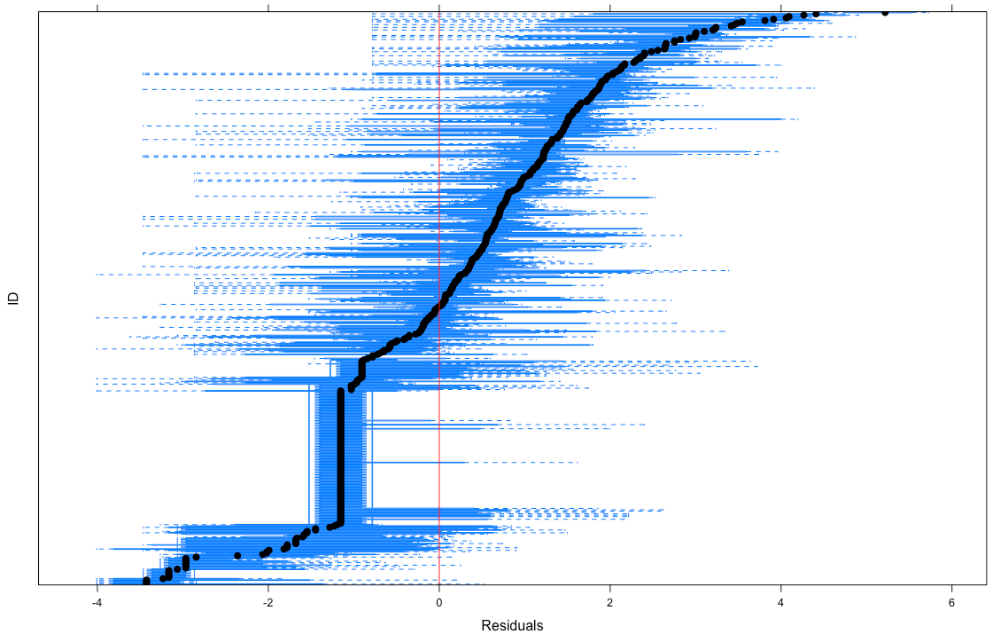

If we fit a linear regression for each patient separately, we can estimate each patient's own intercept and slope. These are shown in the blue circles. It can be seen that there is also considerable patient-to-patient variability in the intercepts. This is less so in the slopes but still present.

The pink circles are the individual intercept and slopes from fitting a linear mixed effect model. The individual estimates tend to be "pulled toward" the fixed-effects estimate (dotted line). This effect is perhpas more noticeable for the slopes.

This gives us some confidence about the random-effect structure to include in the model for our data.

Figure 4

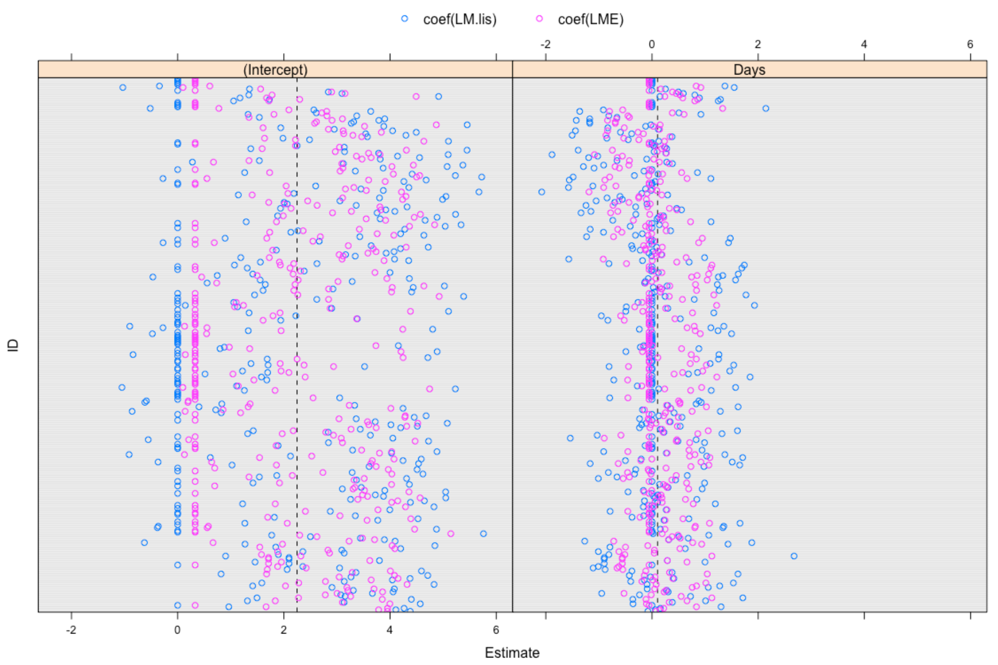

The data has a hierarchical structure, with the outcome variable OMED being highly skewed with an excess of zero. The data generating process for the zero is considered to be the same as the data generating process for non-zero values. They are both reflections of a patient's pain and extend of surgery.

We also noted that the OMED requirements for each individual trend over time aren't linear.

The adjusting variables have been chosen to be collected *a priori*. We consider two kinds of adjusting variables - intrinsic to the patient such as Age, Gender and their ASA classification, and extrinsic to the patient such as the surgical approach and the surgical type chosen. The model thus includes interaction terms for the "extrinsic" variables against time.

3.1 Model specifications

I have therefore chosen to use a generalised mixed effect modelling with a hurdle-lognormal link. The linearity assumption is also relaxed by using natural splines on the time ("Days") predictor. This allows within individual correlations to be accounted for the multiple measurements taken over time. The mixed effect included random effects on both the intercepts and slops at the individual level for the response over time. The hurdle part is modelled with logistic regression with the same mixed effect structure, predictors and interaction terms, but without the natural spline.

$$Pr(y|\pi,\theta) = \begin{cases} \pi, & y = 0 \\ (1-\pi)f(y|\theta), & y > 0 \end{cases}$$

Here $0 \leq \pi \leq 1$ is the probability that the response variable y is exactly equal to zero and $f(y|\theta)$ is the density function conditional on $y > 0$. Here, y represents the OMED, and we can specify $f(y|\theta) = f(y|\mu,\sigma)$ where μ and σ are its location and scale parameters respectively. They are also the mean and variance of the logarithm of the variable, which by definition, is normally distributed.

The hurdle part is specified with a binomial distribution, where

$$y_i \sim Binomial(n, \pi_i)$$
$$logit(\pi_i) = \alpha_{ID[i]} + \beta_{ID[i]}$$

Likewise, the $LogNormal$ likelihood is specified as,

$$log(y_i) \sim Normal(\mu_i, \sigma_i)$$
$$\mu_i = \alpha_{ID[i]} + \beta_{ID[i]} + ns(\beta^{Time}_{ID[i]})$$
$$\alpha_j \sim Normal(\overline{\alpha}, \sigma_\alpha)$$
$$\beta_j \sim Normal(\overline{\beta}, \sigma_\beta)$$

Note $ns()$ is the natural spline function (from spline package) applied to the "time" component of the model.

For this analysis, we will adapt the Bayesian approach. It is useful in this application as it is flexible for specifying exactly the desired model structure. The brms package in R and Stan software can provide the necessary computation. The Bayesian approach also directly yields credible intervals and posterior predicted responses for direct comparisons of OMED requirements that we are interested in.

3.2 Priors

We will use previous studies (*PrvStyPr*) to inform our prior choice for the initial average analgesic requirement without the effects of the adjusting variables. We will also set the prior for the effects of the adjusting variables roughly similar in multitude to previous studies. We will set the priors for the scales parameters such that it can generate plausible response values within the 99% credible interval but still broad enough to accommodate extreme values.

Two other priors will be utilised to see their effects on the posterior as compared to our model prior.
Broad priors (*BrPr*) will encompass very broad responses, even highly extreme values, such that they should have limited influence on the results and that the data, via the likelihood function, should dominate the posteriors. We will also use sceptical priors (*ScptPr*) which favours results sceptical of large effects.

3.2.1 *PrvStyPr*

Previous studies (references from KP) have shown the following mean morphine requirements after abdominal surgeries:

```
- Major abdo 24hours: 27.5+/- 4.3mg
- Colorectal cumulative to 72hours: 62+/-26mg vs 31+/-17mg; 24 hours: 20mg vs 10mg
- Major abdo 24hours: 40+/-26 vs 9+/-17mg
- Hepatic at 48hours: 124+/-30mg vs 47+/-21mg; 24hours: 62mg vs 25mg
- Hepatic at 24 hours: 50mg
```

So the average requirement for major abdominal surgery is around 27.5mg at 24 hours, and in some studies can go up to an average of 60mg. At 48hr, it can go up to 124mg, roughly 2x as much as at 24hr.

We also know from experience that the maximum OMED requirement, even for those who are opioid-tolerant, is very unlikely to be more than 2000mg.

We will utilise this information to set the prior for α (Intercept) $\sim N(3.3, 0.4)$, which corresponds to an average baseline dose without effects of any adjusting variables with 95% probability between roughly 10 to 60mg.

For β (adjusting variable, $\sim N(0, 0.46)$), we will set its multiplicative effect to $\frac{1}{2.5}$ to 2.5-fold with 95% probability. We will also make the estimates more precise by setting a smaller prior for its scale parameter $\overline{\sigma}_\beta$.

The scales parameters/hyperparameters are set

- $\sigma \sim (0, 1)$, such that roughly the maximum morphine requirement will be less than 2000mg, within the 99% credible limits.
- $\overline{\sigma}_\alpha \sim N(0, 1)$, such that the group-level random intercept variability is broad enough to include all plausible values.
- $\overline{\sigma}_\beta \sim N(0, 0.35)$, such that the group-level random slope variability is precise enough to the unwanted side effect of extreme exponential growth with increasing Days in the response with a lognormal distribution.

$$
\begin{aligned}
log(y_i) &\sim Normal(\mu_i, \sigma) \\
\mu_i &= \alpha_{ID[i]} + \beta_{ID[i]} + ns(\beta^{Time}_{ID[i]}) \\
\alpha_j &\sim Normal(\overline{\alpha}, \sigma_\alpha) \\
\beta_j &\sim Normal(\overline{\beta}, \sigma_\beta) \\
\beta^{Time}_j &\sim Normal(\overline{\beta}, \sigma_\beta) \\
\overline{\alpha} &\sim Normal(3.3, 0.4) \\
\overline{\beta} &\sim Normal(0, 0.46) \\
\overline{\sigma}_\alpha &\sim Normal(0, 1) \\
\overline{\sigma}_\beta &\sim Normal(0, 0.35) \\
\sigma &\sim Normal(0, 1)
\end{aligned}
$$

3.2.1.1 Prior predictive check

Here the error bars represent 95% credible limits. The dotted error bars represent 99% credible limits for comparison. The black circles are our sample response. Most of our data lie within the 95% credible interval with some outliers. But all of our data lie within the 99% credible interval.

Figure 5

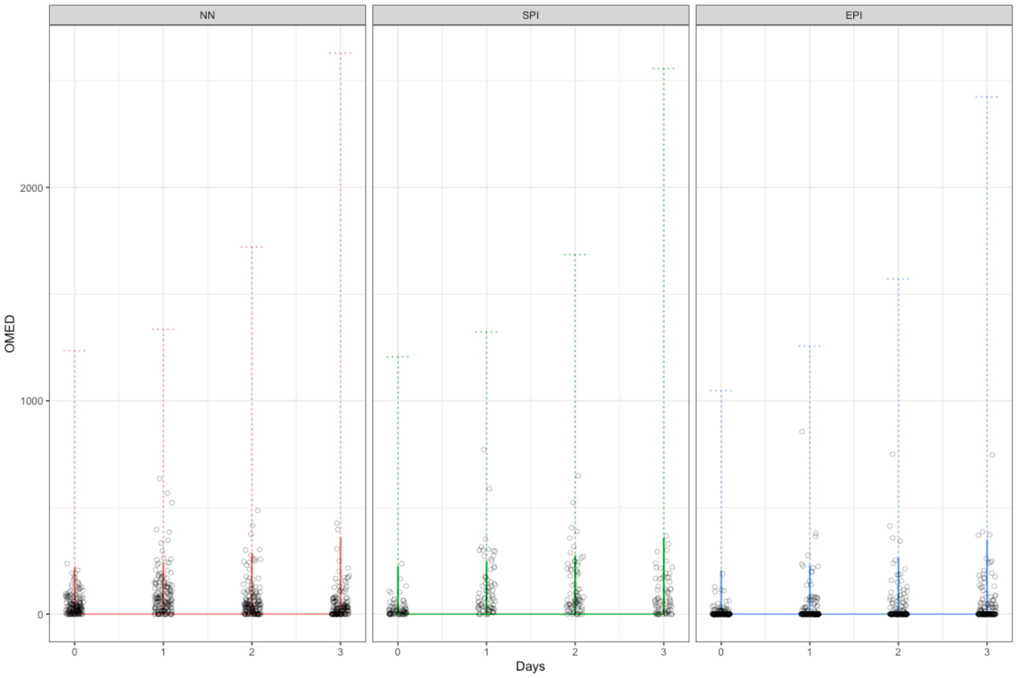

3.2.1.2 Posterior predictive check

The model's posterior predictions are plotted below, superimposed on the data.
The data are shown by the circles in gray. The dotted gray lines are the median of the data.
The posterior predicted medians are shown by the circle and the trend represented by the adjoining line. The colour represents different analgesic groups. The error bars show their 95% credible intervals.

It can be seen from the plot that the model describes the data very well.

Figure 6

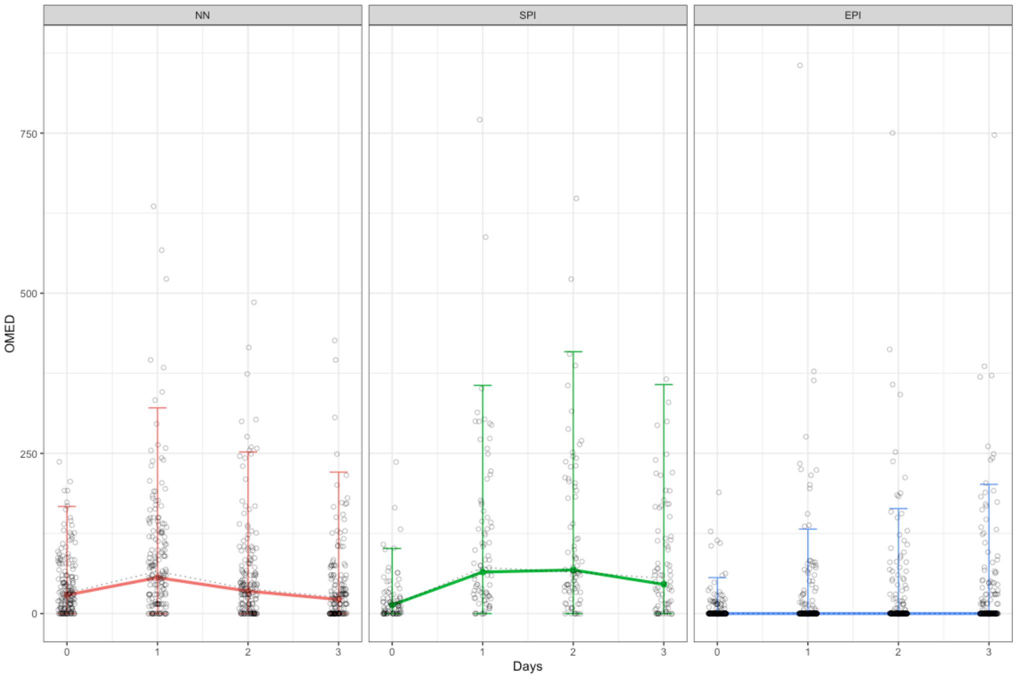

3.3 Priors for sensitivity analyses

Two other priors will be generated to see their effects on the posterior as compared to our model prior. Broad priors (*BrPr*) will encompass very broad responses, even highly extreme values such that they should have limited influence on the results and that the data, via the likelihood function, should dominate the posteriors. We will also use sceptical priors (*ScptPr*) which favours results sceptical of large effects.

3.3.1 *BrPr*

We know from experience, that although possible for some minority of patients, the average daily morphine is unlikely to be more than 800mg (if a person uses a morphine PCA with 1mg bolus and 5min lock-out, and he/she uses it every 5min for 24 hours for argument's sake, he/she would have pressed 12 boluses per hour for 24 hours = 288mg iv morphine = 864mg OMED).

The following priors will be used:

- **Intercept** / $\alpha \sim N(4.6, 1)$: A normally distributed prior with $\mu = 4.6$ and $\sigma = 1$ will be used. With log-normal distribution of the response variable y, y is in a different scale to μ and σ. After exponentiating, this prior corresponds to an average baseline dose with 95% probability between roughly between 10mg and 750mg. Taking into account within individual standard deviation, without adding the effect of the adjustment variables, the initial morphine requirement can be as high as 2000mg 99% of the time.
- **Adjustment variables** / $\beta \sim N(0, 0.8)$: Effects of βs are multiplicative. Here we will set the prior such that *each* adjustment variable has an exponential growth (or decay effect depending on the sign) of 5-fold (or $\frac{1}{5}$ -fold), 95% of the time.
- We will use the same distributions for the scales parameters.

$$
\begin{aligned}
log(y_i) &\sim Normal(\mu_i, \sigma) \\
\mu_i &= \alpha_{ID[i]} + \beta_{ID[i]} + ns(\beta^{Time}_{ID[i]}) \\
\alpha_j &\sim Normal(\overline{\alpha}, \sigma_\alpha) \\
\beta_j &\sim Normal(\overline{\beta}, \sigma_\beta) \\
\beta^{Time}_j &\sim Normal(\overline{\beta}, \sigma_\beta) \\
\overline{\alpha} &\sim Normal(4.6, 1) \\
\overline{\beta} &\sim Normal(0, 0.8) \\
\overline{\sigma}_\alpha &\sim Normal(0, 1) \\
\overline{\sigma}_\beta &\sim Normal(0, 0.35) \\
\sigma &\sim Normal(0, 1)
\end{aligned}
$$

3.3.1.1 Prior predictive check

The broad prior was specified abstractly with some sensible values for the intercept to provide opportunity for extreme responses. Here the error bars represent 95% credible limits. The black circles are our sample response which confirms that they are within plausible values (within 95% credible limits) of the broad prior. Note 99% credible interval does extends to include very broad values including those that are extremely large that can be unrealistic and out of scale.

Figure 7

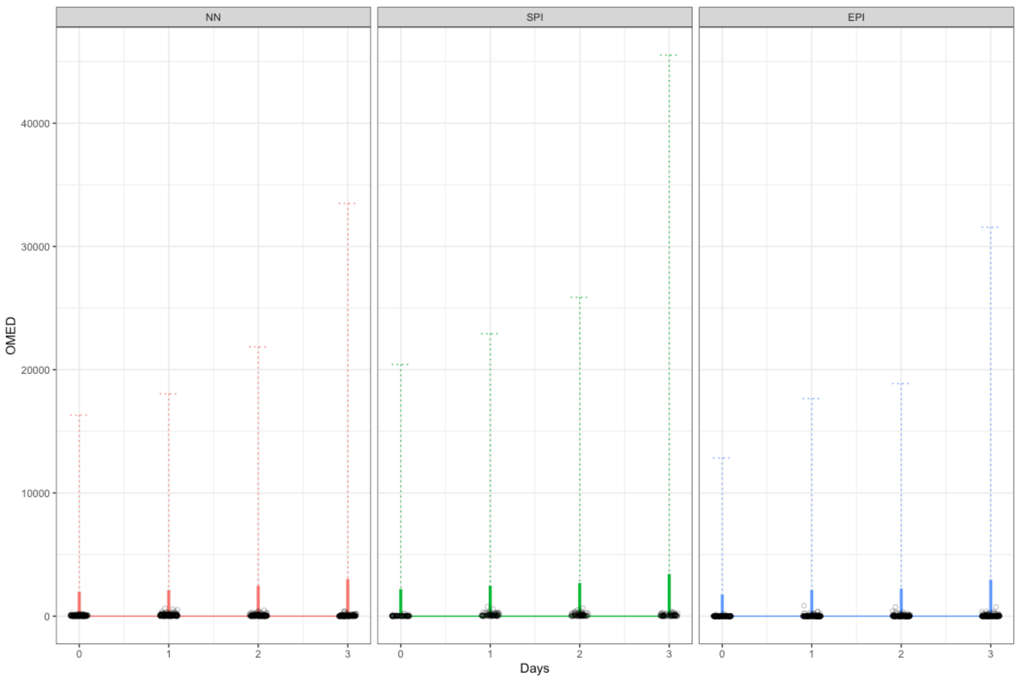

3.3.1.2 Posterior predictive check

It can be seen from the plot that the posteriors reasonably describes the data.

Figure 8

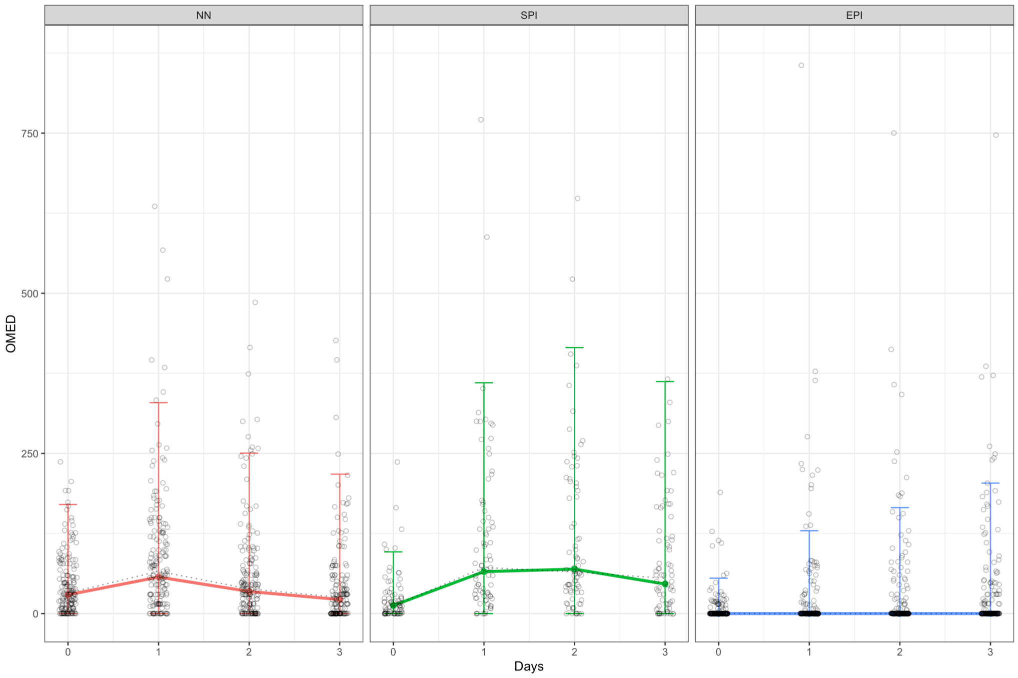

3.3.2 *ScptPr*

Sceptical priors are sceptical of large effects.

Here, we will use the same prior for the initial average morphine requirements α (intercept), but limit the Group-level random effects. We will also limit the effects of adjusting variables (β) to be very precise and symmetrical around 0.

$$
\begin{aligned}
log(y_i) &\sim Normal(\mu_i, \sigma) \\
\mu_i &= \alpha_{ID[i]} + \beta_{ID[i]} + ns(\beta^{Time}_{ID[i]}) \\
\alpha_j &\sim Normal(\overline{\alpha}, \sigma_\alpha) \\
\beta_j &\sim Normal(\overline{\beta}, \sigma_\beta) \\
\beta^{Time}_j &\sim Normal(\overline{\beta}, \sigma_\beta) \\
\overline{\alpha} &\sim Normal(3.3, 0.4) \\
\overline{\beta} &\sim Normal(0, 0.01) \\
\overline{\sigma}_\alpha &\sim Normal(0, 0.1) \\
\overline{\sigma}_\beta &\sim Normal(0, 0.01) \\
\sigma &\sim Normal(0, 1)
\end{aligned}
$$

3.3.2.1 Prior predictive check

Again the error bars represent 95% credible limits. The dotted error bars represent 99% credible limits for comparison. The black circles are our sample response.

The sceptical priors generates much more conservative responses.

Figure 9

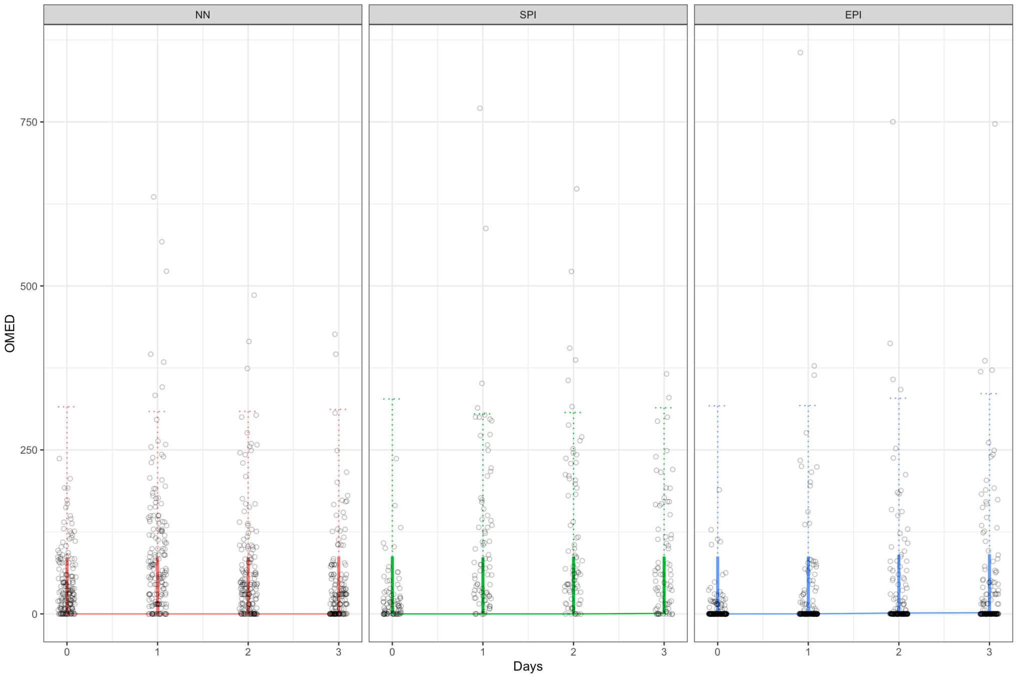

3.3.2.2 Posterior predictive check

It can be seen from the plot that the posteriors describes the data very well.

Figure 10

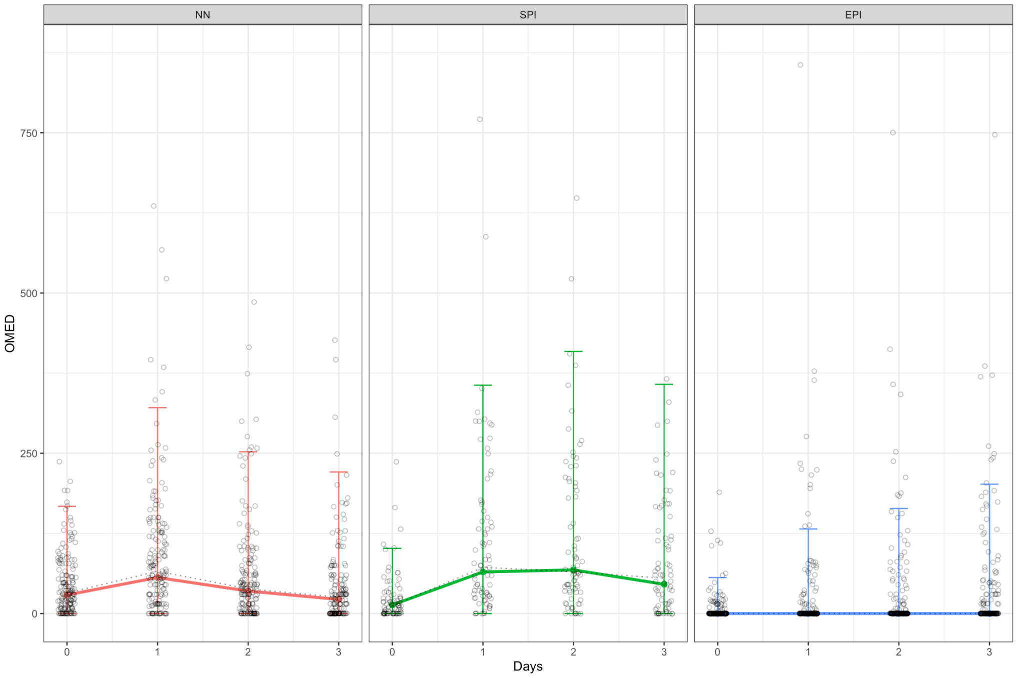

3.3.3 Checking sensitivity of posterior to choices of prior

3.3.3.1 Comparing posterior predictive responses

Different priors are represented by the different linetypes, superimposed on the same graph.
It can be seen that there is little, if any, influence of different priors on the posterior predictions.

Figure 11

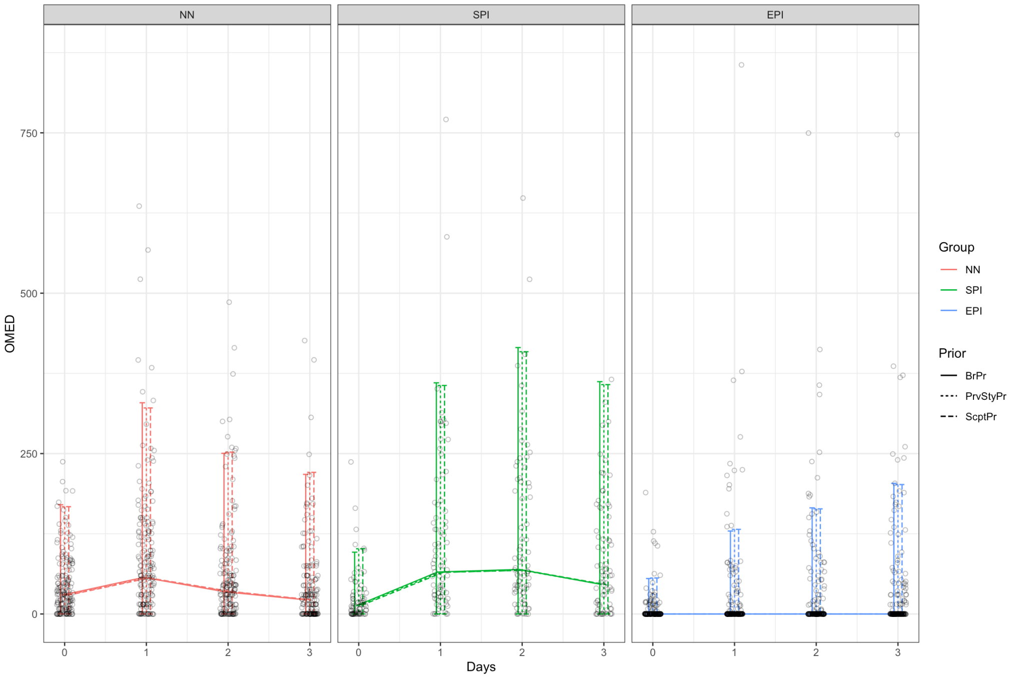

3.3.3.2 Comparing posterior predictions if one were to have a different analegesic technique

See below under ***"4.2 Comparisons of differences in OMED requirements"***.
In short, this is to compare the differences in OMED requirements if one were to change the original analgesic technique and to have received a different analgesic technique.

The black dots are the medians of the posterior predicted differences. The error bars are the 95% credible intervals of the posterior predicted differences. The different priors are represented by different line types.

It can be seen that the different priors have little effects on the posterior predicted differences.

Figure 12

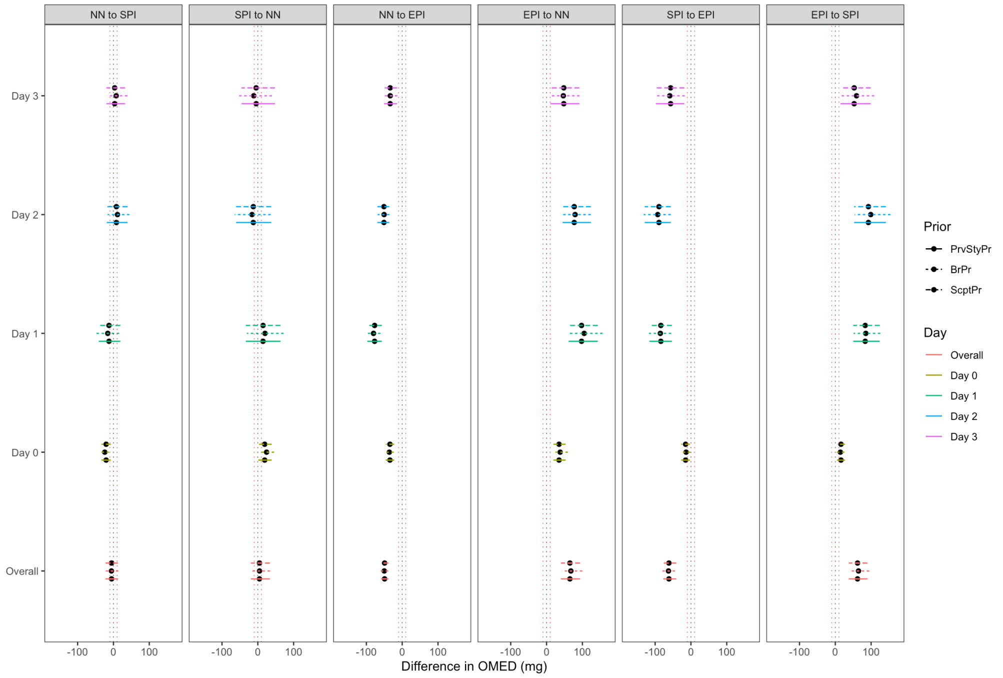

4 Adjusted OMED requirements

4.1 Comparisons of adjusted posterior OMED requirements

4.1.1 Median OMED between analgesic groups, and between Surgical Approaches

The plot shows the post-op median OMED requirements for the different analgesic groups, for a typical patient who is

```
- 60 years of age
- Female
- ASA = 3
- Surgical type = Type 1
```

Figure 13

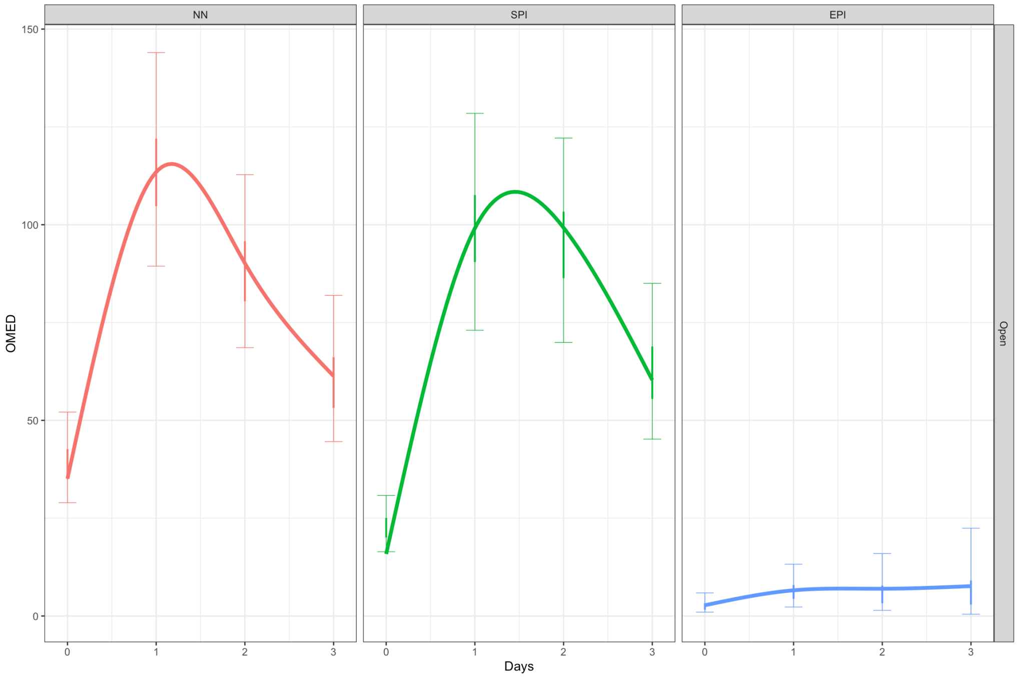

Table 2

Group	Days	SurgAppr	Median	CI95_high	CI95_low
NN	0	Open	40.16	52.11	28.96
NN	1	Open	115.90	144.00	89.41
NN	2	Open	88.79	112.81	68.59
NN	3	Open	62.56	81.93	44.58
SPI	0	Open	23.03	30.82	16.44
SPI	1	Open	101.00	128.46	73.03
SPI	2	Open	97.17	122.15	69.91
SPI	3	Open	63.38	85.01	45.21
EPI	0	Open	2.96	5.91	0.98

EPI	1	Open	6.64	13.23	2.31
EPI	2	Open	6.87	15.97	1.45
EPI	3	Open	7.72	22.44	0.47

Figure 14

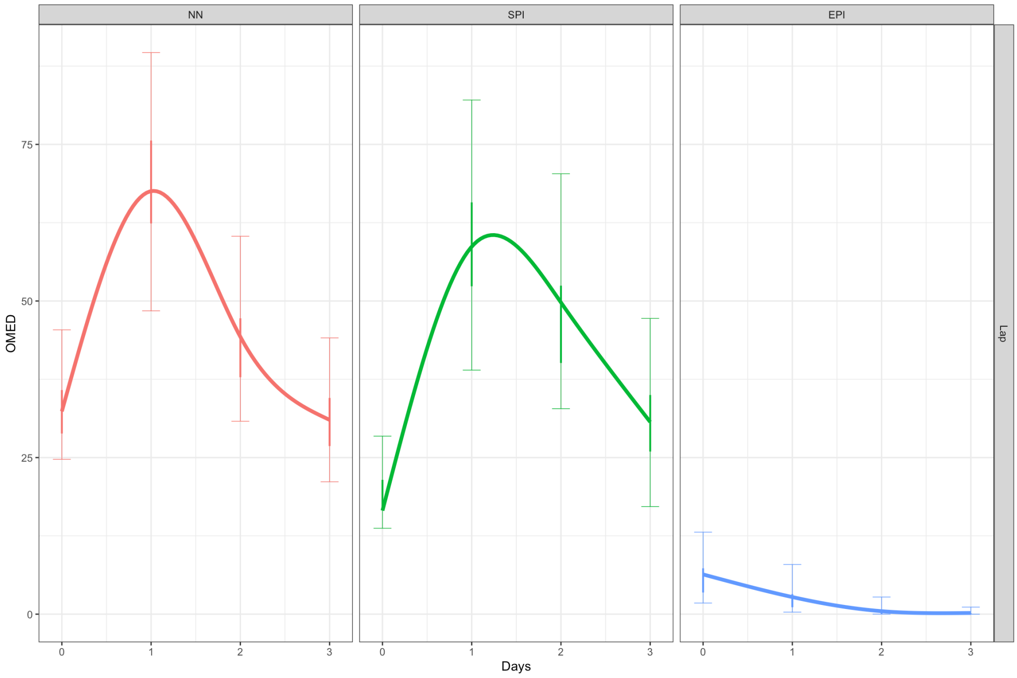

Table 3

Group	Days	SurgAppr	Median	CI95_high	CI95_low
NN	0	Lap	34.00	45.39	24.72
NN	1	Lap	68.73	89.66	48.42
NN	2	Lap	43.74	60.33	30.80
NN	3	Lap	30.91	44.10	21.14
SPI	0	Lap	19.80	28.42	13.71
SPI	1	Lap	59.67	82.09	38.97
SPI	2	Lap	48.68	70.31	32.80
SPI	3	Lap	31.78	47.22	17.17
EPI	0	Lap	6.19	13.10	1.78
EPI	1	Lap	2.75	7.94	0.32
EPI	2	Lap	0.51	2.73	0.01
EPI	3	Lap	0.12	1.13	0.00

4.1.2 Median OMED between analgesic groups, and between Surgical Types

The plot shows the post-op median OMED requirements for the different analgesic groups, for a typical patient who is

```
- 60 years of age
- Female
- ASA = 3
- Surgical Approach = Open
```

Figure 15

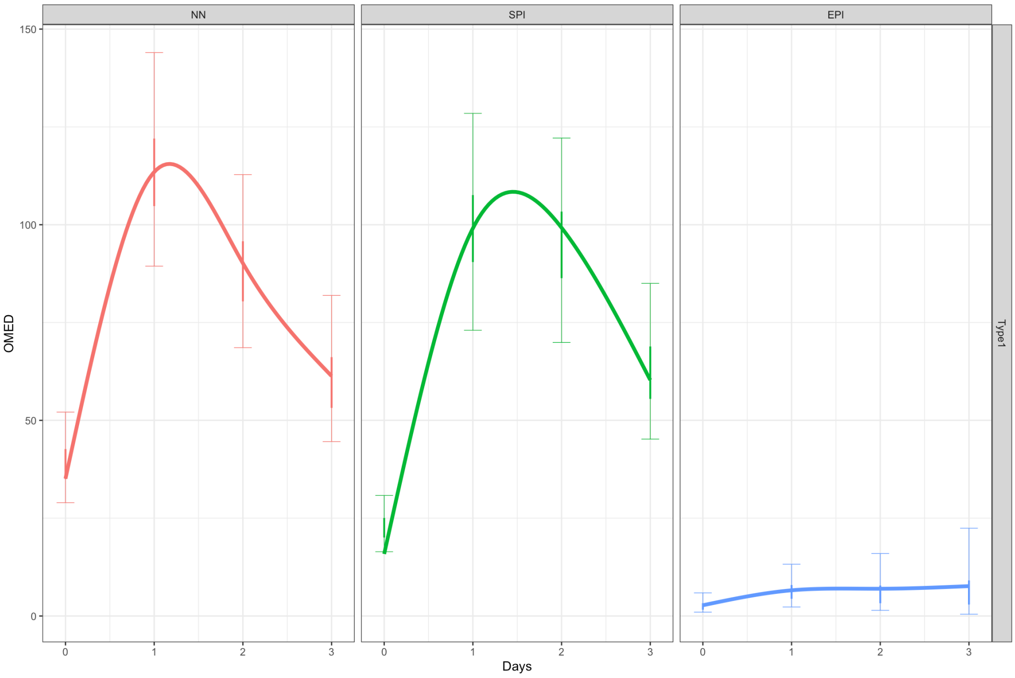

Table 4

Group	Days	SurgType	Median	CI95_high	CI95_low
NN	0	Type1	40.16	52.11	28.96
NN	1	Type1	115.90	144.00	89.41
NN	2	Type1	88.79	112.81	68.59
NN	3	Type1	62.56	81.93	44.58
SPI	0	Type1	23.03	30.82	16.44
SPI	1	Type1	101.00	128.46	73.03
SPI	2	Type1	97.17	122.15	69.91
SPI	3	Type1	63.38	85.01	45.21
EPI	0	Type1	2.96	5.91	0.98
EPI	1	Type1	6.64	13.23	2.31
EPI	2	Type1	6.87	15.97	1.45
EPI	3	Type1	7.72	22.44	0.47

Figure 16

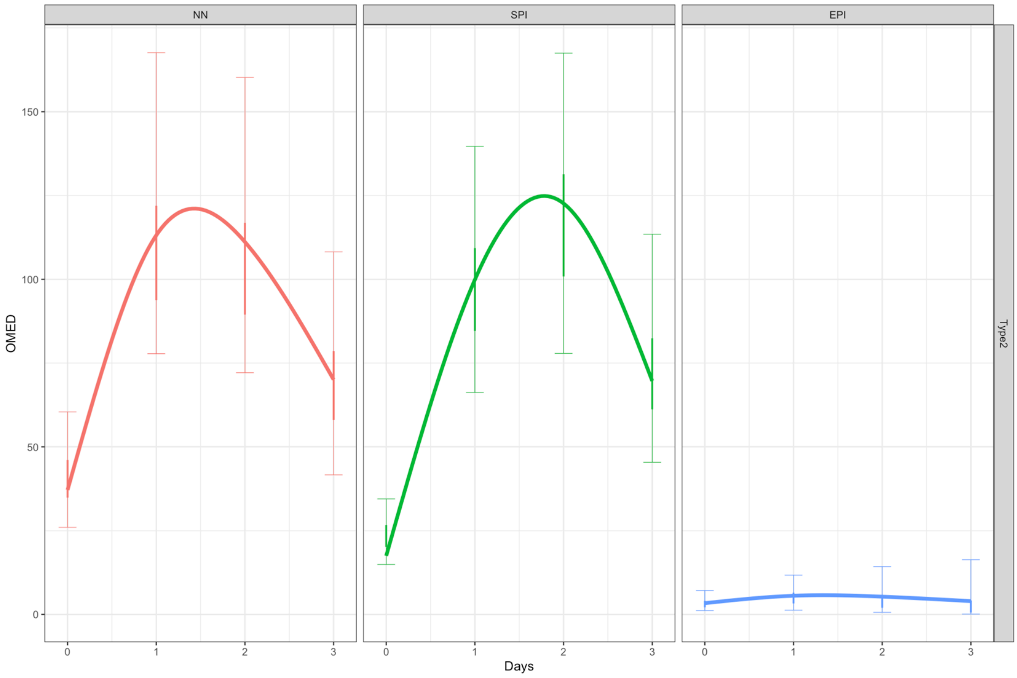

Table 5

Group	Days	SurgType	Median	CI95_high	CI95_low
NN	0	Type2	41.96	60.43	25.99
NN	1	Type2	114.50	167.62	77.80
NN	2	Type2	110.12	160.20	72.12
NN	3	Type2	72.33	108.19	41.64
SPI	0	Type2	23.99	34.46	14.90
SPI	1	Type2	101.10	139.63	66.24
SPI	2	Type2	121.61	167.46	77.88
SPI	3	Type2	73.68	113.44	45.39
EPI	0	Type2	3.44	7.13	1.18
EPI	1	Type2	5.58	11.73	1.27
EPI	2	Type2	5.27	14.27	0.62
EPI	3	Type2	4.02	16.31	0.10

Figure 17

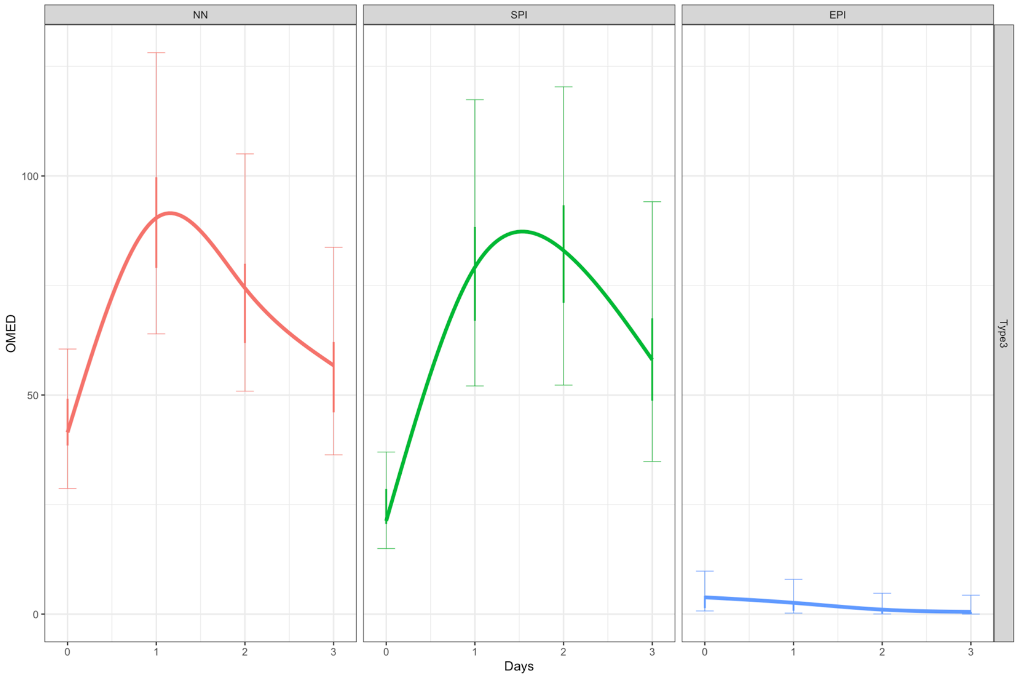

Table 6

Group	Days	SurgType	Median	CI95_high	CI95_low
NN	0	Type3	43.91	60.50	28.67
NN	1	Type3	91.58	128.14	63.95
NN	2	Type3	73.77	105.04	50.87
NN	3	Type3	57.57	83.71	36.35
SPI	0	Type3	25.40	36.98	14.95
SPI	1	Type3	80.37	117.39	52.07
SPI	2	Type3	82.02	120.35	52.25
SPI	3	Type3	59.92	94.13	34.82
EPI	0	Type3	3.75	9.80	0.72
EPI	1	Type3	2.56	7.93	0.23
EPI	2	Type3	1.04	4.74	0.02
EPI	3	Type3	0.48	4.30	0.00

4.2 Comparisons of differences in OMED requirements if one were to have a different analgesic technique

This is looking at for those who received a particular analgesic technique, the OMED difference if they were to receive a different analgesic technique (i.e. the difference in OMED if they otherwise received a different analgesic technique).

The plot shows the posterior predictive distributions of the differences - overall (over all 4 time points), and on separate days. Instead of controlling for the predictors and showing the result for a "typical patient", here I've taken all the characteristics from the patient sample who, say initially received "NN" and computed the posterior predictive distributions, and using the same characteristics, changed the analgesic technique to say "SPI", and taken the posterior predictive distribution. The difference in OMED is calculated by the posterior predictive OMED distributions for NN minus the posterior predictive OMED distributions if they were to receive SPI. This median dose difference distribution and its 95% (thin bar) and 50% (thick bar) credible intervals are shown as "NN to SPI". The same is done for "SPI to NN" and so on. The black dot is the median of the distribution.

The OMED difference is the analgesic requirement for the original technique minus the hypothetical analgesic technique one were to receive. For example, for "NN to SPI", it's the original OMED requirement if one had "NN" minus if one were to have "SPI".

The vertical gray line lines up the point when the difference equals zero. The red dotted lines are showing a difference of 10mg more or a difference of 10mg less, when one were to have a different analgesic technique.

Note that posterior predictive differences for “NN-SPI” and “SPI-NN” will not be just the reverse of the sign, because the baseline characteristics and other covariates of those that got NN in the audit may differ from the baseline characteristics and covariates of those who received SPI. This is becauase individuals are being modelled as one of the grouping levels in the mixed effect modelling, and this way it should reflect more realistically the proportion of the other characteristics (ASA, age, gender… etc) of those receiving, say “NN”, which may not be exactly the same as the characteristics of those who received “SPI”.

The overall differences stratified by Days have been shown above under **3.3.3.2** when checking sensitivities to the priors. Here we will show the posterior differences stratified by Surgical Approach and Surgical Types.

4.2.1 by Surgical Approaches

Figure 18

Oral morphine equivalent dose difference by surgical approach.
Black dot, median; thin bar, 50% credible interval; thick bar 95% credible interval; thin vertical line, zero difference; thin vertical line on either side, 10 mg difference.
Position on either side of central vertical line denotes whether more (right), or less (left) opioid is predicted.

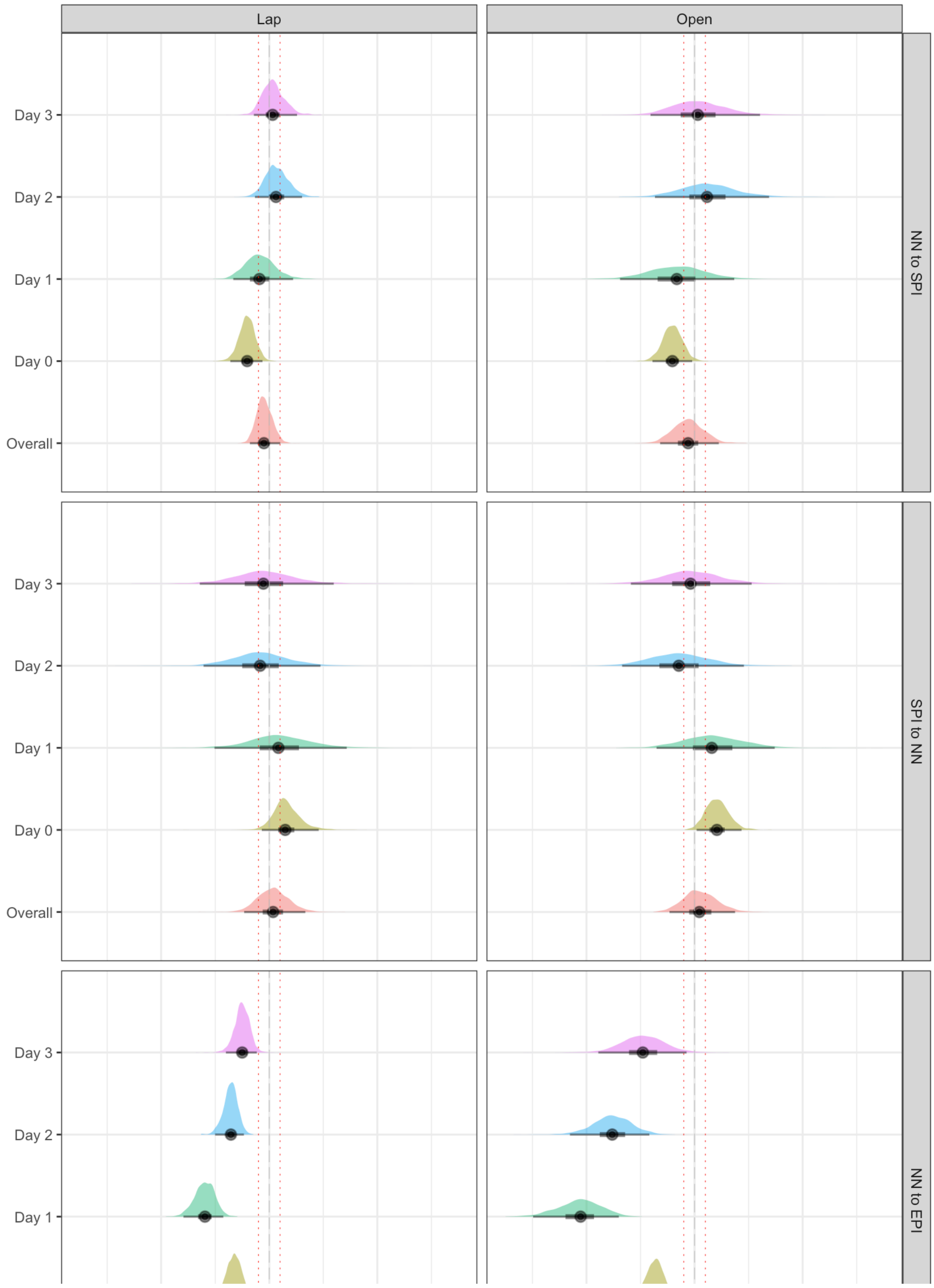

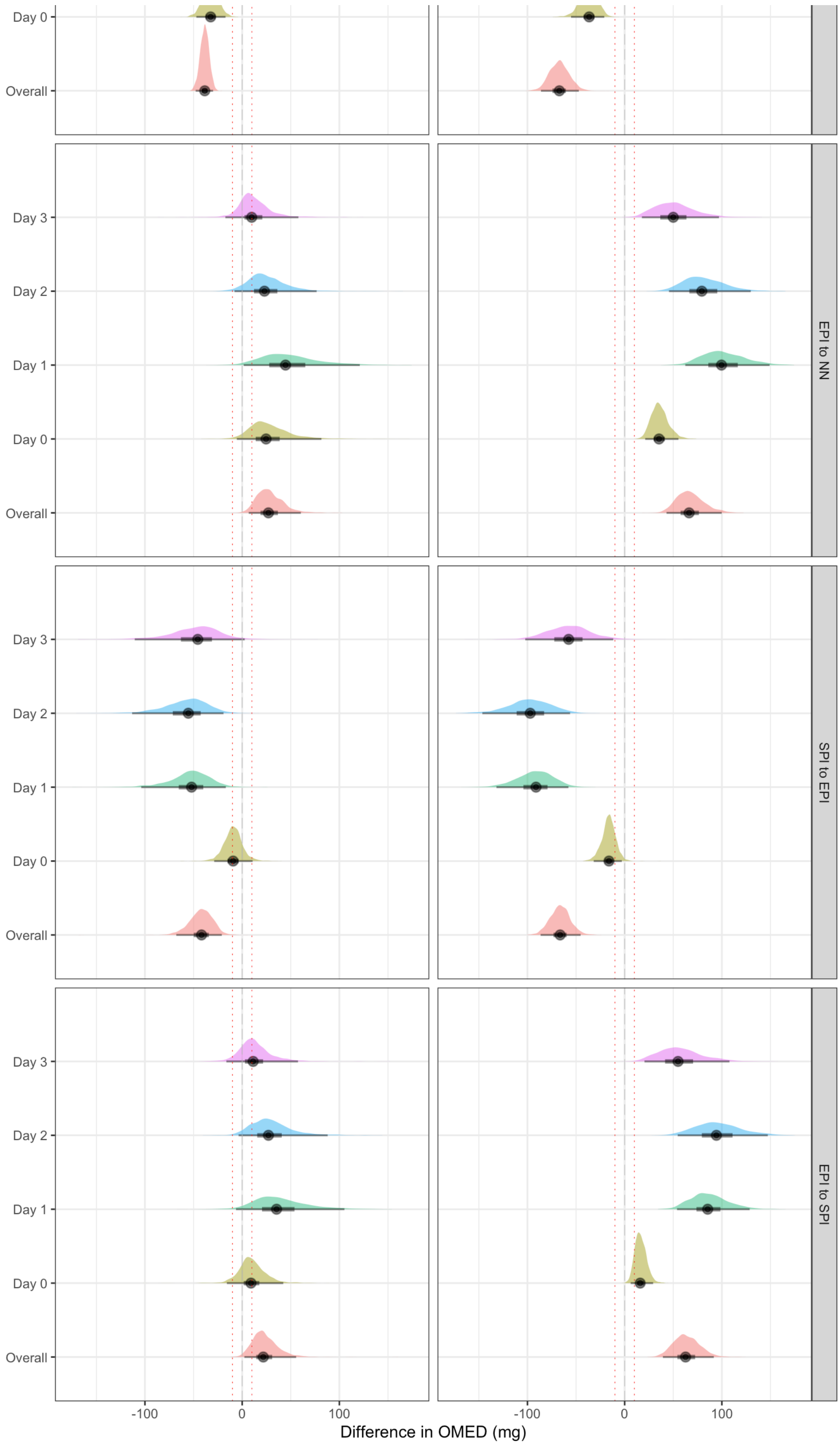

Table 7

SurgAppr	Diff	Day	Median	CI95_low	CI95_high	Pr(|Diff|>=10mg)	Pr(Diff>=10mg)	Pr(Diff<=-10mg)	Pr(-10mg
Lap	NN to SPI	Overall	-4.95	-18.33	9.34	0.25	0.03	0.22	
Lap	NN to SPI	Day 0	-20.43	-35.26	-5.88	0.92	0.00	0.92	

Lap	NN to SPI	Day 1	-9.09	-34.61	19.74	0.56	0.09	0.47
Lap	NN to SPI	Day 2	6.24	-15.31	28.02	0.42	0.38	0.05
Lap	NN to SPI	Day 3	3.10	-13.86	25.96	0.32	0.24	0.08
Lap	SPI to NN	Overall	3.54	-22.77	33.78	0.48	0.31	0.17
Lap	SPI to NN	Day 0	14.79	-9.45	42.57	0.70	0.69	0.01
Lap	SPI to NN	Day 1	8.45	-53.29	67.81	0.71	0.48	0.23
Lap	SPI to NN	Day 2	-8.51	-61.14	46.65	0.72	0.24	0.48
Lap	SPI to NN	Day 3	-5.51	-65.69	56.74	0.71	0.28	0.43
Lap	NN to EPI	Overall	-38.48	-47.21	-29.42	1.00	0.00	1.00
Lap	NN to EPI	Day 0	-32.43	-47.88	-18.13	1.00	0.00	1.00
Lap	NN to EPI	Day 1	-59.50	-80.22	-43.64	1.00	0.00	1.00
Lap	NN to EPI	Day 2	-35.48	-50.57	-23.92	1.00	0.00	1.00
Lap	NN to EPI	Day 3	-25.04	-41.65	-13.34	0.99	0.00	0.99
Lap	EPI to NN	Overall	27.05	5.76	58.57	0.94	0.94	0.00
Lap	EPI to NN	Day 0	24.61	-8.64	74.18	0.84	0.83	0.01
Lap	EPI to NN	Day 1	44.94	-0.51	120.11	0.94	0.93	0.01
Lap	EPI to NN	Day 2	22.95	-14.07	66.42	0.82	0.80	0.02
Lap	EPI to NN	Day 3	9.82	-20.76	52.07	0.55	0.50	0.05
Lap	SPI to EPI	Overall	-41.80	-66.16	-20.09	1.00	0.00	1.00
Lap	SPI to EPI	Day 0	-9.31	-28.82	10.76	0.50	0.03	0.47
Lap	SPI to EPI	Day 1	-52.07	-101.73	-15.52	0.99	0.00	0.99

Lap	SPI to EPI	Day 2	-55.37	-106.42	-15.34	0.99	0.00	0.99
Lap	SPI to EPI	Day 3	-45.72	-110.73	3.93	0.96	0.02	0.94
Lap	EPI to SPI	Overall	21.72	0.35	50.44	0.87	0.87	0.00
Lap	EPI to SPI	Day 0	9.08	-16.91	39.38	0.52	0.46	0.06
Lap	EPI to SPI	Day 1	35.52	-8.32	106.22	0.91	0.89	0.02
Lap	EPI to SPI	Day 2	27.09	-9.31	77.24	0.85	0.84	0.01
Lap	EPI to SPI	Day 3	11.29	-18.13	53.75	0.60	0.54	0.05
Open	NN to SPI	Overall	-5.95	-31.90	22.42	0.52	0.14	0.38
Open	NN to SPI	Day 0	-20.53	-40.01	-3.63	0.88	0.00	0.87
Open	NN to SPI	Day 1	-16.42	-67.92	37.13	0.75	0.16	0.59
Open	NN to SPI	Day 2	11.50	-38.82	66.24	0.71	0.52	0.19
Open	NN to SPI	Day 3	2.98	-44.66	54.14	0.67	0.38	0.29
Open	SPI to NN	Overall	4.41	-23.24	37.48	0.53	0.37	0.16
Open	SPI to NN	Day 0	20.70	1.60	42.77	0.85	0.85	0.00
Open	SPI to NN	Day 1	15.91	-39.01	70.17	0.76	0.60	0.16
Open	SPI to NN	Day 2	-14.57	-69.09	42.53	0.76	0.19	0.57
Open	SPI to NN	Day 3	-3.78	-56.24	54.09	0.71	0.31	0.40
Open	NN to EPI	Overall	-67.20	-86.27	-47.23	1.00	0.00	1.00
Open	NN to EPI	Day 0	-36.52	-53.51	-19.66	1.00	0.00	1.00
Open	NN to EPI	Day 1	-105.46	-145.04	-67.40	1.00	0.00	1.00
Open	NN to	Day 2	-76.18	-109.64	-38.61	1.00	0.00	1.00

	EPI							
Open	NN to EPI	Day 3	-47.86	-84.61	-5.05	0.97	0.00	0.97
Open	EPI to NN	Overall	66.30	41.46	97.26	1.00	1.00	0.00
Open	EPI to NN	Day 0	35.35	20.88	54.30	1.00	1.00	0.00
Open	EPI to NN	Day 1	99.69	58.70	145.16	1.00	1.00	0.00
Open	EPI to NN	Day 2	79.37	42.85	126.38	1.00	1.00	0.00
Open	EPI to NN	Day 3	49.98	16.14	94.74	0.99	0.99	0.00
Open	SPI to EPI	Overall	-66.38	-87.92	-46.95	1.00	0.00	1.00
Open	SPI to EPI	Day 0	-16.13	-31.27	-2.62	0.83	0.00	0.83
Open	SPI to EPI	Day 1	-91.25	-131.04	-56.93	1.00	0.00	1.00
Open	SPI to EPI	Day 2	-97.25	-143.08	-54.13	1.00	0.00	1.00
Open	SPI to EPI	Day 3	-57.61	-104.46	-14.67	0.98	0.00	0.98
Open	EPI to SPI	Overall	62.65	40.52	92.50	1.00	1.00	0.00
Open	EPI to SPI	Day 0	15.96	5.70	28.57	0.87	0.87	0.00
Open	EPI to SPI	Day 1	85.40	53.48	127.22	1.00	1.00	0.00
Open	EPI to SPI	Day 2	94.45	51.55	144.49	1.00	1.00	0.00
Open	EPI to SPI	Day 3	54.96	16.93	102.70	1.00	1.00	0.00

4.2.2 by Surgical Types

Figure 19

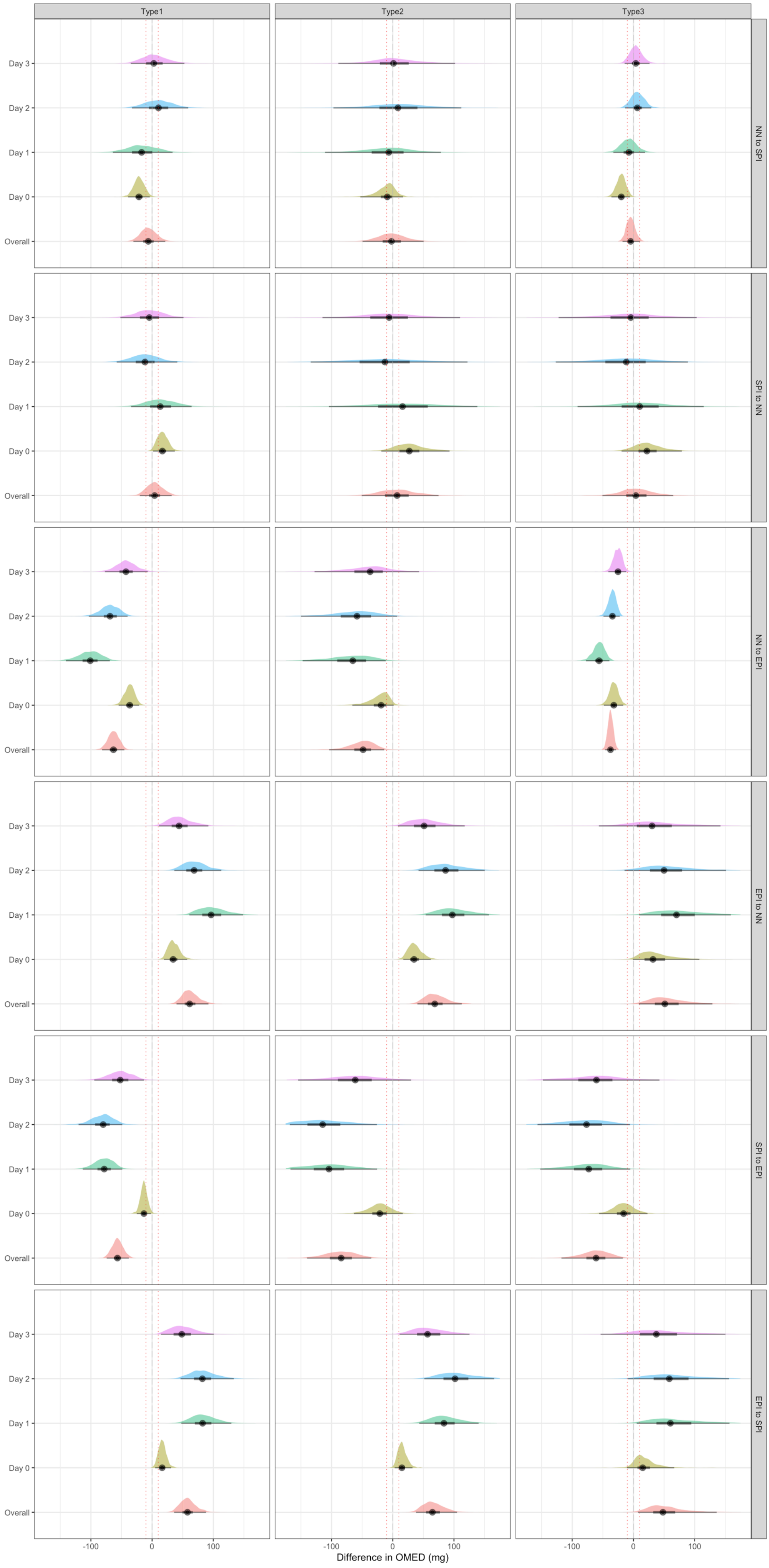

Type1
Type2
Type3
NN to SPI
SPI to NN
NN to EPI
EPI to NN
SPI to EPI
EPI to SPI
Day 3
Day 2
Day 1
Day 0
Overall
-100
0
100
Difference in OMED (mg)

1 The Data

2 Goals of the analysis

3 The Model

4 Adjusted OMED requirements

4.1 Comparisons of adjusted posterior OMED requirements

4.2 Comparisons of differences in OMED requirements if one were to have a different analgesic technique

4.2.1 by Surgical Approaches

4.2.2 by Surgical Types

5 Software and packages used

Table 8

SurgType	Diff	Day	Median	CI95_low	CI95_high	Pr(|Diff|>=10mg)	Pr(Diff>=10mg)	Pr(Diff<=-10mg)	Pr(-10mg
Type1	NN to SPI	Overall	-6.23	-31.12	19.95	0.50	0.11	0.38	
Type1	NN to SPI	Day 0	-21.49	-38.47	-3.54	0.90	0.00	0.90	
Type1	NN to SPI	Day 1	-17.12	-63.53	33.75	0.76	0.15	0.61	
Type1	NN to SPI	Day 2	10.42	-34.02	56.59	0.69	0.51	0.18	
Type1	NN to SPI	Day 3	2.79	-38.27	47.40	0.61	0.36	0.25	
Type1	SPI to NN	Overall	4.06	-21.41	30.40	0.47	0.33	0.14	
Type1	SPI to NN	Day 0	16.82	-0.53	33.74	0.78	0.78	0.00	
Type1	SPI to NN	Day 1	13.27	-36.88	61.31	0.72	0.56	0.16	
Type1	SPI to NN	Day 2	-11.70	-61.25	37.26	0.70	0.18	0.53	
Type1	SPI to NN	Day 3	-4.49	-55.85	45.93	0.68	0.27	0.41	
Type1	NN to EPI	Overall	-63.12	-82.00	-46.42	1.00	0.00	1.00	
Type1	NN to EPI	Day 0	-36.52	-53.20	-20.44	1.00	0.00	1.00	
Type1	NN to EPI	Day 1	-100.99	-138.72	-67.33	1.00	0.00	1.00	
Type1	NN to EPI	Day 2	-68.73	-103.63	-40.00	1.00	0.00	1.00	
Type1	NN to EPI	Day 3	-42.70	-78.54	-9.26	0.97	0.00	0.97	
Type1	EPI to NN	Overall	61.32	38.76	90.41	1.00	1.00	0.00	
Type1	EPI to NN	Day 0	34.40	18.25	55.08	1.00	1.00	0.00	
Type1	EPI to NN	Day 1	96.49	59.23	146.67	1.00	1.00	0.00	
Type1	EPI to NN	Day 2	68.68	34.97	110.59	1.00	1.00	0.00	
Type1	EPI	Day 3	44.07	10.15	90.15	0.98	0.98	0.00	

	to NN							
Type1	SPI to EPI	Overall	-56.55	-74.40	-38.52	1.00	0.00	1.00
Type1	SPI to EPI	Day 0	-13.33	-24.36	-1.33	0.74	0.00	0.74
Type1	SPI to EPI	Day 1	-78.19	-114.24	-50.20	1.00	0.00	1.00
Type1	SPI to EPI	Day 2	-79.90	-117.68	-47.07	1.00	0.00	1.00
Type1	SPI to EPI	Day 3	-52.03	-95.98	-16.37	0.99	0.00	0.98
Type1	EPI to SPI	Overall	57.80	34.98	86.22	1.00	1.00	0.00
Type1	EPI to SPI	Day 0	16.45	4.28	29.45	0.85	0.85	0.00
Type1	EPI to SPI	Day 1	82.48	44.39	124.43	1.00	1.00	0.00
Type1	EPI to SPI	Day 2	82.30	43.33	129.20	1.00	1.00	0.00
Type1	EPI to SPI	Day 3	48.51	11.25	95.15	0.99	0.99	0.00
Type2	NN to SPI	Overall	-2.10	-51.33	47.48	0.66	0.29	0.36
Type2	NN to SPI	Day 0	-8.96	-45.81	20.28	0.52	0.06	0.47
Type2	NN to SPI	Day 1	-6.64	-114.33	81.18	0.79	0.32	0.47
Type2	NN to SPI	Day 2	9.24	-111.95	122.12	0.82	0.49	0.33
Type2	NN to SPI	Day 3	1.18	-89.07	113.15	0.76	0.40	0.37
Type2	SPI to NN	Overall	7.07	-53.23	71.52	0.75	0.46	0.28
Type2	SPI to NN	Day 0	27.02	-20.73	90.37	0.81	0.76	0.05
Type2	SPI to NN	Day 1	18.10	-123.03	153.37	0.87	0.55	0.32
Type2	SPI to NN	Day 2	-13.72	-158.09	135.36	0.87	0.34	0.52
Type2	SPI to NN	Day 3	-5.89	-104.79	128.02	0.83	0.36	0.47

Type2	NN to EPI	Overall	-48.46	-99.64	-11.84	0.98	0.00	0.98
Type2	NN to EPI	Day 0	-19.02	-63.03	2.32	0.77	0.01	0.77
Type2	NN to EPI	Day 1	-65.90	-152.53	-6.45	0.98	0.00	0.98
Type2	NN to EPI	Day 2	-59.45	-160.56	11.19	0.96	0.02	0.94
Type2	NN to EPI	Day 3	-37.53	-132.07	57.44	0.90	0.09	0.81
Type2	EPI to NN	Overall	68.59	37.09	107.73	1.00	1.00	0.00
Type2	EPI to NN	Day 0	34.78	17.12	62.09	0.99	0.99	0.00
Type2	EPI to NN	Day 1	97.86	50.21	159.91	1.00	1.00	0.00
Type2	EPI to NN	Day 2	86.57	38.23	151.17	1.00	1.00	0.00
Type2	EPI to NN	Day 3	51.20	6.61	113.44	0.97	0.97	0.00
Type2	SPI to EPI	Overall	-84.32	-141.20	-35.16	1.00	0.00	1.00
Type2	SPI to EPI	Day 0	-21.19	-63.59	16.33	0.79	0.04	0.75
Type2	SPI to EPI	Day 1	-110.29	-236.48	-27.90	1.00	0.00	0.99
Type2	SPI to EPI	Day 2	-127.58	-252.64	-25.25	0.99	0.00	0.99
Type2	SPI to EPI	Day 3	-62.29	-173.11	25.62	0.94	0.05	0.89
Type2	EPI to SPI	Overall	64.79	35.54	100.72	1.00	1.00	0.00
Type2	EPI to SPI	Day 0	14.89	2.08	30.79	0.77	0.77	0.00
Type2	EPI to SPI	Day 1	83.60	41.23	138.99	1.00	1.00	0.00
Type2	EPI to SPI	Day 2	103.29	46.32	174.28	1.00	1.00	0.00
Type2	EPI to SPI	Day 3	56.89	10.39	126.05	0.98	0.98	0.00
Type3	NN to SPI	Overall	-4.57	-18.59	9.49	0.25	0.03	0.22

Type3	NN to SPI	Day 0	-19.82	-36.09	-5.94	0.91	0.00	0.91
Type3	NN to SPI	Day 1	-7.49	-32.82	18.94	0.52	0.09	0.43
Type3	NN to SPI	Day 2	6.38	-15.75	26.64	0.43	0.38	0.05
Type3	NN to SPI	Day 3	3.84	-15.14	25.33	0.34	0.27	0.07
Type3	SPI to NN	Overall	3.90	-51.63	64.28	0.69	0.41	0.28
Type3	SPI to NN	Day 0	21.97	-22.32	76.16	0.78	0.72	0.06
Type3	SPI to NN	Day 1	10.56	-95.90	126.26	0.82	0.51	0.32
Type3	SPI to NN	Day 2	-11.82	-135.00	100.69	0.85	0.33	0.52
Type3	SPI to NN	Day 3	-4.04	-138.29	118.59	0.82	0.37	0.45
Type3	NN to EPI	Overall	-37.55	-46.17	-28.61	1.00	0.00	1.00
Type3	NN to EPI	Day 0	-32.11	-47.35	-16.15	1.00	0.00	1.00
Type3	NN to EPI	Day 1	-56.15	-74.32	-37.08	1.00	0.00	1.00
Type3	NN to EPI	Day 2	-34.32	-47.70	-21.31	1.00	0.00	1.00
Type3	NN to EPI	Day 3	-25.19	-39.75	-10.96	0.98	0.00	0.98
Type3	EPI to NN	Overall	51.75	5.06	128.00	0.98	0.97	0.00
Type3	EPI to NN	Day 0	32.13	-4.36	106.01	0.90	0.88	0.01
Type3	EPI to NN	Day 1	74.21	-6.36	208.91	0.98	0.98	0.01
Type3	EPI to NN	Day 2	52.25	-32.04	175.99	0.93	0.89	0.03
Type3	EPI to NN	Day 3	32.46	-61.36	185.55	0.86	0.72	0.14
Type3	SPI to EPI	Overall	-61.05	-115.38	-15.61	0.99	0.00	0.99
Type3	SPI to EPI	Day 0	-16.05	-56.48	21.22	0.72	0.08	0.64

Type3	SPI to EPI	Day 1	-74.02	-167.93	-0.44	0.98	0.01	0.97
Type3	SPI to EPI	Day 2	-78.99	-182.82	9.19	0.98	0.01	0.97
Type3	SPI to EPI	Day 3	-62.88	-205.14	44.89	0.96	0.06	0.90
Type3	EPI to SPI	Overall	48.92	-2.88	128.01	0.97	0.96	0.00
Type3	EPI to SPI	Day 0	15.13	-14.88	61.23	0.68	0.66	0.03
Type3	EPI to SPI	Day 1	63.20	-3.40	186.92	0.97	0.96	0.01
Type3	EPI to SPI	Day 2	62.24	-25.80	200.34	0.95	0.92	0.02
Type3	EPI to SPI	Day 3	39.90	-61.42	197.36	0.87	0.76	0.10

5 Software and packages used

The model was implemented with the brms package in R which provides an interface to fit Bayesian generalized multivariate multilevel models using the probabilistic programming language Stan. cmdstanr package was used to interface with Stan from R. Packages used and version details are provided below.

```
## [1] "Fri Jul 22 15:06:35 2022"
```

```
## R version 4.2.1 (2022-06-23)
## Platform: x86_64-apple-darwin17.0 (64-bit)
## Running under: macOS Big Sur ... 10.16
##
## Matrix products: default
## BLAS:   /Library/Frameworks/R.framework/Versions/4.2/Resources/lib/libRblas.0.dylib
## LAPACK: /Library/Frameworks/R.framework/Versions/4.2/Resources/lib/libRlapack.dylib
##
## locale:
## [1] en_AU.UTF-8/en_AU.UTF-8/en_AU.UTF-8/C/en_AU.UTF-8/en_AU.UTF-8
##
## attached base packages:
## [1] splines   stats     graphics  grDevices utils     datasets  methods
## [8] base
##
## other attached packages:
##  [1] tidybayes_3.0.2    nlme_3.1-158       gridExtra_2.3      magrittr_2.0.3
##  [5] lubridate_1.8.0    readxl_1.4.0       skimr_2.1.4        tictoc_1.0.1
##  [9] bayesplot_1.9.0    bayestestR_0.12.1  quantreg_5.93      SparseM_1.81
## [13] brms_2.17.0        Rcpp_1.0.9         DT_0.23            summarytools_1.0.1
## [17] plotrix_3.8-2      forcats_0.5.1      stringr_1.4.0      dplyr_1.0.9
## [21] purrr_0.3.4        readr_2.1.2        tidyr_1.2.0        tibble_3.1.7
## [25] ggplot2_3.3.6      tidyverse_1.3.2
##
## loaded via a namespace (and not attached):
##   [1] backports_1.4.1      plyr_1.8.7           igraph_1.3.3
##   [4] repr_1.1.4           svUnit_1.0.6         crosstalk_1.2.0
##   [7] rstantools_2.2.0     inline_0.3.19        pryr_0.1.5
##  [10] digest_0.6.29        htmltools_0.5.3      magick_2.7.3
##  [13] fansi_1.0.3          checkmate_2.1.0      googlesheets4_1.0.0
##  [16] tzdb_0.3.0           modelr_0.1.8         RcppParallel_5.1.5
##  [19] matrixStats_0.62.0   xts_0.12.1           prettyunits_1.1.1
##  [22] colorspace_2.0-3     rvest_1.0.2          ggdist_3.1.1
##  [25] haven_2.5.0          xfun_0.31            tcltk_4.2.1
##  [28] callr_3.7.1          crayon_1.5.1         jsonlite_1.8.0
##  [31] survival_3.3-1       zoo_1.8-10           glue_1.6.2
##  [34] gtable_0.3.0         gargle_1.2.0         MatrixModels_0.5-0
##  [37] distributional_0.3.0 pkgbuild_1.3.1       rstan_2.21.5
##  [40] abind_1.4-5          rapportools_1.1      scales_1.2.0
##  [43] mvtnorm_1.1-3        DBI_1.1.3            ggeffects_1.1.2
##  [46] miniUI_0.1.1.1       xtable_1.8-4         stats4_4.2.1
##  [49] StanHeaders_2.21.0-7 datawizard_0.4.1     htmlwidgets_1.5.4
##  [52] httr_1.4.3           threejs_0.3.3        arrayhelpers_1.1-0
##  [55] posterior_1.2.2      ellipsis_0.3.2       pkgconfig_2.0.3
##  [58] loo_2.5.1            farver_2.1.1         sass_0.4.2
##  [61] dbplyr_2.2.1         utf8_1.2.2           labeling_0.4.2
##  [64] tidyselect_1.1.2     rlang_1.0.4          reshape2_1.4.4
##  [67] later_1.3.0          munsell_0.5.0        cellranger_1.1.0
##  [70] tools_4.2.1          cachem_1.0.6         cli_3.3.0
##  [73] generics_0.1.3       broom_1.0.0          ggridges_0.5.3
##  [76] evaluate_0.15        fastmap_1.1.0        yaml_2.3.5
##  [79] processx_3.7.0       knitr_1.39           fs_1.5.2
##  [82] pander_0.6.5         mime_0.12            ggpol_0.0.7
##  [85] xml2_1.3.3           compiler_4.2.1       shinythemes_1.2.0
##  [88] rstudioapi_0.13      reprex_2.0.1         bslib_0.4.0
##  [91] stringi_1.7.8        highr_0.9            ps_1.7.1
##  [94] Brobdingnag_1.2-7    lattice_0.20-45      Matrix_1.4-1
##  [97] markdown_1.1         shinyjs_2.1.0        tensorA_0.36.2
## [100] vctrs_0.4.1          pillar_1.8.0         lifecycle_1.0.1
## [103] jquerylib_0.1.4      bridgesampling_1.1-2 data.table_1.14.2
## [106] insight_0.18.0       httpuv_1.6.5         R6_2.5.1
## [109] promises_1.2.0.1     codetools_0.2-18     colourpicker_1.1.1
## [112] MASS_7.3-58          gtools_3.9.3         assertthat_0.2.1
## [115] withr_2.5.0          shinystan_2.6.0      mgcv_1.8-40
## [118] parallel_4.2.1       hms_1.1.1            grid_4.2.1
## [121] coda_0.19-4          cmdstanr_0.5.2       rmarkdown_2.14
## [124] googledrive_2.0.0    shiny_1.7.2          base64enc_0.1-3
## [127] dygraphs_1.1.1.6
```

```
## 1613.956 sec elapsed
```
