# Supplementary material for: Postoperative analgesia for upper gastrointestinal surgery: a retrospective cohort analysis
Source: Perioper Med (Lond). 2023 Jul 18;12:40. doi: 10.1186/s13741-023-00324-0 (PMC10355044; doi:10.1186/s13741-023-00324-0)
Supplement: Supplementary file 1 — Additional file 1. a Post-operative morphine requirements. b. Post-operative rest pain. c Post-operative dynamic pain. [file 13741_2023_324_MOESM1_ESM.zip › Additional file 1b.pdf]

# Supplementary Material 1b

Code ▾

## Post-operative Rest Pain

# 1 The Data

The data are ***resting pain scores*** on day 0, 1, 2 and 3 after surgery. They are on an ordinal scale from 0 to 10. To avoid mistaken them as on a numerical scale, I've labelled them in words from "zero" to "ten", so arithmic calculations aren't possible.
The data displayed below in the frequency table is the count of a particular pain score on a particular day.

PSr: Pain Score (resting)
NN: Non-neuraxial
SPI: Intrathecal Morphine
EPI: Thoracic Epidural Analgesia

### Table 1

```
##        Days
## PSr       0   1   2   3
##   Zero  105  45  62  85
##   One    39  41  49  49
##   Two    46  56  55  69
##   Three  28  52  63  49
##   Four   28  66  62  36
##   Five   33  63  50  34
##   Six    29  36  26  22
##   Seven  25  19  17  14
##   Eight  30  20  17   7
##   Nine   10  10   2   3
##   Ten     9  11   2   3
```

The data is again displayed graphically below.

The first plot shows a bar graph of the proportion of counts for resting pain scores over different post operative days across different analgesic techniques. The red bars represent the pain score with the most counts.

### Figure 1

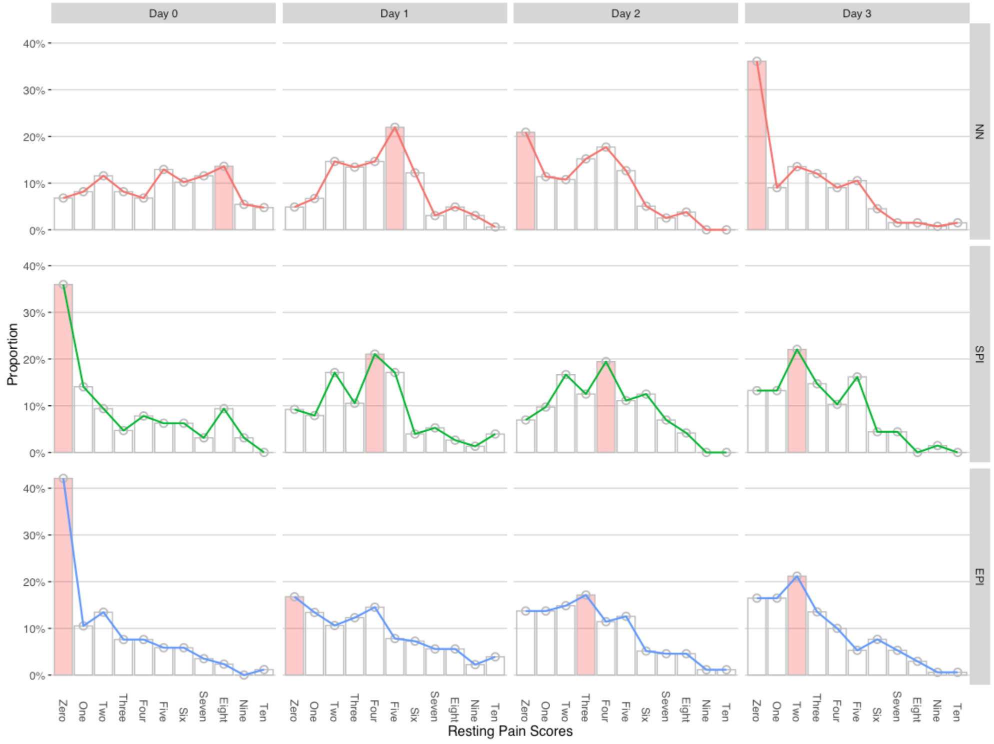


The plot below shows the same information, this time with cumulative proportion. The vertical line shows the proportion of patients scoring the respective resting PS. The PS with the largest proportion is represented by the red line.

## Figure 2

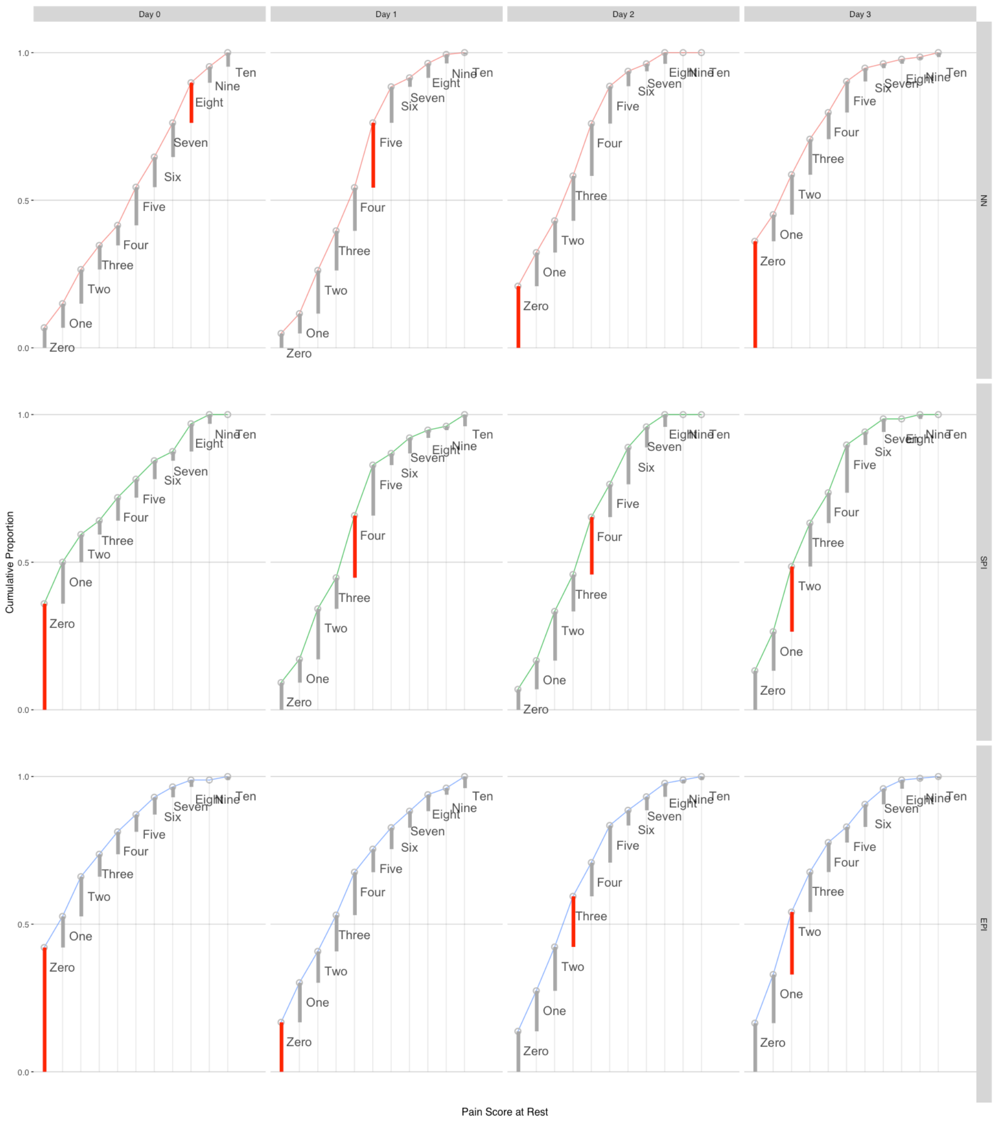


# 2 Goals of the analysis

We are interested in

1. Distribution of Pain scores at rest with different analgesic techniques over the post-operative period
2. Probability of poor pain control
3. Probability of poor pain control if our threshold is different

We consider pain score of 4 or less as adequate pain control.

# 3 The Model

The outcome variable resting pain scores (PSr) are ordinal such that pain score (PS) of $zero > one > two > three > \ldots > ten$. The interval between the pain scores are not necessarily of the same distance. That is, the increase in pain from a PS of 1 to 2 may not be of the same magnitude to that from a PS of 5 to 6. Hence, we will model PSr with an ordinal cumulative model.

The cumulative model assumes that the observed ordinal variable $Y$ originates from the categorization of a latent continuous variable $\tilde{Y}$, such that there are latent thresholds $\theta_k$ that partition the values into the $k+1$ observable ordered categories of $Y$ (i.e. ordered pain score categories of zero, one, two, three… and so on to a pain score category of ten). If we use $\eta$ to symbolise the predictor terms, such that $\eta = \beta X$, and $F$ being a cumulative distribution function, then

$$Pr(Y = k|\eta) = F(\theta_k - \eta) - F(\theta_{k-1} - \eta)$$

Note this cumulative model assumes that the predictor term $\eta$ is constant across the response categories. For our model, we will use the $logit$ link function for its ease of interpretation of the coefficients as odds ratios.

## 3.1 Model specifications

The data generating process for scoring a particular PS should be the same as for the OMED requirements. We will therefore use the same mixed effect structure for $\eta$ with the same predictor variables and interaction terms.

For the priors, we will set a common prior for each intercept which assigns probability to scoring the 11 categories of pain score more evenly. This choice is examined by comparing different prior values as shown below. To find a prior that roughly assigns equal probability to each of the pain score, we will compare different prior values from $\sim N(0, 0.5)$ to $\sim N(0, 11)$ in sequential sequence by 0.5. One can see that as the prior $\sigma$ increases from 0.5 to 11, the proportion of assignment of the pain score changes from assigning more on the extreme values to more in the middle. The bars are the data pain score distribution. The blue points/lines are the median of prior posterior draw predictions (without the influence of the data), and the error bars are 99% credible intervals for the prior prediction draws. A prior of $\sim N(0.5)$ seems to be reasonably spread out with coverage up to 50% proportion for each pain score.

**Figure 3**

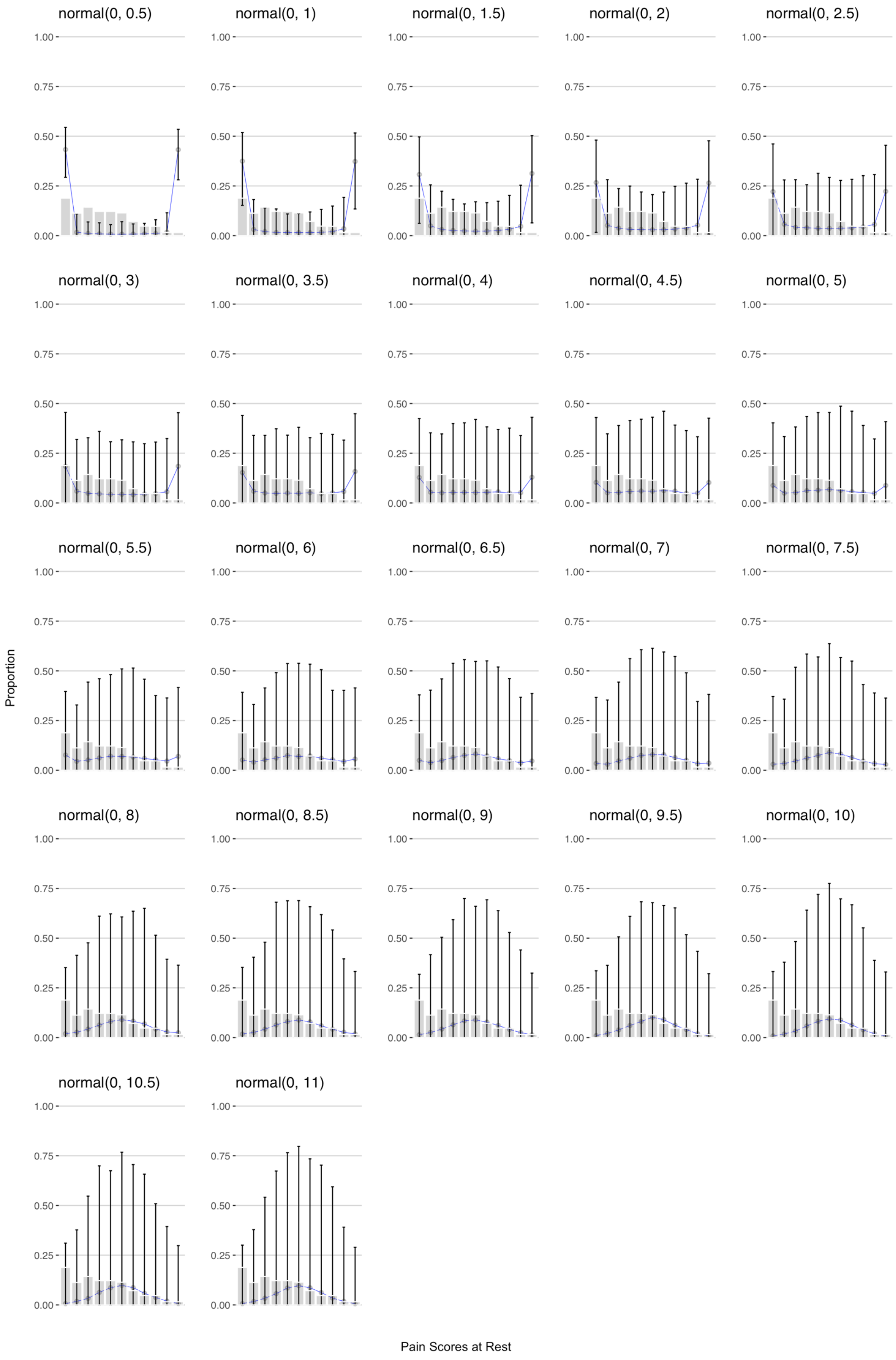


As for the $\beta$ effect, we will assign the prior to $\sim Normal(0, 0.5)$, which assigns prior probability to be higher towards zero difference, but still encompass reasonable probability difference effects. These are weakly informative priors that consist of reasonable, non-extreme probability assignments.

$$
\begin{aligned}
PS_i &\sim Categorical(\mathbf{p}) \\
p_1 &= q_1 \\
p_k &= q_k - q_{k-1} && for\ K > k > 1 \\
logit(q_k) &= \theta_k - \eta_i \\
\eta_i &= \beta_{ID[i]} \\
\theta_k &\sim Normal(0, 5) \\
\beta_j &\sim Normal(0, 0.5)
\end{aligned}
$$

## 3.2 Prior predictive check

This is the same as the comparison plot above, zoomed in to the prior predictive distribution when we set the intercept prior to be $\sim Normal(0, 5)$.

It can be seen that the prior roughly evenly distributed the probability to each of the 11 pain scores and covers a reasonable probability of scoring a particular pain score. There is also no unreasonable values such as negative proportions.

**Figure 4**

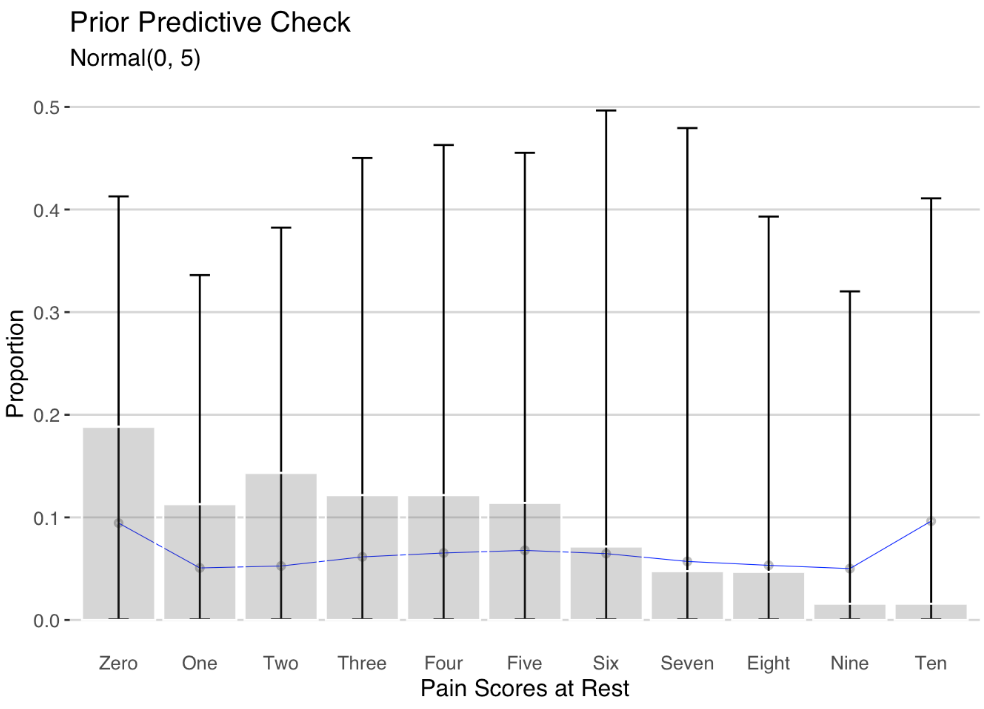


## 3.3 Posterior predictive check

It can be seen from the plot that the posteriors describes the data very well.

**Figure 5**

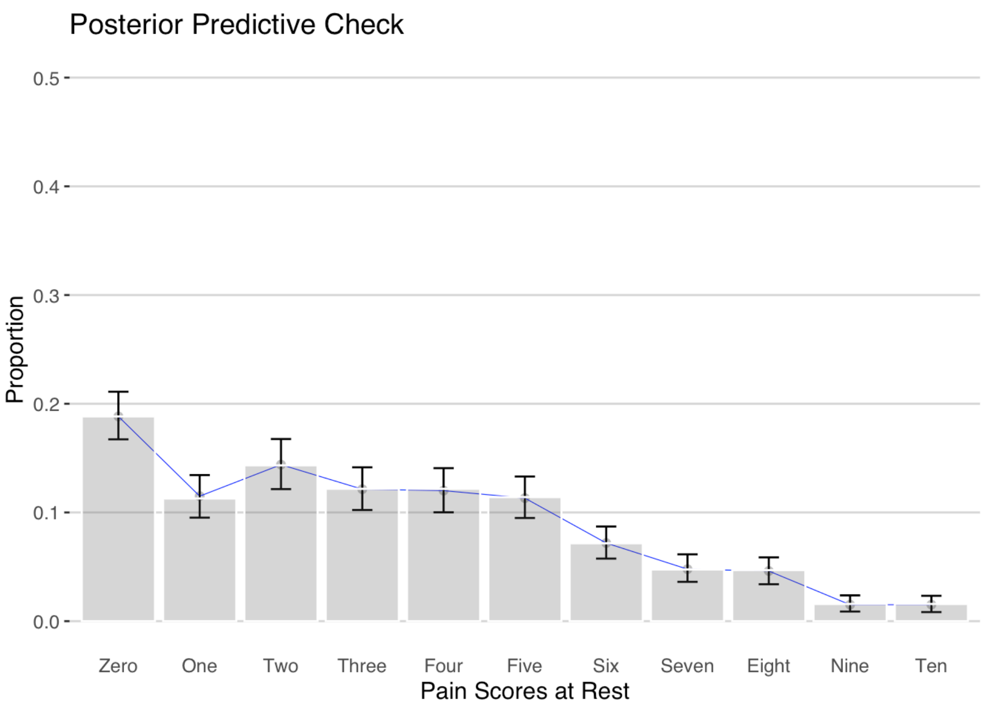


# 4 Posterior Distributions of Pain Scores at Rest

## 4.1 Adjusted resting pain scores

### 4.1.1 Overal distribution of pain scores at rest over the 4 post-operative time points and with different analgesic techniques

The plot shows the distribution of post-op resting pain scores for a typical patient who is

```
- 60 years of age
- Female
- ASA = 3
- Surgical Approach = Open
- Surgical type = Type 1
```

**Figure 6**

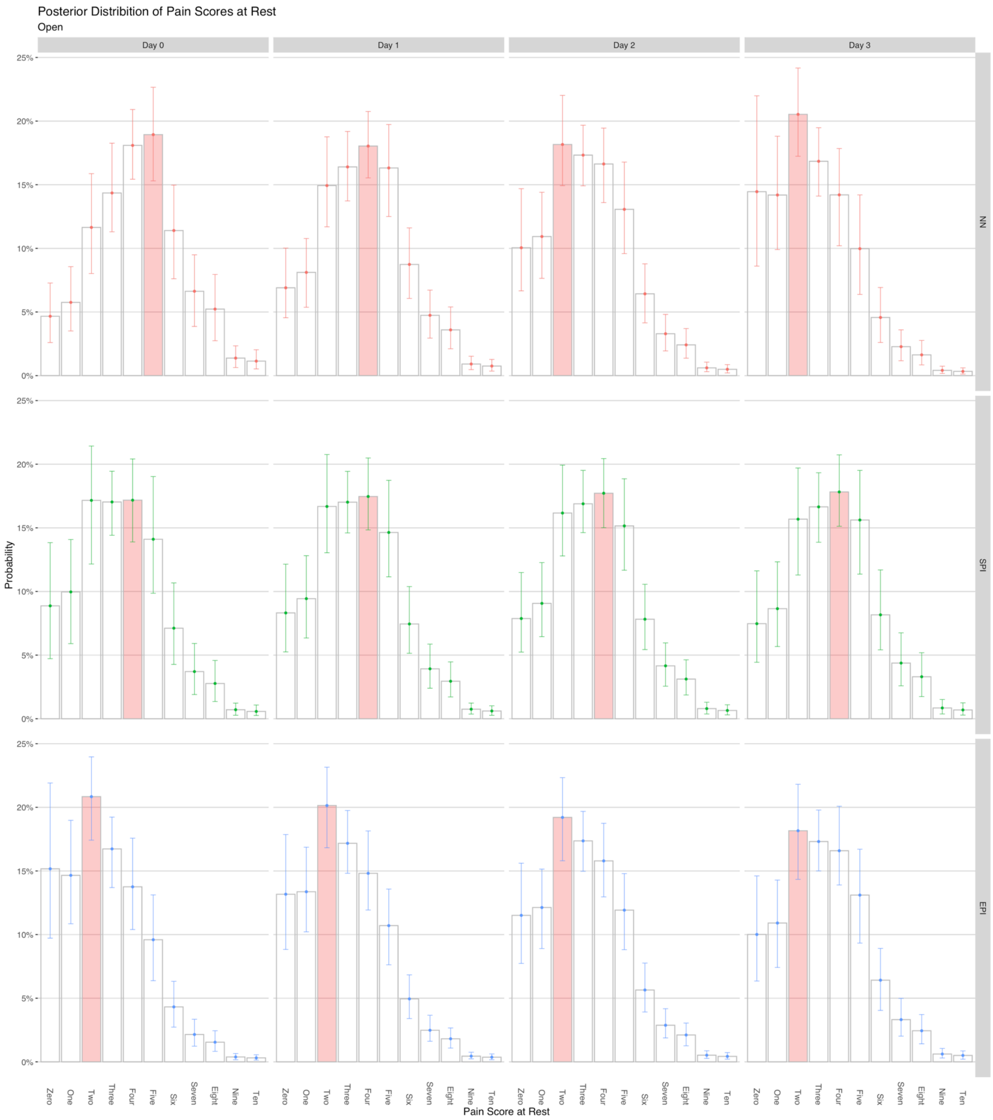


## Table 2

| Days | Group | PS | Probability | 95%CrI.upper | 95%CrI.lower | 50%CrI.upper | 50%CrI.lo[illegible] |
|---|---|---|---|---|---|---|---|
| 0 | NN | Zero | 0.05 | 0.07 | 0.03 | 0.05 | 0 |
| 0 | NN | One | 0.06 | 0.09 | 0.04 | 0.06 | 0 |
| 0 | NN | Two | 0.12 | 0.16 | 0.08 | 0.13 | 0 |
| 0 | NN | Three | 0.14 | 0.18 | 0.11 | 0.16 | 0 |
| 0 | NN | Four | 0.18 | 0.21 | 0.15 | 0.19 | 0 |
| 0 | NN | Five | 0.19 | 0.23 | 0.15 | 0.20 | 0 |
| 0 | NN | Six | 0.11 | 0.15 | 0.08 | 0.13 | 0 |
| 0 | NN | Seven | 0.07 | 0.09 | 0.04 | 0.08 | 0 |
| 0 | NN | Eight | 0.05 | 0.08 | 0.03 | 0.06 | 0 |
| 0 | NN | Nine | 0.01 | 0.02 | 0.01 | 0.02 | 0 |
| 0 | NN | Ten | 0.01 | 0.02 | 0.01 | 0.01 | 0 |
| 0 | SPI | Zero | 0.09 | 0.14 | 0.05 | 0.10 | 0 |

| | | | | | | | |
|---|---|---|---|---|---|---|---|
| 0 | SPI | One | 0.10 | 0.14 | 0.06 | 0.11 | 0 |
| 0 | SPI | Two | 0.17 | 0.21 | 0.12 | 0.19 | 0 |
| 0 | SPI | Three | 0.17 | 0.19 | 0.14 | 0.18 | 0 |
| 0 | SPI | Four | 0.17 | 0.20 | 0.14 | 0.18 | 0 |
| 0 | SPI | Five | 0.14 | 0.19 | 0.10 | 0.15 | 0 |
| 0 | SPI | Six | 0.07 | 0.11 | 0.04 | 0.08 | 0 |
| 0 | SPI | Seven | 0.04 | 0.06 | 0.02 | 0.04 | 0 |
| 0 | SPI | Eight | 0.03 | 0.05 | 0.01 | 0.03 | 0 |
| 0 | SPI | Nine | 0.01 | 0.01 | 0.00 | 0.01 | 0 |
| 0 | SPI | Ten | 0.01 | 0.01 | 0.00 | 0.01 | 0 |
| 0 | EPI | Zero | 0.15 | 0.22 | 0.10 | 0.17 | 0 |
| 0 | EPI | One | 0.15 | 0.19 | 0.11 | 0.16 | 0 |
| 0 | EPI | Two | 0.21 | 0.24 | 0.17 | 0.22 | 0 |
| 0 | EPI | Three | 0.17 | 0.19 | 0.14 | 0.18 | 0 |
| 0 | EPI | Four | 0.14 | 0.18 | 0.10 | 0.15 | 0 |
| 0 | EPI | Five | 0.10 | 0.13 | 0.06 | 0.11 | 0 |
| 0 | EPI | Six | 0.04 | 0.06 | 0.03 | 0.05 | 0 |
| 0 | EPI | Seven | 0.02 | 0.03 | 0.01 | 0.02 | 0 |
| 0 | EPI | Eight | 0.02 | 0.02 | 0.01 | 0.02 | 0 |
| 0 | EPI | Nine | 0.00 | 0.01 | 0.00 | 0.00 | 0 |
| 0 | EPI | Ten | 0.00 | 0.01 | 0.00 | 0.00 | 0 |
| 1 | NN | Zero | 0.07 | 0.10 | 0.05 | 0.08 | 0 |
| 1 | NN | One | 0.08 | 0.11 | 0.05 | 0.09 | 0 |
| 1 | NN | Two | 0.15 | 0.19 | 0.12 | 0.16 | 0 |
| 1 | NN | Three | 0.16 | 0.19 | 0.14 | 0.17 | 0 |
| 1 | NN | Four | 0.18 | 0.21 | 0.16 | 0.19 | 0 |
| 1 | NN | Five | 0.16 | 0.20 | 0.13 | 0.18 | 0 |
| 1 | NN | Six | 0.09 | 0.12 | 0.06 | 0.10 | 0 |
| 1 | NN | Seven | 0.05 | 0.07 | 0.03 | 0.05 | 0 |
| 1 | NN | Eight | 0.04 | 0.05 | 0.02 | 0.04 | 0 |
| 1 | NN | Nine | 0.01 | 0.02 | 0.00 | 0.01 | 0 |
| 1 | NN | Ten | 0.01 | 0.01 | 0.00 | 0.01 | 0 |
| 1 | SPI | Zero | 0.08 | 0.12 | 0.05 | 0.09 | 0 |
| 1 | SPI | One | 0.09 | 0.13 | 0.06 | 0.10 | 0 |
| 1 | SPI | Two | 0.17 | 0.21 | 0.13 | 0.18 | 0 |
| 1 | SPI | Three | 0.17 | 0.19 | 0.15 | 0.18 | 0 |
| 1 | SPI | Four | 0.17 | 0.20 | 0.15 | 0.18 | 0 |
| 1 | SPI | Five | 0.15 | 0.19 | 0.11 | 0.16 | 0 |

| | | | | | | | |
|---|---|---|---|---|---|---|---|
| 1 | SPI | Six | 0.07 | 0.10 | 0.05 | 0.08 | 0 |
| 1 | SPI | Seven | 0.04 | 0.06 | 0.02 | 0.05 | 0 |
| 1 | SPI | Eight | 0.03 | 0.04 | 0.02 | 0.03 | 0 |
| 1 | SPI | Nine | 0.01 | 0.01 | 0.00 | 0.01 | 0 |
| 1 | SPI | Ten | 0.01 | 0.01 | 0.00 | 0.01 | 0 |
| 1 | EPI | Zero | 0.13 | 0.18 | 0.09 | 0.14 | 0 |
| 1 | EPI | One | 0.13 | 0.17 | 0.10 | 0.14 | 0 |
| 1 | EPI | Two | 0.20 | 0.23 | 0.17 | 0.21 | 0 |
| 1 | EPI | Three | 0.17 | 0.20 | 0.15 | 0.18 | 0 |
| 1 | EPI | Four | 0.15 | 0.18 | 0.12 | 0.16 | 0 |
| 1 | EPI | Five | 0.11 | 0.14 | 0.08 | 0.11 | 0 |
| 1 | EPI | Six | 0.05 | 0.07 | 0.03 | 0.05 | 0 |
| 1 | EPI | Seven | 0.02 | 0.04 | 0.02 | 0.03 | 0 |
| 1 | EPI | Eight | 0.02 | 0.03 | 0.01 | 0.02 | 0 |
| 1 | EPI | Nine | 0.00 | 0.01 | 0.00 | 0.01 | 0 |
| 1 | EPI | Ten | 0.00 | 0.01 | 0.00 | 0.00 | 0 |
| 2 | NN | Zero | 0.10 | 0.15 | 0.07 | 0.11 | 0 |
| 2 | NN | One | 0.11 | 0.14 | 0.08 | 0.12 | 0 |
| 2 | NN | Two | 0.18 | 0.22 | 0.15 | 0.20 | 0 |
| 2 | NN | Three | 0.17 | 0.20 | 0.15 | 0.18 | 0 |
| 2 | NN | Four | 0.17 | 0.19 | 0.14 | 0.18 | 0 |
| 2 | NN | Five | 0.13 | 0.17 | 0.10 | 0.14 | 0 |
| 2 | NN | Six | 0.06 | 0.09 | 0.04 | 0.07 | 0 |
| 2 | NN | Seven | 0.03 | 0.05 | 0.02 | 0.04 | 0 |
| 2 | NN | Eight | 0.02 | 0.04 | 0.01 | 0.03 | 0 |
| 2 | NN | Nine | 0.01 | 0.01 | 0.00 | 0.01 | 0 |
| 2 | NN | Ten | 0.00 | 0.01 | 0.00 | 0.01 | 0 |
| 2 | SPI | Zero | 0.08 | 0.11 | 0.05 | 0.09 | 0 |
| 2 | SPI | One | 0.09 | 0.12 | 0.06 | 0.10 | 0 |
| 2 | SPI | Two | 0.16 | 0.20 | 0.13 | 0.17 | 0 |
| 2 | SPI | Three | 0.17 | 0.20 | 0.15 | 0.18 | 0 |
| 2 | SPI | Four | 0.18 | 0.20 | 0.15 | 0.19 | 0 |
| 2 | SPI | Five | 0.15 | 0.19 | 0.12 | 0.16 | 0 |
| 2 | SPI | Six | 0.08 | 0.11 | 0.05 | 0.09 | 0 |
| 2 | SPI | Seven | 0.04 | 0.06 | 0.03 | 0.05 | 0 |
| 2 | SPI | Eight | 0.03 | 0.05 | 0.02 | 0.03 | 0 |
| 2 | SPI | Nine | 0.01 | 0.01 | 0.00 | 0.01 | 0 |
| 2 | SPI | Ten | 0.01 | 0.01 | 0.00 | 0.01 | 0 |

| | | | | | | | |
|---|---|---|---|---|---|---|---|
| 2 | EPI | Zero | 0.12 | 0.16 | 0.08 | 0.13 | 0 |
| 2 | EPI | One | 0.12 | 0.15 | 0.09 | 0.13 | 0 |
| 2 | EPI | Two | 0.19 | 0.22 | 0.16 | 0.20 | 0 |
| 2 | EPI | Three | 0.17 | 0.20 | 0.15 | 0.18 | 0 |
| 2 | EPI | Four | 0.16 | 0.19 | 0.13 | 0.17 | 0 |
| 2 | EPI | Five | 0.12 | 0.15 | 0.09 | 0.13 | 0 |
| 2 | EPI | Six | 0.06 | 0.08 | 0.04 | 0.06 | 0 |
| 2 | EPI | Seven | 0.03 | 0.04 | 0.02 | 0.03 | 0 |
| 2 | EPI | Eight | 0.02 | 0.03 | 0.01 | 0.02 | 0 |
| 2 | EPI | Nine | 0.01 | 0.01 | 0.00 | 0.01 | 0 |
| 2 | EPI | Ten | 0.00 | 0.01 | 0.00 | 0.00 | 0 |
| 3 | NN | Zero | 0.14 | 0.22 | 0.09 | 0.16 | 0 |
| 3 | NN | One | 0.14 | 0.19 | 0.10 | 0.15 | 0 |
| 3 | NN | Two | 0.21 | 0.24 | 0.17 | 0.22 | 0 |
| 3 | NN | Three | 0.17 | 0.19 | 0.14 | 0.18 | 0 |
| 3 | NN | Four | 0.14 | 0.18 | 0.10 | 0.16 | 0 |
| 3 | NN | Five | 0.10 | 0.14 | 0.06 | 0.11 | 0 |
| 3 | NN | Six | 0.05 | 0.07 | 0.03 | 0.05 | 0 |
| 3 | NN | Seven | 0.02 | 0.04 | 0.01 | 0.03 | 0 |
| 3 | NN | Eight | 0.02 | 0.03 | 0.01 | 0.02 | 0 |
| 3 | NN | Nine | 0.00 | 0.01 | 0.00 | 0.00 | 0 |
| 3 | NN | Ten | 0.00 | 0.01 | 0.00 | 0.00 | 0 |
| 3 | SPI | Zero | 0.07 | 0.12 | 0.04 | 0.08 | 0 |
| 3 | SPI | One | 0.09 | 0.12 | 0.06 | 0.09 | 0 |
| 3 | SPI | Two | 0.16 | 0.20 | 0.11 | 0.17 | 0 |
| 3 | SPI | Three | 0.17 | 0.19 | 0.14 | 0.18 | 0 |
| 3 | SPI | Four | 0.18 | 0.21 | 0.15 | 0.19 | 0 |
| 3 | SPI | Five | 0.16 | 0.20 | 0.11 | 0.17 | 0 |
| 3 | SPI | Six | 0.08 | 0.12 | 0.05 | 0.09 | 0 |
| 3 | SPI | Seven | 0.04 | 0.07 | 0.03 | 0.05 | 0 |
| 3 | SPI | Eight | 0.03 | 0.05 | 0.02 | 0.04 | 0 |
| 3 | SPI | Nine | 0.01 | 0.02 | 0.00 | 0.01 | 0 |
| 3 | SPI | Ten | 0.01 | 0.01 | 0.00 | 0.01 | 0 |
| 3 | EPI | Zero | 0.10 | 0.15 | 0.06 | 0.11 | 0 |
| 3 | EPI | One | 0.11 | 0.14 | 0.07 | 0.12 | 0 |
| 3 | EPI | Two | 0.18 | 0.22 | 0.14 | 0.19 | 0 |
| 3 | EPI | Three | 0.17 | 0.20 | 0.15 | 0.18 | 0 |
| 3 | EPI | Four | 0.17 | 0.20 | 0.14 | 0.18 | 0 |

| | | | | | | | |
|---|---|---|---|---|---|---|---|
| 3 | EPI | Five | 0.13 | 0.17 | 0.09 | 0.14 | 0 |
| 3 | EPI | Six | 0.06 | 0.09 | 0.04 | 0.07 | 0 |
| 3 | EPI | Seven | 0.03 | 0.05 | 0.02 | 0.04 | 0 |
| 3 | EPI | Eight | 0.02 | 0.04 | 0.01 | 0.03 | 0 |
| 3 | EPI | Nine | 0.01 | 0.01 | 0.00 | 0.01 | 0 |
| 3 | EPI | Ten | 0.01 | 0.01 | 0.00 | 0.01 | 0 |

In comparison, we will plot the probability distribution of resting pain scores across the 4 day post-op period a typical patient who has **laparoscopic surgery** instead:

```
- 60 years of age
- Female
- ASA = 3
- Surgical Approach = Laparoscopic
- Surgical type = Type 1
```

## Figure 7

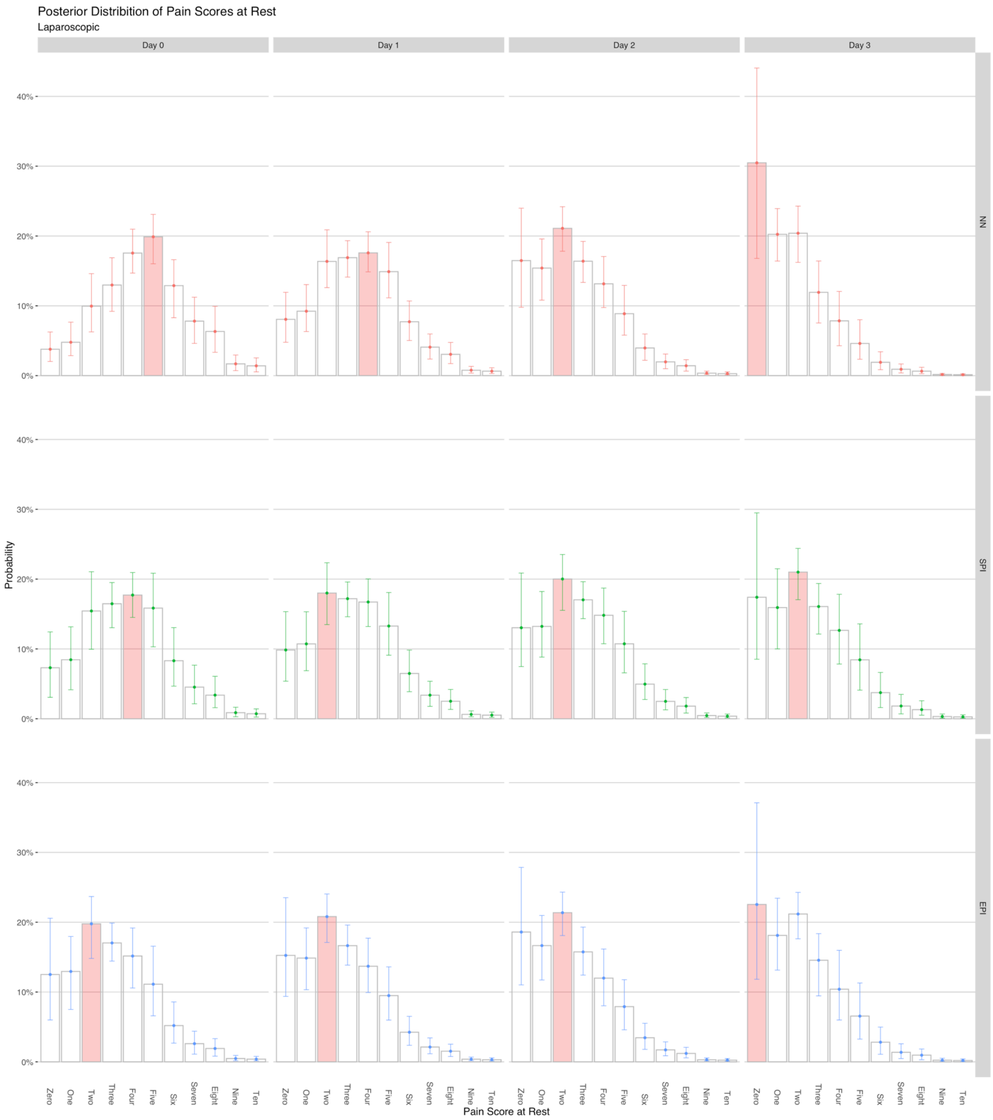


## Table 3

| Days | Group | PS | Probability | 95%CrI.upper | 95%CrI.lower | 50%CrI.upper | 50%CrI.low |
|---|---|---|---|---|---|---|---|
| 0 | NN | Zero | 0.04 | 0.06 | 0.02 | 0.04 | 0 |
| 0 | NN | One | 0.05 | 0.08 | 0.03 | 0.06 | 0 |
| 0 | NN | Two | 0.10 | 0.15 | 0.06 | 0.11 | 0 |
| 0 | NN | Three | 0.13 | 0.17 | 0.09 | 0.14 | 0 |
| 0 | NN | Four | 0.18 | 0.21 | 0.15 | 0.19 | 0 |
| 0 | NN | Five | 0.20 | 0.23 | 0.16 | 0.21 | 0 |
| 0 | NN | Six | 0.13 | 0.17 | 0.08 | 0.14 | 0 |
| 0 | NN | Seven | 0.08 | 0.11 | 0.05 | 0.09 | 0 |
| 0 | NN | Eight | 0.06 | 0.10 | 0.03 | 0.07 | 0 |
| 0 | NN | Nine | 0.02 | 0.03 | 0.01 | 0.02 | 0 |
| 0 | NN | Ten | 0.01 | 0.03 | 0.01 | 0.02 | 0 |
| 0 | SPI | Zero | 0.07 | 0.12 | 0.03 | 0.08 | 0 |

| | | | | | | | |
|---|---|---|---|---|---|---|---|
| 0 | SPI | One | 0.08 | 0.13 | 0.04 | 0.10 | 0 |
| 0 | SPI | Two | 0.15 | 0.21 | 0.10 | 0.17 | 0 |
| 0 | SPI | Three | 0.16 | 0.20 | 0.13 | 0.18 | 0 |
| 0 | SPI | Four | 0.18 | 0.21 | 0.15 | 0.19 | 0 |
| 0 | SPI | Five | 0.16 | 0.21 | 0.10 | 0.18 | 0 |
| 0 | SPI | Six | 0.08 | 0.13 | 0.05 | 0.09 | 0 |
| 0 | SPI | Seven | 0.05 | 0.08 | 0.02 | 0.05 | 0 |
| 0 | SPI | Eight | 0.03 | 0.06 | 0.02 | 0.04 | 0 |
| 0 | SPI | Nine | 0.01 | 0.02 | 0.00 | 0.01 | 0 |
| 0 | SPI | Ten | 0.01 | 0.01 | 0.00 | 0.01 | 0 |
| 0 | EPI | Zero | 0.13 | 0.21 | 0.06 | 0.15 | 0 |
| 0 | EPI | One | 0.13 | 0.18 | 0.07 | 0.15 | 0 |
| 0 | EPI | Two | 0.20 | 0.24 | 0.15 | 0.21 | 0 |
| 0 | EPI | Three | 0.17 | 0.20 | 0.14 | 0.18 | 0 |
| 0 | EPI | Four | 0.15 | 0.19 | 0.11 | 0.17 | 0 |
| 0 | EPI | Five | 0.11 | 0.17 | 0.07 | 0.13 | 0 |
| 0 | EPI | Six | 0.05 | 0.09 | 0.03 | 0.06 | 0 |
| 0 | EPI | Seven | 0.03 | 0.04 | 0.01 | 0.03 | 0 |
| 0 | EPI | Eight | 0.02 | 0.03 | 0.01 | 0.02 | 0 |
| 0 | EPI | Nine | 0.00 | 0.01 | 0.00 | 0.01 | 0 |
| 0 | EPI | Ten | 0.00 | 0.01 | 0.00 | 0.00 | 0 |
| 1 | NN | Zero | 0.08 | 0.12 | 0.05 | 0.09 | 0 |
| 1 | NN | One | 0.09 | 0.13 | 0.06 | 0.10 | 0 |
| 1 | NN | Two | 0.16 | 0.21 | 0.13 | 0.18 | 0 |
| 1 | NN | Three | 0.17 | 0.19 | 0.14 | 0.18 | 0 |
| 1 | NN | Four | 0.18 | 0.21 | 0.15 | 0.19 | 0 |
| 1 | NN | Five | 0.15 | 0.19 | 0.11 | 0.16 | 0 |
| 1 | NN | Six | 0.08 | 0.11 | 0.05 | 0.09 | 0 |
| 1 | NN | Seven | 0.04 | 0.06 | 0.02 | 0.05 | 0 |
| 1 | NN | Eight | 0.03 | 0.05 | 0.02 | 0.03 | 0 |
| 1 | NN | Nine | 0.01 | 0.01 | 0.00 | 0.01 | 0 |
| 1 | NN | Ten | 0.01 | 0.01 | 0.00 | 0.01 | 0 |
| 1 | SPI | Zero | 0.10 | 0.15 | 0.05 | 0.11 | 0 |
| 1 | SPI | One | 0.11 | 0.15 | 0.07 | 0.12 | 0 |
| 1 | SPI | Two | 0.18 | 0.22 | 0.13 | 0.20 | 0 |
| 1 | SPI | Three | 0.17 | 0.20 | 0.15 | 0.18 | 0 |
| 1 | SPI | Four | 0.17 | 0.20 | 0.13 | 0.18 | 0 |
| 1 | SPI | Five | 0.13 | 0.18 | 0.09 | 0.15 | 0 |

| | | | | | | | |
|---|---|---|---|---|---|---|---|
| 1 | SPI | Six | 0.06 | 0.10 | 0.04 | 0.07 | 0 |
| 1 | SPI | Seven | 0.03 | 0.05 | 0.02 | 0.04 | 0 |
| 1 | SPI | Eight | 0.03 | 0.04 | 0.01 | 0.03 | 0 |
| 1 | SPI | Nine | 0.01 | 0.01 | 0.00 | 0.01 | 0 |
| 1 | SPI | Ten | 0.01 | 0.01 | 0.00 | 0.01 | 0 |
| 1 | EPI | Zero | 0.15 | 0.24 | 0.09 | 0.17 | 0 |
| 1 | EPI | One | 0.15 | 0.19 | 0.10 | 0.16 | 0 |
| 1 | EPI | Two | 0.21 | 0.24 | 0.17 | 0.22 | 0 |
| 1 | EPI | Three | 0.17 | 0.20 | 0.14 | 0.18 | 0 |
| 1 | EPI | Four | 0.14 | 0.18 | 0.10 | 0.15 | 0 |
| 1 | EPI | Five | 0.09 | 0.14 | 0.06 | 0.11 | 0 |
| 1 | EPI | Six | 0.04 | 0.07 | 0.02 | 0.05 | 0 |
| 1 | EPI | Seven | 0.02 | 0.03 | 0.01 | 0.02 | 0 |
| 1 | EPI | Eight | 0.02 | 0.03 | 0.01 | 0.02 | 0 |
| 1 | EPI | Nine | 0.00 | 0.01 | 0.00 | 0.00 | 0 |
| 1 | EPI | Ten | 0.00 | 0.01 | 0.00 | 0.00 | 0 |
| 2 | NN | Zero | 0.16 | 0.24 | 0.10 | 0.18 | 0 |
| 2 | NN | One | 0.15 | 0.20 | 0.11 | 0.17 | 0 |
| 2 | NN | Two | 0.21 | 0.24 | 0.18 | 0.22 | 0 |
| 2 | NN | Three | 0.16 | 0.19 | 0.13 | 0.17 | 0 |
| 2 | NN | Four | 0.13 | 0.17 | 0.10 | 0.14 | 0 |
| 2 | NN | Five | 0.09 | 0.13 | 0.06 | 0.10 | 0 |
| 2 | NN | Six | 0.04 | 0.06 | 0.02 | 0.04 | 0 |
| 2 | NN | Seven | 0.02 | 0.03 | 0.01 | 0.02 | 0 |
| 2 | NN | Eight | 0.01 | 0.02 | 0.01 | 0.02 | 0 |
| 2 | NN | Nine | 0.00 | 0.01 | 0.00 | 0.00 | 0 |
| 2 | NN | Ten | 0.00 | 0.01 | 0.00 | 0.00 | 0 |
| 2 | SPI | Zero | 0.13 | 0.21 | 0.07 | 0.15 | 0 |
| 2 | SPI | One | 0.13 | 0.18 | 0.09 | 0.15 | 0 |
| 2 | SPI | Two | 0.20 | 0.24 | 0.16 | 0.22 | 0 |
| 2 | SPI | Three | 0.17 | 0.20 | 0.14 | 0.18 | 0 |
| 2 | SPI | Four | 0.15 | 0.19 | 0.11 | 0.16 | 0 |
| 2 | SPI | Five | 0.11 | 0.15 | 0.07 | 0.12 | 0 |
| 2 | SPI | Six | 0.05 | 0.08 | 0.03 | 0.06 | 0 |
| 2 | SPI | Seven | 0.02 | 0.04 | 0.01 | 0.03 | 0 |
| 2 | SPI | Eight | 0.02 | 0.03 | 0.01 | 0.02 | 0 |
| 2 | SPI | Nine | 0.00 | 0.01 | 0.00 | 0.01 | 0 |
| 2 | SPI | Ten | 0.00 | 0.01 | 0.00 | 0.00 | 0 |

| | | | | | | | |
|---|---|---|---|---|---|---|---|
| 2 | EPI | Zero | 0.19 | 0.28 | 0.11 | 0.21 | 0 |
| 2 | EPI | One | 0.17 | 0.21 | 0.12 | 0.18 | 0 |
| 2 | EPI | Two | 0.21 | 0.24 | 0.18 | 0.22 | 0 |
| 2 | EPI | Three | 0.16 | 0.19 | 0.12 | 0.17 | 0 |
| 2 | EPI | Four | 0.12 | 0.16 | 0.08 | 0.14 | 0 |
| 2 | EPI | Five | 0.08 | 0.12 | 0.05 | 0.09 | 0 |
| 2 | EPI | Six | 0.03 | 0.06 | 0.02 | 0.04 | 0 |
| 2 | EPI | Seven | 0.02 | 0.03 | 0.01 | 0.02 | 0 |
| 2 | EPI | Eight | 0.01 | 0.02 | 0.01 | 0.01 | 0 |
| 2 | EPI | Nine | 0.00 | 0.01 | 0.00 | 0.00 | 0 |
| 2 | EPI | Ten | 0.00 | 0.00 | 0.00 | 0.00 | 0 |
| 3 | NN | Zero | 0.30 | 0.44 | 0.17 | 0.35 | 0 |
| 3 | NN | One | 0.20 | 0.24 | 0.16 | 0.22 | 0 |
| 3 | NN | Two | 0.20 | 0.24 | 0.16 | 0.22 | 0 |
| 3 | NN | Three | 0.12 | 0.16 | 0.08 | 0.14 | 0 |
| 3 | NN | Four | 0.08 | 0.12 | 0.04 | 0.09 | 0 |
| 3 | NN | Five | 0.05 | 0.08 | 0.02 | 0.05 | 0 |
| 3 | NN | Six | 0.02 | 0.03 | 0.01 | 0.02 | 0 |
| 3 | NN | Seven | 0.01 | 0.02 | 0.00 | 0.01 | 0 |
| 3 | NN | Eight | 0.01 | 0.01 | 0.00 | 0.01 | 0 |
| 3 | NN | Nine | 0.00 | 0.00 | 0.00 | 0.00 | 0 |
| 3 | NN | Ten | 0.00 | 0.00 | 0.00 | 0.00 | 0 |
| 3 | SPI | Zero | 0.17 | 0.29 | 0.09 | 0.20 | 0 |
| 3 | SPI | One | 0.16 | 0.21 | 0.10 | 0.18 | 0 |
| 3 | SPI | Two | 0.21 | 0.24 | 0.17 | 0.22 | 0 |
| 3 | SPI | Three | 0.16 | 0.19 | 0.12 | 0.18 | 0 |
| 3 | SPI | Four | 0.13 | 0.18 | 0.08 | 0.15 | 0 |
| 3 | SPI | Five | 0.08 | 0.14 | 0.04 | 0.10 | 0 |
| 3 | SPI | Six | 0.04 | 0.07 | 0.02 | 0.04 | 0 |
| 3 | SPI | Seven | 0.02 | 0.03 | 0.01 | 0.02 | 0 |
| 3 | SPI | Eight | 0.01 | 0.03 | 0.01 | 0.02 | 0 |
| 3 | SPI | Nine | 0.00 | 0.01 | 0.00 | 0.00 | 0 |
| 3 | SPI | Ten | 0.00 | 0.01 | 0.00 | 0.00 | 0 |
| 3 | EPI | Zero | 0.23 | 0.37 | 0.12 | 0.27 | 0 |
| 3 | EPI | One | 0.18 | 0.23 | 0.13 | 0.21 | 0 |
| 3 | EPI | Two | 0.21 | 0.24 | 0.18 | 0.23 | 0 |
| 3 | EPI | Three | 0.15 | 0.18 | 0.09 | 0.16 | 0 |
| 3 | EPI | Four | 0.10 | 0.16 | 0.06 | 0.12 | 0 |

| | | | | | | | |
|---|---|---|---|---|---|---|---|
| 3 | EPI | Five | 0.07 | 0.11 | 0.03 | 0.08 | 0 |
| 3 | EPI | Six | 0.03 | 0.05 | 0.01 | 0.03 | 0 |
| 3 | EPI | Seven | 0.01 | 0.03 | 0.00 | 0.02 | 0 |
| 3 | EPI | Eight | 0.01 | 0.02 | 0.00 | 0.01 | 0 |
| 3 | EPI | Nine | 0.00 | 0.01 | 0.00 | 0.00 | 0 |
| 3 | EPI | Ten | 0.00 | 0.00 | 0.00 | 0.00 | 0 |

# 4.2 Probability of adequate pain relief

We define adequate pain relief as a pain score of 4 or less.
Hence, PS of 5 or more are considered poor resting pain control.

## 4.2.1 Probability of poor pain control at rest

We determine the probability of poor resting pain control if one were to have a different analgesic technique.
The plot shows the posterior predictive distribution of the probability of poor pain control of the original analgesic technique, the posterior predictive distribution of the probability of poor pain control if one were to have a different analgesic technique, and the distribution of the differences in the probability of poor pain control (grey). The black error bars reflect the 95% credible intervals.

**Figure 8**

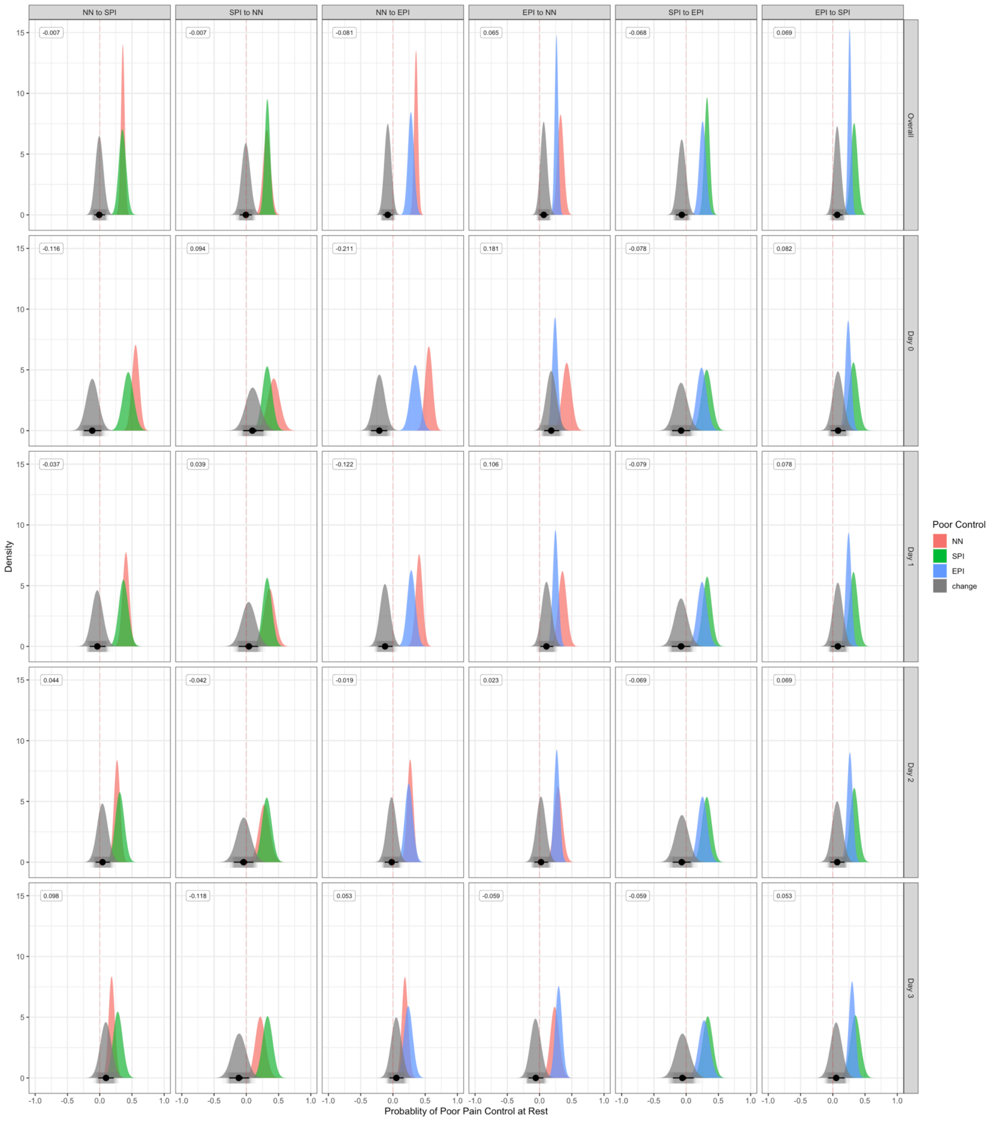


## Table 4

| AnalgesicTechniques | Day | Analg | ProbPoorControl.median | CI95low | CI95high |
|---|---|---|---|---|---|
| NN to SPI | Overall | NN | 0.36 | 0.31 | 0.40 |
| NN to SPI | Overall | SPI | 0.35 | 0.27 | 0.43 |
| NN to SPI | Overall | change | -0.01 | -0.09 | 0.08 |
| NN to SPI | Day 0 | NN | 0.56 | 0.48 | 0.63 |
| NN to SPI | Day 0 | SPI | 0.44 | 0.33 | 0.56 |
| NN to SPI | Day 0 | change | -0.12 | -0.26 | 0.01 |
| NN to SPI | Day 1 | NN | 0.41 | 0.32 | 0.47 |
| NN to SPI | Day 1 | SPI | 0.37 | 0.27 | 0.47 |
| NN to SPI | Day 1 | change | -0.04 | -0.16 | 0.09 |
| NN to SPI | Day 2 | NN | 0.27 | 0.20 | 0.33 |
| NN to SPI | Day 2 | SPI | 0.31 | 0.22 | 0.41 |
| NN to SPI | Day 2 | change | 0.04 | -0.06 | 0.16 |

| | | | | | |
|---|---|---|---|---|---|
| NN to SPI | Day 3 | NN | 0.19 | 0.13 | 0.26 |
| NN to SPI | Day 3 | SPI | 0.28 | 0.18 | 0.38 |
| NN to SPI | Day 3 | change | 0.10 | -0.02 | 0.21 |
| SPI to NN | Overall | NN | 0.32 | 0.25 | 0.40 |
| SPI to NN | Overall | SPI | 0.32 | 0.27 | 0.38 |
| SPI to NN | Overall | change | -0.01 | -0.11 | 0.08 |
| SPI to NN | Day 0 | NN | 0.42 | 0.31 | 0.56 |
| SPI to NN | Day 0 | SPI | 0.33 | 0.22 | 0.42 |
| SPI to NN | Day 0 | change | 0.09 | -0.05 | 0.25 |
| SPI to NN | Day 1 | NN | 0.36 | 0.25 | 0.47 |
| SPI to NN | Day 1 | SPI | 0.33 | 0.22 | 0.42 |
| SPI to NN | Day 1 | change | 0.04 | -0.13 | 0.17 |
| SPI to NN | Day 2 | NN | 0.28 | 0.17 | 0.39 |
| SPI to NN | Day 2 | SPI | 0.32 | 0.24 | 0.44 |
| SPI to NN | Day 2 | change | -0.04 | -0.18 | 0.11 |
| SPI to NN | Day 3 | NN | 0.22 | 0.10 | 0.32 |
| SPI to NN | Day 3 | SPI | 0.32 | 0.22 | 0.43 |
| SPI to NN | Day 3 | change | -0.12 | -0.26 | 0.03 |
| NN to EPI | Overall | NN | 0.36 | 0.32 | 0.40 |
| NN to EPI | Overall | EPI | 0.28 | 0.21 | 0.34 |
| NN to EPI | Overall | change | -0.08 | -0.16 | -0.01 |
| NN to EPI | Day 0 | NN | 0.56 | 0.47 | 0.63 |
| NN to EPI | Day 0 | EPI | 0.35 | 0.23 | 0.44 |
| NN to EPI | Day 0 | change | -0.21 | -0.35 | -0.10 |
| NN to EPI | Day 1 | NN | 0.41 | 0.34 | 0.48 |
| NN to EPI | Day 1 | EPI | 0.28 | 0.19 | 0.36 |
| NN to EPI | Day 1 | change | -0.12 | -0.23 | -0.01 |
| NN to EPI | Day 2 | NN | 0.27 | 0.20 | 0.33 |
| NN to EPI | Day 2 | EPI | 0.25 | 0.16 | 0.33 |
| NN to EPI | Day 2 | change | -0.02 | -0.13 | 0.08 |
| NN to EPI | Day 3 | NN | 0.19 | 0.12 | 0.25 |
| NN to EPI | Day 3 | EPI | 0.24 | 0.15 | 0.33 |
| NN to EPI | Day 3 | change | 0.05 | -0.06 | 0.16 |
| EPI to NN | Overall | NN | 0.33 | 0.26 | 0.40 |
| EPI to NN | Overall | EPI | 0.26 | 0.23 | 0.30 |
| EPI to NN | Overall | change | 0.06 | 0.00 | 0.14 |
| EPI to NN | Day 0 | NN | 0.42 | 0.32 | 0.52 |
| EPI to NN | Day 0 | EPI | 0.24 | 0.19 | 0.30 |

| | | | | | |
|---|---|---|---|---|---|
| EPI to NN | Day 0 | change | 0.18 | 0.06 | 0.29 |
| EPI to NN | Day 1 | NN | 0.36 | 0.27 | 0.44 |
| EPI to NN | Day 1 | EPI | 0.25 | 0.18 | 0.30 |
| EPI to NN | Day 1 | change | 0.11 | 0.01 | 0.21 |
| EPI to NN | Day 2 | NN | 0.29 | 0.21 | 0.38 |
| EPI to NN | Day 2 | EPI | 0.27 | 0.21 | 0.33 |
| EPI to NN | Day 2 | change | 0.02 | -0.09 | 0.13 |
| EPI to NN | Day 3 | NN | 0.23 | 0.15 | 0.34 |
| EPI to NN | Day 3 | EPI | 0.29 | 0.22 | 0.36 |
| EPI to NN | Day 3 | change | -0.06 | -0.17 | 0.06 |
| SPI to EPI | Overall | SPI | 0.32 | 0.26 | 0.38 |
| SPI to EPI | Overall | EPI | 0.25 | 0.19 | 0.33 |
| SPI to EPI | Overall | change | -0.07 | -0.16 | 0.02 |
| SPI to EPI | Day 0 | SPI | 0.31 | 0.20 | 0.42 |
| SPI to EPI | Day 0 | EPI | 0.23 | 0.12 | 0.33 |
| SPI to EPI | Day 0 | change | -0.08 | -0.23 | 0.05 |
| SPI to EPI | Day 1 | SPI | 0.33 | 0.22 | 0.42 |
| SPI to EPI | Day 1 | EPI | 0.25 | 0.16 | 0.36 |
| SPI to EPI | Day 1 | change | -0.08 | -0.22 | 0.05 |
| SPI to EPI | Day 2 | SPI | 0.32 | 0.22 | 0.42 |
| SPI to EPI | Day 2 | EPI | 0.25 | 0.15 | 0.35 |
| SPI to EPI | Day 2 | change | -0.07 | -0.21 | 0.07 |
| SPI to EPI | Day 3 | SPI | 0.34 | 0.21 | 0.43 |
| SPI to EPI | Day 3 | EPI | 0.28 | 0.16 | 0.38 |
| SPI to EPI | Day 3 | change | -0.06 | -0.22 | 0.09 |
| EPI to SPI | Overall | SPI | 0.33 | 0.26 | 0.41 |
| EPI to SPI | Overall | EPI | 0.26 | 0.23 | 0.30 |
| EPI to SPI | Overall | change | 0.07 | -0.01 | 0.15 |
| EPI to SPI | Day 0 | SPI | 0.32 | 0.22 | 0.42 |
| EPI to SPI | Day 0 | EPI | 0.24 | 0.17 | 0.29 |
| EPI to SPI | Day 0 | change | 0.08 | -0.04 | 0.19 |
| EPI to SPI | Day 1 | SPI | 0.32 | 0.23 | 0.41 |
| EPI to SPI | Day 1 | EPI | 0.25 | 0.18 | 0.30 |
| EPI to SPI | Day 1 | change | 0.08 | -0.03 | 0.18 |
| EPI to SPI | Day 2 | SPI | 0.33 | 0.25 | 0.43 |
| EPI to SPI | Day 2 | EPI | 0.26 | 0.21 | 0.33 |
| EPI to SPI | Day 2 | change | 0.07 | -0.04 | 0.18 |
| EPI to SPI | Day 3 | SPI | 0.35 | 0.25 | 0.46 |

| | | | | | |
|---|---|---|---|---|---|
| EPI to SPI | Day 3 | EPI | 0.30 | 0.23 | 0.36 |
| EPI to SPI | Day 3 | change | 0.05 | -0.07 | 0.18 |

## 4.2.2 by Surgical Approach

### Figure 9

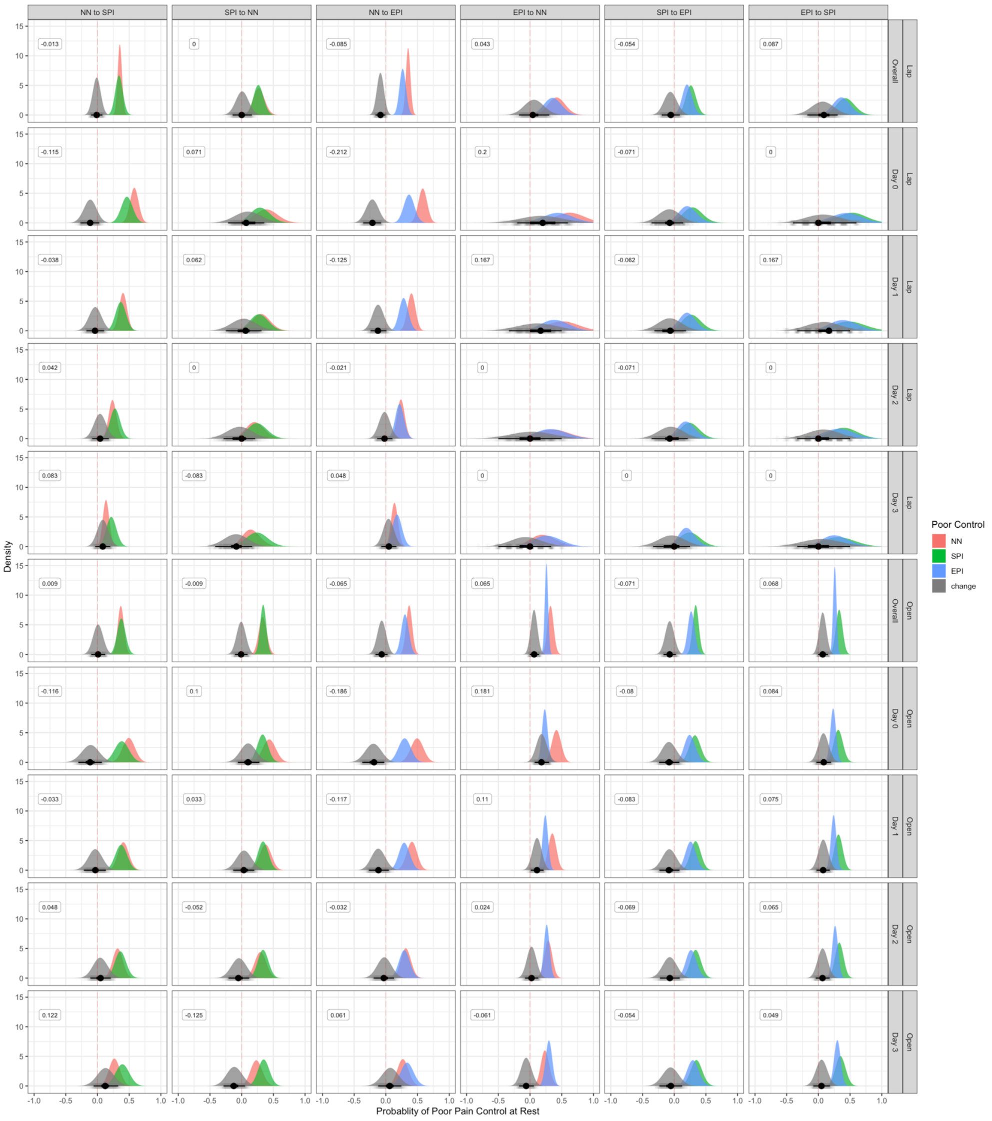


### Table 5

| SurgAppr | AnalgesicTechniques | Day | Analg | ProbPoorControl.median | CI95low | CI95 |
|---|---|---|---|---|---|---|
| Lap | NN to SPI | Overall | NN | 0.35 | 0.31 | |
| Lap | NN to SPI | Overall | SPI | 0.34 | 0.26 | |
| Lap | NN to SPI | Overall | change | -0.01 | -0.10 | |
| Lap | NN to SPI | Day 0 | NN | 0.59 | 0.50 | |

| | | | | | |
|---|---|---|---|---|---|
| Lap | NN to SPI | Day 0 | SPI | 0.46 | 0.34 |
| Lap | NN to SPI | Day 0 | change | -0.12 | -0.26 |
| Lap | NN to SPI | Day 1 | NN | 0.40 | 0.32 |
| Lap | NN to SPI | Day 1 | SPI | 0.36 | 0.26 |
| Lap | NN to SPI | Day 1 | change | -0.04 | -0.19 |
| Lap | NN to SPI | Day 2 | NN | 0.24 | 0.15 |
| Lap | NN to SPI | Day 2 | SPI | 0.28 | 0.18 |
| Lap | NN to SPI | Day 2 | change | 0.04 | -0.09 |
| Lap | NN to SPI | Day 3 | NN | 0.13 | 0.06 |
| Lap | NN to SPI | Day 3 | SPI | 0.21 | 0.12 |
| Lap | NN to SPI | Day 3 | change | 0.08 | -0.04 |
| Lap | SPI to NN | Overall | NN | 0.27 | 0.16 |
| Lap | SPI to NN | Overall | SPI | 0.27 | 0.16 |
| Lap | SPI to NN | Overall | change | 0.00 | -0.14 |
| Lap | SPI to NN | Day 0 | NN | 0.36 | 0.21 |
| Lap | SPI to NN | Day 0 | SPI | 0.29 | 0.14 |
| Lap | SPI to NN | Day 0 | change | 0.07 | -0.14 |
| Lap | SPI to NN | Day 1 | NN | 0.31 | 0.12 |
| Lap | SPI to NN | Day 1 | SPI | 0.25 | 0.06 |
| Lap | SPI to NN | Day 1 | change | 0.06 | -0.25 |
| Lap | SPI to NN | Day 2 | NN | 0.21 | 0.07 |
| Lap | SPI to NN | Day 2 | SPI | 0.21 | 0.07 |
| Lap | SPI to NN | Day 2 | change | 0.00 | -0.29 |
| Lap | SPI to NN | Day 3 | NN | 0.17 | 0.00 |
| Lap | SPI to NN | Day 3 | SPI | 0.25 | 0.00 |
| Lap | SPI to NN | Day 3 | change | -0.08 | -0.33 |
| Lap | NN to EPI | Overall | NN | 0.35 | 0.31 |
| Lap | NN to EPI | Overall | EPI | 0.26 | 0.20 |
| Lap | NN to EPI | Overall | change | -0.09 | -0.16 |
| Lap | NN to EPI | Day 0 | NN | 0.58 | 0.49 |
| Lap | NN to EPI | Day 0 | EPI | 0.36 | 0.23 |
| Lap | NN to EPI | Day 0 | change | -0.21 | -0.35 |
| Lap | NN to EPI | Day 1 | NN | 0.40 | 0.31 |
| Lap | NN to EPI | Day 1 | EPI | 0.28 | 0.17 |
| Lap | NN to EPI | Day 1 | change | -0.12 | -0.25 |
| Lap | NN to EPI | Day 2 | NN | 0.24 | 0.15 |
| Lap | NN to EPI | Day 2 | EPI | 0.22 | 0.12 |
| Lap | NN to EPI | Day 2 | change | -0.02 | -0.15 |


1 The Data
2 Goals of the analysis
3 The Model
4 Posterior Distributions of Pain Scores at Rest
4.1 Adjusted resting pain scores
4.2 Probability of adequate pain relief
4.2.1 Probability of poor pain control at rest
4.2.2 by Surgical Approach
4.2.3 by Surgical Type
5 Software and packages used

| | | | | | |
|---|---|---|---|---|---|
| Lap | NN to EPI | Day 3 | NN | 0.13 | 0.06 |
| Lap | NN to EPI | Day 3 | EPI | 0.18 | 0.10 |
| Lap | NN to EPI | Day 3 | change | 0.05 | -0.06 |
| Lap | EPI to NN | Overall | NN | 0.44 | 0.22 |
| Lap | EPI to NN | Overall | EPI | 0.35 | 0.17 |
| Lap | EPI to NN | Overall | change | 0.04 | -0.13 |
| Lap | EPI to NN | Day 0 | NN | 0.60 | 0.40 |
| Lap | EPI to NN | Day 0 | EPI | 0.40 | 0.20 |
| Lap | EPI to NN | Day 0 | change | 0.20 | -0.20 |
| Lap | EPI to NN | Day 1 | NN | 0.50 | 0.17 |
| Lap | EPI to NN | Day 1 | EPI | 0.33 | 0.17 |
| Lap | EPI to NN | Day 1 | change | 0.17 | -0.33 |
| Lap | EPI to NN | Day 2 | NN | 0.33 | 0.00 |
| Lap | EPI to NN | Day 2 | EPI | 0.33 | 0.00 |
| Lap | EPI to NN | Day 2 | change | 0.00 | -0.33 |
| Lap | EPI to NN | Day 3 | NN | 0.17 | 0.00 |
| Lap | EPI to NN | Day 3 | EPI | 0.33 | 0.00 |
| Lap | EPI to NN | Day 3 | change | 0.00 | -0.50 |
| Lap | SPI to EPI | Overall | SPI | 0.27 | 0.16 |
| Lap | SPI to EPI | Overall | EPI | 0.20 | 0.09 |
| Lap | SPI to EPI | Overall | change | -0.05 | -0.20 |
| Lap | SPI to EPI | Day 0 | SPI | 0.29 | 0.14 |
| Lap | SPI to EPI | Day 0 | EPI | 0.21 | 0.07 |
| Lap | SPI to EPI | Day 0 | change | -0.07 | -0.36 |
| Lap | SPI to EPI | Day 1 | SPI | 0.25 | 0.06 |
| Lap | SPI to EPI | Day 1 | EPI | 0.19 | 0.06 |
| Lap | SPI to EPI | Day 1 | change | -0.06 | -0.38 |
| Lap | SPI to EPI | Day 2 | SPI | 0.21 | 0.07 |
| Lap | SPI to EPI | Day 2 | EPI | 0.21 | 0.07 |
| Lap | SPI to EPI | Day 2 | change | -0.07 | -0.29 |
| Lap | SPI to EPI | Day 3 | SPI | 0.25 | 0.00 |
| Lap | SPI to EPI | Day 3 | EPI | 0.17 | 0.00 |
| Lap | SPI to EPI | Day 3 | change | 0.00 | -0.33 |
| Lap | EPI to SPI | Overall | SPI | 0.44 | 0.26 |
| Lap | EPI to SPI | Overall | EPI | 0.35 | 0.17 |
| Lap | EPI to SPI | Overall | change | 0.09 | -0.17 |
| Lap | EPI to SPI | Day 0 | SPI | 0.60 | 0.20 |
| Lap | EPI to SPI | Day 0 | EPI | 0.40 | 0.20 |

| | | | | | |
|---|---|---|---|---|---|
| Lap | EPI to SPI | Day 0 | change | 0.00 | -0.40 |
| Lap | EPI to SPI | Day 1 | SPI | 0.50 | 0.17 |
| Lap | EPI to SPI | Day 1 | EPI | 0.33 | 0.17 |
| Lap | EPI to SPI | Day 1 | change | 0.17 | -0.33 |
| Lap | EPI to SPI | Day 2 | SPI | 0.33 | 0.17 |
| Lap | EPI to SPI | Day 2 | EPI | 0.33 | 0.00 |
| Lap | EPI to SPI | Day 2 | change | 0.00 | -0.33 |
| Lap | EPI to SPI | Day 3 | SPI | 0.33 | 0.00 |
| Lap | EPI to SPI | Day 3 | EPI | 0.33 | 0.00 |
| Lap | EPI to SPI | Day 3 | change | 0.00 | -0.33 |
| Open | NN to SPI | Overall | NN | 0.37 | 0.30 |
| Open | NN to SPI | Overall | SPI | 0.38 | 0.28 |
| Open | NN to SPI | Overall | change | 0.01 | -0.10 |
| Open | NN to SPI | Day 0 | NN | 0.49 | 0.37 |
| Open | NN to SPI | Day 0 | SPI | 0.37 | 0.21 |
| Open | NN to SPI | Day 0 | change | -0.12 | -0.30 |
| Open | NN to SPI | Day 1 | NN | 0.42 | 0.30 |
| Open | NN to SPI | Day 1 | SPI | 0.37 | 0.25 |
| Open | NN to SPI | Day 1 | change | -0.03 | -0.22 |
| Open | NN to SPI | Day 2 | NN | 0.32 | 0.21 |
| Open | NN to SPI | Day 2 | SPI | 0.36 | 0.23 |
| Open | NN to SPI | Day 2 | change | 0.05 | -0.10 |
| Open | NN to SPI | Day 3 | NN | 0.26 | 0.16 |
| Open | NN to SPI | Day 3 | SPI | 0.39 | 0.24 |
| Open | NN to SPI | Day 3 | change | 0.12 | -0.06 |
| Open | SPI to NN | Overall | NN | 0.33 | 0.25 |
| Open | SPI to NN | Overall | SPI | 0.34 | 0.28 |
| Open | SPI to NN | Overall | change | -0.01 | -0.11 |
| Open | SPI to NN | Day 0 | NN | 0.44 | 0.30 |
| Open | SPI to NN | Day 0 | SPI | 0.34 | 0.20 |
| Open | SPI to NN | Day 0 | change | 0.10 | -0.06 |
| Open | SPI to NN | Day 1 | NN | 0.38 | 0.25 |
| Open | SPI to NN | Day 1 | SPI | 0.33 | 0.22 |
| Open | SPI to NN | Day 1 | change | 0.03 | -0.15 |
| Open | SPI to NN | Day 2 | NN | 0.29 | 0.17 |
| Open | SPI to NN | Day 2 | SPI | 0.34 | 0.24 |
| Open | SPI to NN | Day 2 | change | -0.05 | -0.21 |
| Open | SPI to NN | Day 3 | NN | 0.23 | 0.11 |

| | | | | | |
|---|---|---|---|---|---|
| Open | SPI to NN | Day 3 | SPI | 0.34 | 0.23 |
| Open | SPI to NN | Day 3 | change | -0.12 | -0.29 |
| Open | NN to EPI | Overall | NN | 0.37 | 0.30 |
| Open | NN to EPI | Overall | EPI | 0.30 | 0.22 |
| Open | NN to EPI | Overall | change | -0.06 | -0.18 |
| Open | NN to EPI | Day 0 | NN | 0.49 | 0.37 |
| Open | NN to EPI | Day 0 | EPI | 0.30 | 0.16 |
| Open | NN to EPI | Day 0 | change | -0.19 | -0.37 |
| Open | NN to EPI | Day 1 | NN | 0.42 | 0.30 |
| Open | NN to EPI | Day 1 | EPI | 0.28 | 0.17 |
| Open | NN to EPI | Day 1 | change | -0.12 | -0.28 |
| Open | NN to EPI | Day 2 | NN | 0.32 | 0.21 |
| Open | NN to EPI | Day 2 | EPI | 0.29 | 0.18 |
| Open | NN to EPI | Day 2 | change | -0.03 | -0.18 |
| Open | NN to EPI | Day 3 | NN | 0.26 | 0.14 |
| Open | NN to EPI | Day 3 | EPI | 0.33 | 0.18 |
| Open | NN to EPI | Day 3 | change | 0.06 | -0.14 |
| Open | EPI to NN | Overall | NN | 0.32 | 0.26 |
| Open | EPI to NN | Overall | EPI | 0.26 | 0.22 |
| Open | EPI to NN | Overall | change | 0.06 | -0.01 |
| Open | EPI to NN | Day 0 | NN | 0.42 | 0.32 |
| Open | EPI to NN | Day 0 | EPI | 0.23 | 0.17 |
| Open | EPI to NN | Day 0 | change | 0.18 | 0.07 |
| Open | EPI to NN | Day 1 | NN | 0.35 | 0.26 |
| Open | EPI to NN | Day 1 | EPI | 0.24 | 0.18 |
| Open | EPI to NN | Day 1 | change | 0.11 | 0.01 |
| Open | EPI to NN | Day 2 | NN | 0.28 | 0.20 |
| Open | EPI to NN | Day 2 | EPI | 0.26 | 0.20 |
| Open | EPI to NN | Day 2 | change | 0.02 | -0.08 |
| Open | EPI to NN | Day 3 | NN | 0.24 | 0.15 |
| Open | EPI to NN | Day 3 | EPI | 0.30 | 0.21 |
| Open | EPI to NN | Day 3 | change | -0.06 | -0.18 |
| Open | SPI to EPI | Overall | SPI | 0.34 | 0.27 |
| Open | SPI to EPI | Overall | EPI | 0.27 | 0.19 |
| Open | SPI to EPI | Overall | change | -0.07 | -0.16 |
| Open | SPI to EPI | Day 0 | SPI | 0.32 | 0.20 |
| Open | SPI to EPI | Day 0 | EPI | 0.24 | 0.14 |

| | | | | | |
|---|---|---|---|---|---|
| Open | SPI to EPI | Day 0 | change | -0.08 | -0.26 |
| Open | SPI to EPI | Day 1 | SPI | 0.33 | 0.23 |
| Open | SPI to EPI | Day 1 | EPI | 0.27 | 0.15 |
| Open | SPI to EPI | Day 1 | change | -0.08 | -0.23 |
| Open | SPI to EPI | Day 2 | SPI | 0.34 | 0.24 |
| Open | SPI to EPI | Day 2 | EPI | 0.26 | 0.16 |
| Open | SPI to EPI | Day 2 | change | -0.07 | -0.22 |
| Open | SPI to EPI | Day 3 | SPI | 0.36 | 0.21 |
| Open | SPI to EPI | Day 3 | EPI | 0.29 | 0.16 |
| Open | SPI to EPI | Day 3 | change | -0.05 | -0.23 |
| Open | EPI to SPI | Overall | SPI | 0.33 | 0.26 |
| Open | EPI to SPI | Overall | EPI | 0.26 | 0.22 |
| Open | EPI to SPI | Overall | change | 0.07 | 0.00 |
| Open | EPI to SPI | Day 0 | SPI | 0.31 | 0.21 |
| Open | EPI to SPI | Day 0 | EPI | 0.23 | 0.17 |
| Open | EPI to SPI | Day 0 | change | 0.08 | -0.03 |
| Open | EPI to SPI | Day 1 | SPI | 0.32 | 0.22 |
| Open | EPI to SPI | Day 1 | EPI | 0.24 | 0.17 |
| Open | EPI to SPI | Day 1 | change | 0.07 | -0.04 |
| Open | EPI to SPI | Day 2 | SPI | 0.33 | 0.24 |
| Open | EPI to SPI | Day 2 | EPI | 0.26 | 0.20 |
| Open | EPI to SPI | Day 2 | change | 0.06 | -0.04 |
| Open | EPI to SPI | Day 3 | SPI | 0.35 | 0.24 |
| Open | EPI to SPI | Day 3 | EPI | 0.30 | 0.23 |
| Open | EPI to SPI | Day 3 | change | 0.05 | -0.07 |

### 4.2.3 by Surgical Type

**Figure 10**

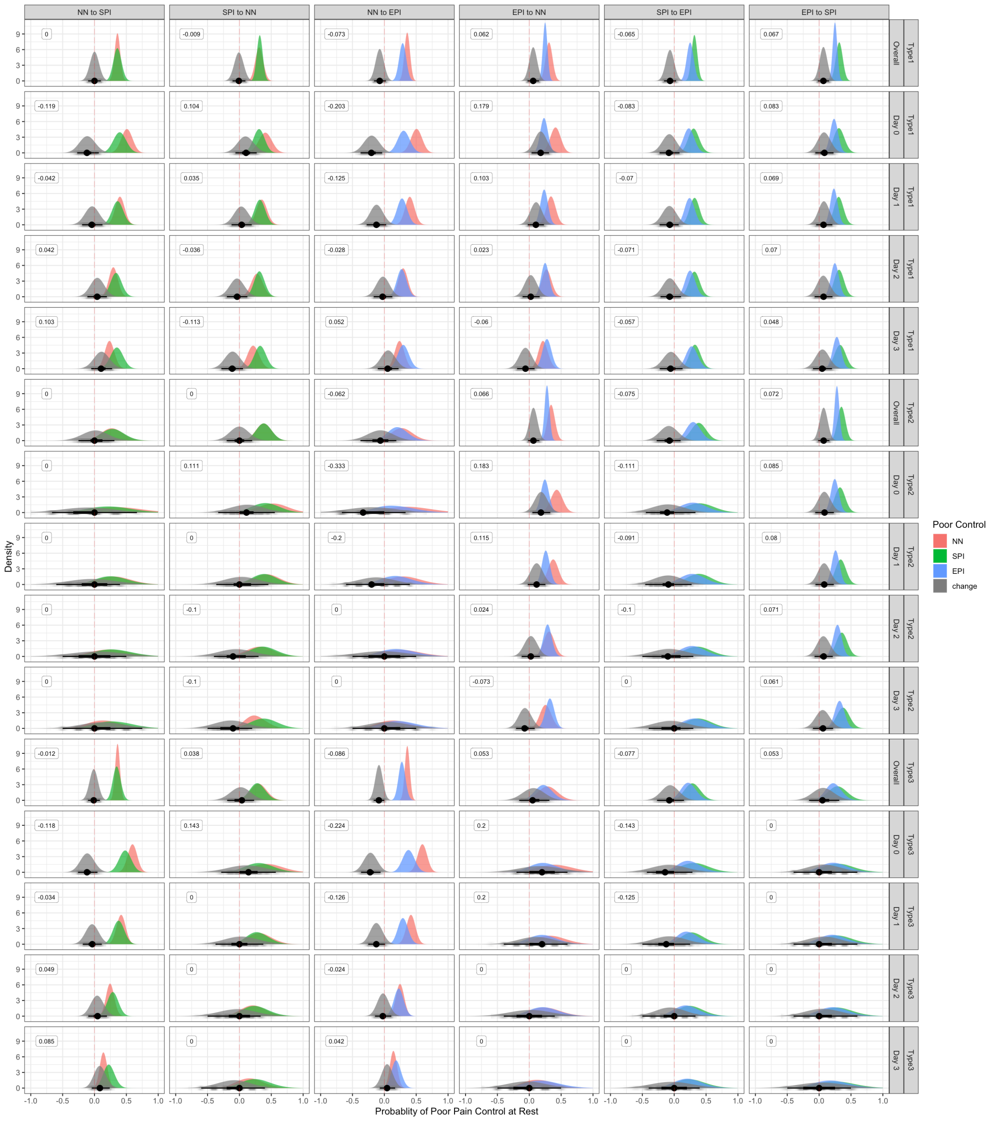


## Table 6

| SurgType | AnalgesicTechniques | Day | Analg | ProbPoorControl.median | CI95low | CI95[illegible] |
|---|---|---|---|---|---|---|
| Type1 | NN to SPI | Overall | NN | 0.36 | 0.30 | |
| Type1 | NN to SPI | Overall | SPI | 0.36 | 0.28 | |
| Type1 | NN to SPI | Overall | change | 0.00 | -0.10 | |
| Type1 | NN to SPI | Day 0 | NN | 0.51 | 0.41 | |
| Type1 | NN to SPI | Day 0 | SPI | 0.39 | 0.27 | |
| Type1 | NN to SPI | Day 0 | change | -0.12 | -0.27 | |
| Type1 | NN to SPI | Day 1 | NN | 0.40 | 0.31 | |
| Type1 | NN to SPI | Day 1 | SPI | 0.36 | 0.25 | |
| Type1 | NN to SPI | Day 1 | change | -0.04 | -0.19 | |
| Type1 | NN to SPI | Day 2 | NN | 0.29 | 0.19 | |
| Type1 | NN to SPI | Day 2 | SPI | 0.33 | 0.22 | |
| Type1 | NN to SPI | Day 2 | change | 0.04 | -0.11 | |

| | | | | | |
|---|---|---|---|---|---|
| Type1 | NN to SPI | Day 3 | NN | 0.24 | 0.14 |
| Type1 | NN to SPI | Day 3 | SPI | 0.34 | 0.21 |
| Type1 | NN to SPI | Day 3 | change | 0.10 | -0.05 |
| Type1 | SPI to NN | Overall | NN | 0.31 | 0.22 |
| Type1 | SPI to NN | Overall | SPI | 0.32 | 0.24 |
| Type1 | SPI to NN | Overall | change | -0.01 | -0.11 |
| Type1 | SPI to NN | Day 0 | NN | 0.40 | 0.27 |
| Type1 | SPI to NN | Day 0 | SPI | 0.31 | 0.19 |
| Type1 | SPI to NN | Day 0 | change | 0.10 | -0.06 |
| Type1 | SPI to NN | Day 1 | NN | 0.35 | 0.21 |
| Type1 | SPI to NN | Day 1 | SPI | 0.32 | 0.21 |
| Type1 | SPI to NN | Day 1 | change | 0.04 | -0.12 |
| Type1 | SPI to NN | Day 2 | NN | 0.27 | 0.16 |
| Type1 | SPI to NN | Day 2 | SPI | 0.32 | 0.21 |
| Type1 | SPI to NN | Day 2 | change | -0.04 | -0.20 |
| Type1 | SPI to NN | Day 3 | NN | 0.21 | 0.11 |
| Type1 | SPI to NN | Day 3 | SPI | 0.32 | 0.19 |
| Type1 | SPI to NN | Day 3 | change | -0.11 | -0.30 |
| Type1 | NN to EPI | Overall | NN | 0.36 | 0.30 |
| Type1 | NN to EPI | Overall | EPI | 0.29 | 0.22 |
| Type1 | NN to EPI | Overall | change | -0.07 | -0.16 |
| Type1 | NN to EPI | Day 0 | NN | 0.51 | 0.41 |
| Type1 | NN to EPI | Day 0 | EPI | 0.30 | 0.17 |
| Type1 | NN to EPI | Day 0 | change | -0.20 | -0.37 |
| Type1 | NN to EPI | Day 1 | NN | 0.40 | 0.31 |
| Type1 | NN to EPI | Day 1 | EPI | 0.28 | 0.17 |
| Type1 | NN to EPI | Day 1 | change | -0.12 | -0.28 |
| Type1 | NN to EPI | Day 2 | NN | 0.29 | 0.19 |
| Type1 | NN to EPI | Day 2 | EPI | 0.28 | 0.15 |
| Type1 | NN to EPI | Day 2 | change | -0.03 | -0.18 |
| Type1 | NN to EPI | Day 3 | NN | 0.24 | 0.14 |
| Type1 | NN to EPI | Day 3 | EPI | 0.29 | 0.17 |
| Type1 | NN to EPI | Day 3 | change | 0.05 | -0.09 |
| Type1 | EPI to NN | Overall | NN | 0.31 | 0.24 |
| Type1 | EPI to NN | Overall | EPI | 0.25 | 0.20 |
| Type1 | EPI to NN | Overall | change | 0.06 | -0.03 |
| Type1 | EPI to NN | Day 0 | NN | 0.42 | 0.30 |
| Type1 | EPI to NN | Day 0 | EPI | 0.24 | 0.16 |

| | | | | | |
|---|---|---|---|---|---|
| Type1 | EPI to NN | Day 0 | change | 0.18 | 0.05 |
| Type1 | EPI to NN | Day 1 | NN | 0.34 | 0.24 |
| Type1 | EPI to NN | Day 1 | EPI | 0.23 | 0.15 |
| Type1 | EPI to NN | Day 1 | change | 0.10 | -0.02 |
| Type1 | EPI to NN | Day 2 | NN | 0.27 | 0.16 |
| Type1 | EPI to NN | Day 2 | EPI | 0.24 | 0.16 |
| Type1 | EPI to NN | Day 2 | change | 0.02 | -0.10 |
| Type1 | EPI to NN | Day 3 | NN | 0.21 | 0.12 |
| Type1 | EPI to NN | Day 3 | EPI | 0.27 | 0.19 |
| Type1 | EPI to NN | Day 3 | change | -0.06 | -0.20 |
| Type1 | SPI to EPI | Overall | SPI | 0.32 | 0.26 |
| Type1 | SPI to EPI | Overall | EPI | 0.25 | 0.17 |
| Type1 | SPI to EPI | Overall | change | -0.06 | -0.16 |
| Type1 | SPI to EPI | Day 0 | SPI | 0.31 | 0.19 |
| Type1 | SPI to EPI | Day 0 | EPI | 0.23 | 0.10 |
| Type1 | SPI to EPI | Day 0 | change | -0.08 | -0.25 |
| Type1 | SPI to EPI | Day 1 | SPI | 0.32 | 0.21 |
| Type1 | SPI to EPI | Day 1 | EPI | 0.25 | 0.14 |
| Type1 | SPI to EPI | Day 1 | change | -0.07 | -0.25 |
| Type1 | SPI to EPI | Day 2 | SPI | 0.32 | 0.21 |
| Type1 | SPI to EPI | Day 2 | EPI | 0.25 | 0.12 |
| Type1 | SPI to EPI | Day 2 | change | -0.07 | -0.21 |
| Type1 | SPI to EPI | Day 3 | SPI | 0.32 | 0.21 |
| Type1 | SPI to EPI | Day 3 | EPI | 0.26 | 0.15 |
| Type1 | SPI to EPI | Day 3 | change | -0.06 | -0.23 |
| Type1 | EPI to SPI | Overall | SPI | 0.32 | 0.25 |
| Type1 | EPI to SPI | Overall | EPI | 0.25 | 0.20 |
| Type1 | EPI to SPI | Overall | change | 0.07 | -0.02 |
| Type1 | EPI to SPI | Day 0 | SPI | 0.31 | 0.20 |
| Type1 | EPI to SPI | Day 0 | EPI | 0.24 | 0.14 |
| Type1 | EPI to SPI | Day 0 | change | 0.08 | -0.07 |
| Type1 | EPI to SPI | Day 1 | SPI | 0.31 | 0.21 |
| Type1 | EPI to SPI | Day 1 | EPI | 0.23 | 0.16 |
| Type1 | EPI to SPI | Day 1 | change | 0.07 | -0.07 |
| Type1 | EPI to SPI | Day 2 | SPI | 0.31 | 0.21 |
| Type1 | EPI to SPI | Day 2 | EPI | 0.24 | 0.16 |
| Type1 | EPI to SPI | Day 2 | change | 0.07 | -0.07 |
| Type1 | EPI to SPI | Day 3 | SPI | 0.33 | 0.21 |

| | | | | | |
|---|---|---|---|---|---|
| Type1 | EPI to SPI | Day 3 | EPI | 0.27 | 0.19 |
| Type1 | EPI to SPI | Day 3 | change | 0.05 | -0.11 |
| Type2 | NN to SPI | Overall | NN | 0.25 | 0.06 |
| Type2 | NN to SPI | Overall | SPI | 0.31 | 0.06 |
| Type2 | NN to SPI | Overall | change | 0.00 | -0.31 |
| Type2 | NN to SPI | Day 0 | NN | 0.33 | 0.00 |
| Type2 | NN to SPI | Day 0 | SPI | 0.33 | 0.00 |
| Type2 | NN to SPI | Day 0 | change | 0.00 | -0.67 |
| Type2 | NN to SPI | Day 1 | NN | 0.40 | 0.00 |
| Type2 | NN to SPI | Day 1 | SPI | 0.20 | 0.00 |
| Type2 | NN to SPI | Day 1 | change | 0.00 | -0.60 |
| Type2 | NN to SPI | Day 2 | NN | 0.25 | 0.00 |
| Type2 | NN to SPI | Day 2 | SPI | 0.25 | 0.00 |
| Type2 | NN to SPI | Day 2 | change | 0.00 | -0.50 |
| Type2 | NN to SPI | Day 3 | NN | 0.25 | 0.00 |
| Type2 | NN to SPI | Day 3 | SPI | 0.25 | 0.00 |
| Type2 | NN to SPI | Day 3 | change | 0.00 | -0.50 |
| Type2 | SPI to NN | Overall | NN | 0.38 | 0.22 |
| Type2 | SPI to NN | Overall | SPI | 0.38 | 0.25 |
| Type2 | SPI to NN | Overall | change | 0.00 | -0.20 |
| Type2 | SPI to NN | Day 0 | NN | 0.56 | 0.22 |
| Type2 | SPI to NN | Day 0 | SPI | 0.44 | 0.11 |
| Type2 | SPI to NN | Day 0 | change | 0.11 | -0.33 |
| Type2 | SPI to NN | Day 1 | NN | 0.46 | 0.18 |
| Type2 | SPI to NN | Day 1 | SPI | 0.36 | 0.09 |
| Type2 | SPI to NN | Day 1 | change | 0.00 | -0.36 |
| Type2 | SPI to NN | Day 2 | NN | 0.30 | 0.10 |
| Type2 | SPI to NN | Day 2 | SPI | 0.40 | 0.10 |
| Type2 | SPI to NN | Day 2 | change | -0.10 | -0.40 |
| Type2 | SPI to NN | Day 3 | NN | 0.20 | 0.00 |
| Type2 | SPI to NN | Day 3 | SPI | 0.40 | 0.10 |
| Type2 | SPI to NN | Day 3 | change | -0.10 | -0.50 |
| Type2 | NN to EPI | Overall | NN | 0.25 | 0.06 |
| Type2 | NN to EPI | Overall | EPI | 0.19 | 0.06 |
| Type2 | NN to EPI | Overall | change | -0.06 | -0.31 |
| Type2 | NN to EPI | Day 0 | NN | 0.33 | 0.00 |
| Type2 | NN to EPI | Day 0 | EPI | 0.00 | 0.00 |
| Type2 | NN to EPI | Day 0 | change | -0.33 | -0.67 |

| | | | | | |
|---|---|---|---|---|---|
| Type2 | NN to EPI | Day 1 | NN | 0.40 | 0.00 |
| Type2 | NN to EPI | Day 1 | EPI | 0.20 | 0.00 |
| Type2 | NN to EPI | Day 1 | change | -0.20 | -0.80 |
| Type2 | NN to EPI | Day 2 | NN | 0.25 | 0.00 |
| Type2 | NN to EPI | Day 2 | EPI | 0.25 | 0.00 |
| Type2 | NN to EPI | Day 2 | change | 0.00 | -0.50 |
| Type2 | NN to EPI | Day 3 | NN | 0.25 | 0.00 |
| Type2 | NN to EPI | Day 3 | EPI | 0.25 | 0.00 |
| Type2 | NN to EPI | Day 3 | change | 0.00 | -0.50 |
| Type2 | EPI to NN | Overall | NN | 0.34 | 0.27 |
| Type2 | EPI to NN | Overall | EPI | 0.28 | 0.22 |
| Type2 | EPI to NN | Overall | change | 0.07 | -0.02 |
| Type2 | EPI to NN | Day 0 | NN | 0.43 | 0.30 |
| Type2 | EPI to NN | Day 0 | EPI | 0.24 | 0.16 |
| Type2 | EPI to NN | Day 0 | change | 0.18 | 0.05 |
| Type2 | EPI to NN | Day 1 | NN | 0.38 | 0.25 |
| Type2 | EPI to NN | Day 1 | EPI | 0.26 | 0.16 |
| Type2 | EPI to NN | Day 1 | change | 0.12 | -0.02 |
| Type2 | EPI to NN | Day 2 | NN | 0.31 | 0.20 |
| Type2 | EPI to NN | Day 2 | EPI | 0.29 | 0.20 |
| Type2 | EPI to NN | Day 2 | change | 0.02 | -0.11 |
| Type2 | EPI to NN | Day 3 | NN | 0.26 | 0.12 |
| Type2 | EPI to NN | Day 3 | EPI | 0.33 | 0.21 |
| Type2 | EPI to NN | Day 3 | change | -0.07 | -0.21 |
| Type2 | SPI to EPI | Overall | SPI | 0.38 | 0.25 |
| Type2 | SPI to EPI | Overall | EPI | 0.30 | 0.15 |
| Type2 | SPI to EPI | Overall | change | -0.07 | -0.28 |
| Type2 | SPI to EPI | Day 0 | SPI | 0.44 | 0.11 |
| Type2 | SPI to EPI | Day 0 | EPI | 0.33 | 0.00 |
| Type2 | SPI to EPI | Day 0 | change | -0.11 | -0.44 |
| Type2 | SPI to EPI | Day 1 | SPI | 0.36 | 0.09 |
| Type2 | SPI to EPI | Day 1 | EPI | 0.27 | 0.09 |
| Type2 | SPI to EPI | Day 1 | change | -0.09 | -0.54 |
| Type2 | SPI to EPI | Day 2 | SPI | 0.40 | 0.10 |
| Type2 | SPI to EPI | Day 2 | EPI | 0.30 | 0.00 |
| Type2 | SPI to EPI | Day 2 | change | -0.10 | -0.40 |
| Type2 | SPI to EPI | Day 3 | SPI | 0.40 | 0.10 |
| Type2 | SPI to EPI | Day 3 | EPI | 0.30 | 0.10 |

| | | | | | |
|---|---|---|---|---|---|
| Type2 | SPI to EPI | Day 3 | change | 0.00 | -0.40 |
| Type2 | EPI to SPI | Overall | SPI | 0.35 | 0.27 |
| Type2 | EPI to SPI | Overall | EPI | 0.28 | 0.23 |
| Type2 | EPI to SPI | Overall | change | 0.07 | -0.01 |
| Type2 | EPI to SPI | Day 0 | SPI | 0.33 | 0.21 |
| Type2 | EPI to SPI | Day 0 | EPI | 0.24 | 0.17 |
| Type2 | EPI to SPI | Day 0 | change | 0.09 | -0.07 |
| Type2 | EPI to SPI | Day 1 | SPI | 0.34 | 0.23 |
| Type2 | EPI to SPI | Day 1 | EPI | 0.25 | 0.17 |
| Type2 | EPI to SPI | Day 1 | change | 0.08 | -0.07 |
| Type2 | EPI to SPI | Day 2 | SPI | 0.36 | 0.24 |
| Type2 | EPI to SPI | Day 2 | EPI | 0.29 | 0.20 |
| Type2 | EPI to SPI | Day 2 | change | 0.07 | -0.07 |
| Type2 | EPI to SPI | Day 3 | SPI | 0.38 | 0.23 |
| Type2 | EPI to SPI | Day 3 | EPI | 0.32 | 0.21 |
| Type2 | EPI to SPI | Day 3 | change | 0.06 | -0.10 |
| Type3 | NN to SPI | Overall | NN | 0.36 | 0.30 |
| Type3 | NN to SPI | Overall | SPI | 0.35 | 0.26 |
| Type3 | NN to SPI | Overall | change | -0.01 | -0.10 |
| Type3 | NN to SPI | Day 0 | NN | 0.60 | 0.49 |
| Type3 | NN to SPI | Day 0 | SPI | 0.48 | 0.34 |
| Type3 | NN to SPI | Day 0 | change | -0.12 | -0.27 |
| Type3 | NN to SPI | Day 1 | NN | 0.41 | 0.32 |
| Type3 | NN to SPI | Day 1 | SPI | 0.38 | 0.25 |
| Type3 | NN to SPI | Day 1 | change | -0.03 | -0.18 |
| Type3 | NN to SPI | Day 2 | NN | 0.24 | 0.16 |
| Type3 | NN to SPI | Day 2 | SPI | 0.29 | 0.18 |
| Type3 | NN to SPI | Day 2 | change | 0.05 | -0.11 |
| Type3 | NN to SPI | Day 3 | NN | 0.14 | 0.06 |
| Type3 | NN to SPI | Day 3 | SPI | 0.22 | 0.09 |
| Type3 | NN to SPI | Day 3 | change | 0.09 | -0.03 |
| Type3 | SPI to NN | Overall | NN | 0.31 | 0.12 |
| Type3 | SPI to NN | Overall | SPI | 0.27 | 0.12 |
| Type3 | SPI to NN | Overall | change | 0.04 | -0.19 |
| Type3 | SPI to NN | Day 0 | NN | 0.43 | 0.14 |
| Type3 | SPI to NN | Day 0 | SPI | 0.29 | 0.00 |
| Type3 | SPI to NN | Day 0 | change | 0.14 | -0.29 |

| | | | | | |
|---|---|---|---|---|---|
| Type3 | SPI to NN | Day 1 | NN | 0.38 | 0.12 |
| Type3 | SPI to NN | Day 1 | SPI | 0.25 | 0.00 |
| Type3 | SPI to NN | Day 1 | change | 0.00 | -0.38 |
| Type3 | SPI to NN | Day 2 | NN | 0.17 | 0.00 |
| Type3 | SPI to NN | Day 2 | SPI | 0.17 | 0.00 |
| Type3 | SPI to NN | Day 2 | change | 0.00 | -0.50 |
| Type3 | SPI to NN | Day 3 | NN | 0.20 | 0.00 |
| Type3 | SPI to NN | Day 3 | SPI | 0.20 | 0.00 |
| Type3 | SPI to NN | Day 3 | change | 0.00 | -0.60 |
| Type3 | NN to EPI | Overall | NN | 0.36 | 0.30 |
| Type3 | NN to EPI | Overall | EPI | 0.28 | 0.21 |
| Type3 | NN to EPI | Overall | change | -0.09 | -0.16 |
| Type3 | NN to EPI | Day 0 | NN | 0.60 | 0.51 |
| Type3 | NN to EPI | Day 0 | EPI | 0.38 | 0.26 |
| Type3 | NN to EPI | Day 0 | change | -0.22 | -0.38 |
| Type3 | NN to EPI | Day 1 | NN | 0.41 | 0.31 |
| Type3 | NN to EPI | Day 1 | EPI | 0.29 | 0.20 |
| Type3 | NN to EPI | Day 1 | change | -0.13 | -0.26 |
| Type3 | NN to EPI | Day 2 | NN | 0.24 | 0.17 |
| Type3 | NN to EPI | Day 2 | EPI | 0.23 | 0.11 |
| Type3 | NN to EPI | Day 2 | change | -0.02 | -0.16 |
| Type3 | NN to EPI | Day 3 | NN | 0.14 | 0.07 |
| Type3 | NN to EPI | Day 3 | EPI | 0.18 | 0.09 |
| Type3 | NN to EPI | Day 3 | change | 0.04 | -0.06 |
| Type3 | EPI to NN | Overall | NN | 0.32 | 0.10 |
| Type3 | EPI to NN | Overall | EPI | 0.21 | 0.00 |
| Type3 | EPI to NN | Overall | change | 0.05 | -0.21 |
| Type3 | EPI to NN | Day 0 | NN | 0.40 | 0.20 |
| Type3 | EPI to NN | Day 0 | EPI | 0.20 | 0.00 |
| Type3 | EPI to NN | Day 0 | change | 0.20 | -0.20 |
| Type3 | EPI to NN | Day 1 | NN | 0.40 | 0.00 |
| Type3 | EPI to NN | Day 1 | EPI | 0.20 | 0.00 |
| Type3 | EPI to NN | Day 1 | change | 0.20 | -0.20 |
| Type3 | EPI to NN | Day 2 | NN | 0.20 | 0.00 |
| Type3 | EPI to NN | Day 2 | EPI | 0.20 | 0.00 |
| Type3 | EPI to NN | Day 2 | change | 0.00 | -0.40 |
| Type3 | EPI to NN | Day 3 | NN | 0.25 | 0.00 |
| Type3 | EPI to NN | Day 3 | EPI | 0.25 | 0.00 |

| | | | | | |
|---|---|---|---|---|---|
| Type3 | EPI to NN | Day 3 | change | 0.00 | -0.75 |
| Type3 | SPI to EPI | Overall | SPI | 0.27 | 0.12 |
| Type3 | SPI to EPI | Overall | EPI | 0.23 | 0.08 |
| Type3 | SPI to EPI | Overall | change | -0.08 | -0.27 |
| Type3 | SPI to EPI | Day 0 | SPI | 0.29 | 0.00 |
| Type3 | SPI to EPI | Day 0 | EPI | 0.29 | 0.00 |
| Type3 | SPI to EPI | Day 0 | change | -0.14 | -0.43 |
| Type3 | SPI to EPI | Day 1 | SPI | 0.25 | 0.00 |
| Type3 | SPI to EPI | Day 1 | EPI | 0.25 | 0.00 |
| Type3 | SPI to EPI | Day 1 | change | -0.12 | -0.38 |
| Type3 | SPI to EPI | Day 2 | SPI | 0.17 | 0.00 |
| Type3 | SPI to EPI | Day 2 | EPI | 0.17 | 0.00 |
| Type3 | SPI to EPI | Day 2 | change | 0.00 | -0.50 |
| Type3 | SPI to EPI | Day 3 | SPI | 0.20 | 0.00 |
| Type3 | SPI to EPI | Day 3 | EPI | 0.20 | 0.00 |
| Type3 | SPI to EPI | Day 3 | change | 0.00 | -0.40 |
| Type3 | EPI to SPI | Overall | SPI | 0.32 | 0.10 |
| Type3 | EPI to SPI | Overall | EPI | 0.21 | 0.00 |
| Type3 | EPI to SPI | Overall | change | 0.05 | -0.21 |
| Type3 | EPI to SPI | Day 0 | SPI | 0.20 | 0.00 |
| Type3 | EPI to SPI | Day 0 | EPI | 0.20 | 0.00 |
| Type3 | EPI to SPI | Day 0 | change | 0.00 | -0.40 |
| Type3 | EPI to SPI | Day 1 | SPI | 0.20 | 0.00 |
| Type3 | EPI to SPI | Day 1 | EPI | 0.20 | 0.00 |
| Type3 | EPI to SPI | Day 1 | change | 0.00 | -0.40 |
| Type3 | EPI to SPI | Day 2 | SPI | 0.20 | 0.00 |
| Type3 | EPI to SPI | Day 2 | EPI | 0.20 | 0.00 |
| Type3 | EPI to SPI | Day 2 | change | 0.00 | -0.40 |
| Type3 | EPI to SPI | Day 3 | SPI | 0.25 | 0.00 |
| Type3 | EPI to SPI | Day 3 | EPI | 0.25 | 0.00 |
| Type3 | EPI to SPI | Day 3 | change | 0.00 | -0.50 |

# 5 Software and packages used

The model was implemented with the brms package in R which provides an interface to fit Bayesian generalized multivariate multilevel models using the probabilistic programming language Stan. cmdstanr package was used to interface with Stan from R. Packages used and version details are provided below.

```
## [1] "Fri Jul 22 14:56:15 2022"
```

```
## R version 4.2.1 (2022-06-23)
## Platform: x86_64-apple-darwin17.0 (64-bit)
## Running under: macOS Big Sur ... 10.16
##
## Matrix products: default
## BLAS:   /Library/Frameworks/R.framework/Versions/4.2/Resources/lib
/libRblas.0.dylib
## LAPACK: /Library/Frameworks/R.framework/Versions/4.2/Resources/lib
/libRlapack.dylib
##
## locale:
## [1] en_AU.UTF-8/en_AU.UTF-8/en_AU.UTF-8/C/en_AU.UTF-8/en_AU.UTF-8
##
## attached base packages:
## [1] stats     graphics  grDevices utils     datasets  methods   ba
se
##
## other attached packages:
##  [1] magrittr_2.0.3     lubridate_1.8.0    readxl_1.4.0       skim
r_2.1.4
##  [5] tictoc_1.0.1       bayesplot_1.9.0    bayestestR_0.12.1  quan
treg_5.93
##  [9] SparseM_1.81       brms_2.17.0        Rcpp_1.0.9         DT_0
.23
## [13] summarytools_1.0.1 plotrix_3.8-2      ggthemes_4.2.4     forc
ats_0.5.1
## [17] stringr_1.4.0      dplyr_1.0.9        purrr_0.3.4        read
r_2.1.2
## [21] tidyr_1.2.0        tibble_3.1.7       ggplot2_3.3.6      tidy
verse_1.3.2
##
## loaded via a namespace (and not attached):
##   [1] backports_1.4.1      plyr_1.8.7           igraph_1.3.3
##   [4] repr_1.1.4           splines_4.2.1        crosstalk_1.2.0
##   [7] rstantools_2.2.0     inline_0.3.19        pryr_0.1.5
##  [10] digest_0.6.29        htmltools_0.5.3      magick_2.7.3
##  [13] fansi_1.0.3          checkmate_2.1.0      googlesheets4_1.0.
0
##  [16] tzdb_0.3.0           modelr_0.1.8         RcppParallel_5.1.5
##  [19] matrixStats_0.62.0   xts_0.12.1           prettyunits_1.1.1
##  [22] colorspace_2.0-3     rvest_1.0.2          ggdist_3.1.1
##  [25] haven_2.5.0          xfun_0.31            tcltk_4.2.1
##  [28] callr_3.7.1          crayon_1.5.1         jsonlite_1.8.0
##  [31] survival_3.3-1       zoo_1.8-10           glue_1.6.2
##  [34] gtable_0.3.0         gargle_1.2.0         MatrixModels_0.5-0
##  [37] distributional_0.3.0 car_3.1-0            pkgbuild_1.3.1
##  [40] rstan_2.21.5         abind_1.4-5          rapportools_1.1
##  [43] scales_1.2.0         mvtnorm_1.1-3        DBI_1.1.3
##  [46] ggeffects_1.1.2      rstatix_0.7.0        miniUI_0.1.1.1
##  [49] xtable_1.8-4         HDInterval_0.2.2     stats4_4.2.1
##  [52] StanHeaders_2.21.0-7 datawizard_0.4.1     htmlwidgets_1.5.4
##  [55] httr_1.4.3           threejs_0.3.3        posterior_1.2.2
##  [58] ellipsis_0.3.2       pkgconfig_2.0.3      loo_2.5.1
##  [61] farver_2.1.1         sass_0.4.2           dbplyr_2.2.1
##  [64] utf8_1.2.2           labeling_0.4.2       tidyselect_1.1.2
##  [67] rlang_1.0.4          reshape2_1.4.4       later_1.3.0
##  [70] munsell_0.5.0        cellranger_1.1.0     tools_4.2.1
##  [73] cachem_1.0.6         cli_3.3.0            generics_0.1.3
##  [76] broom_1.0.0          ggridges_0.5.3       evaluate_0.15
##  [79] fastmap_1.1.0        yaml_2.3.5           processx_3.7.0
##  [82] knitr_1.39           fs_1.5.2             pander_0.6.5
##  [85] nlme_3.1-158         mime_0.12            xml2_1.3.3
```

```
##  [88] compiler_4.2.1       shinythemes_1.2.0    rstudioapi_0.13
##  [91] ggsignif_0.6.3       reprex_2.0.1         bslib_0.4.0
##  [94] stringi_1.7.8        highr_0.9            ps_1.7.1
##  [97] Brobdingnag_1.2-7    lattice_0.20-45      Matrix_1.4-1
## [100] markdown_1.1         shinyjs_2.1.0        tensorA_0.36.2
## [103] vctrs_0.4.1          pillar_1.8.0         lifecycle_1.0.1
## [106] jquerylib_0.1.4      bridgesampling_1.1-2 cowplot_1.1.1
## [109] data.table_1.14.2    insight_0.18.0       httpuv_1.6.5
## [112] R6_2.5.1             promises_1.2.0.1     gridExtra_2.3
## [115] codetools_0.2-18     colourpicker_1.1.1   MASS_7.3-58
## [118] gtools_3.9.3         assertthat_0.2.1     withr_2.5.0
## [121] shinystan_2.6.0      parallel_4.2.1       hms_1.1.1
## [124] grid_4.2.1           coda_0.19-4          cmdstanr_0.5.2
## [127] rmarkdown_2.14       carData_3.0-5        googledrive_2.0.0
## [130] ggpubr_0.4.0         shiny_1.7.2          base64enc_0.1-3
## [133] dygraphs_1.1.1.6
```

```
## 1608.433 sec elapsed
```
