# Supplementary material for: Postoperative analgesia for upper gastrointestinal surgery: a retrospective cohort analysis
Source: Perioper Med (Lond). 2023 Jul 18;12:40. doi: 10.1186/s13741-023-00324-0 (PMC10355044; doi:10.1186/s13741-023-00324-0)
Supplement: Supplementary file 1 — Additional file 1. a Post-operative morphine requirements. b. Post-operative rest pain. c Post-operative dynamic pain. [file 13741_2023_324_MOESM1_ESM.zip › Additional file 1c.pdf]

1 The Data
2 Goals of the analysis
3 The Model
4 Posterior Distributions of Dynamic Pain Scores
5 Software and packages used

Code

Supplementary Material 1c

Post-operative Dynamic Pain

1 The Data

The data are ***dynamic pain scores*** on day 0, 1, 2 and 3 after surgery. They are on an ordinal scale from 0 to 10. To avoid mistaken them as on a numerical scale, I've labelled them in words from "zero" to "ten", so arithmic calculations aren't possible.
The data displayed below in the frequency table is the count of a particular pain score on a particular day.

PSd: Pain Score (dynamic)
NN: Non-neuraxial
SPI: Intrathecal Morphine
EPI: Thoracic Epidural Analgesia

Table 1

```
##        Days
## PSd      0  1  2  3
##   Zero  65 30 21 25
##   One   21 10 11 10
##   Two   13 19 23 20
##   Three 22 25 36 40
##   Four  21 42 42 47
##   Five  30 55 44 50
##   Six   24 50 61 38
##   Seven 27 53 37 26
##   Eight 26 36 31 20
##   Nine   9 21 16 12
##   Ten   15 25 11  6
```

The data is again displayed graphically below.

The first plot shows a bar graph of the proportion of counts for dynamic pain scores over different post operative days across different analgesic techniques. The red bars represent the pain score with the most counts.

Figure 1

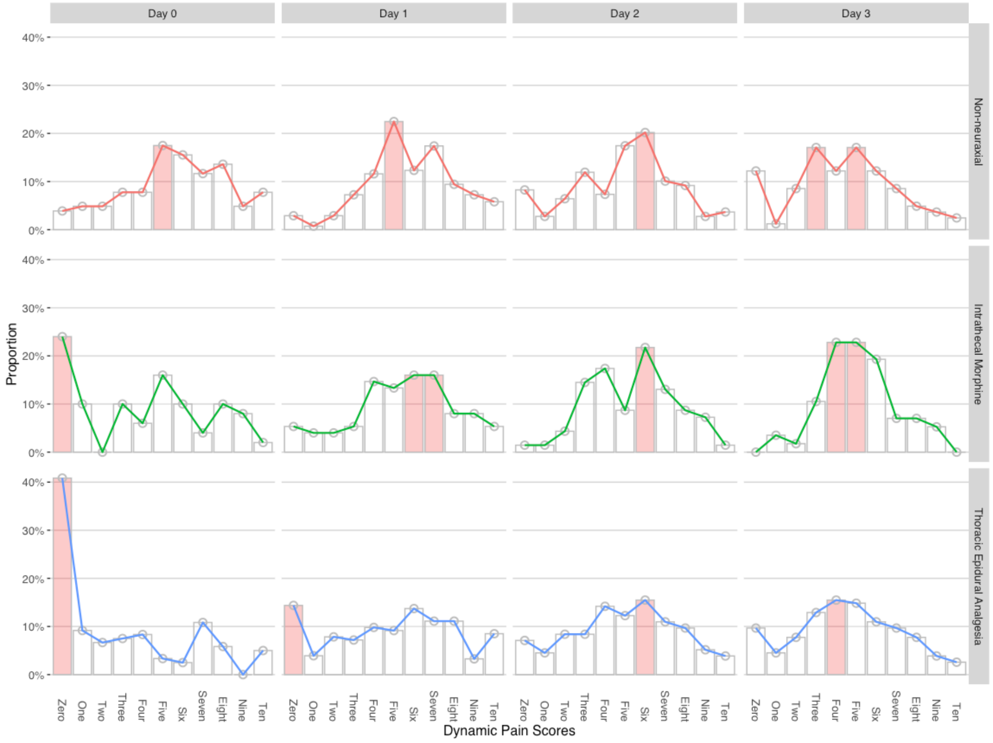

The plot below shows the same information, this time with cumulative proportion. The vertical line shows the proportion of patients scoring the respective dynamic PS. The PS with the largest proportion is represented by the red line.

Figure 2

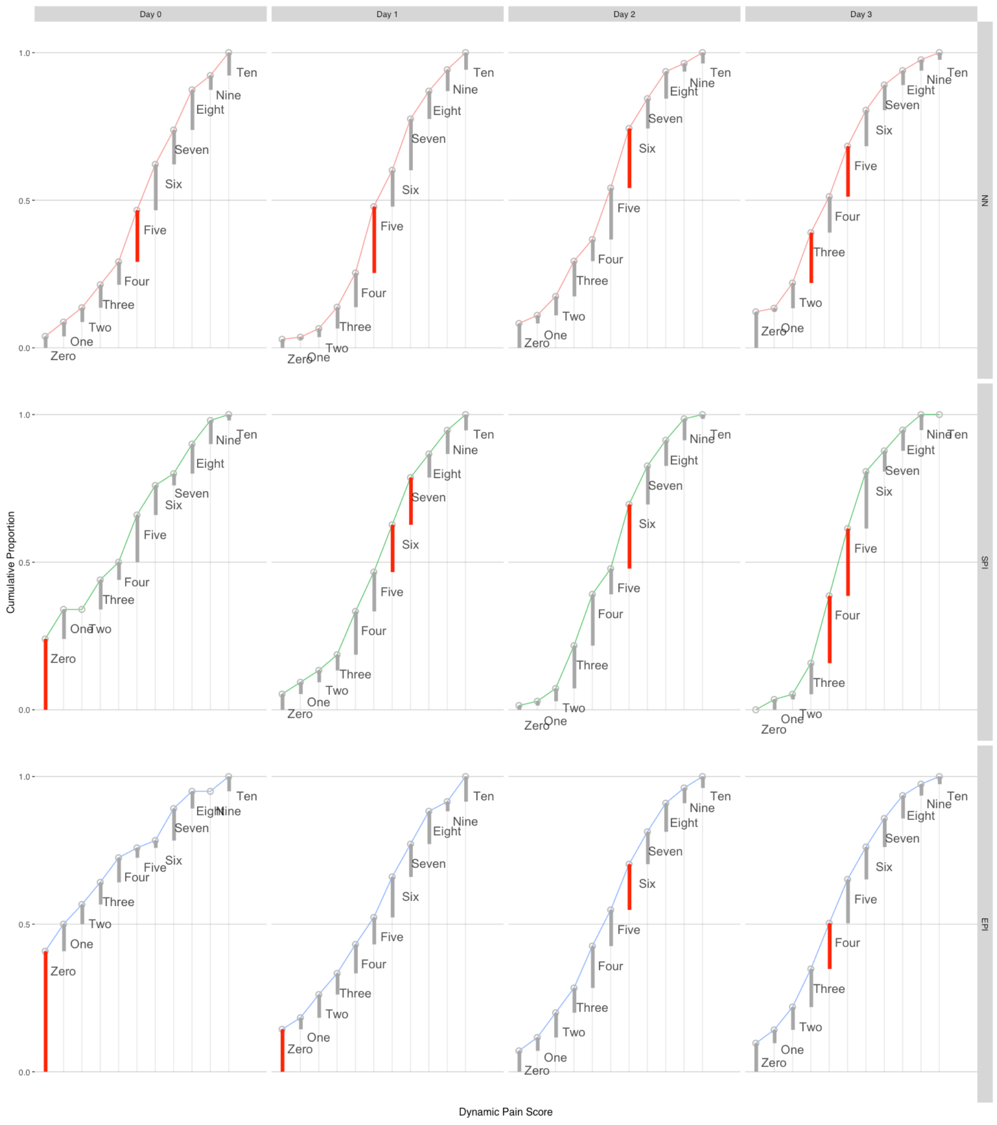

2 Goals of the analysis

We are interested in

1. Dynamic pain score distributions with different analgesic techniques over the post-operative period
2. Probability of poor pain control
3. Probability of poor pain control if our threshold is different

We consider pain score of 4 or less as adequate pain control.

3 The Model

The outcome variable dynamic pain scores (PSd) are ordinal such that pain score (PS) of $zero < one < two < three < \ldots < ten$. The interval between the pain scores are not necessarily of the same distance. That is, the increase in pain from a PS of 1 to 2 may not be of the same magnitude to that from a PS of 5 to 6. Hence, we will model PSd with an ordinal cumulative model.

The cumulative model assumes that the observed ordinal variable Y originates from the categorization of a latent continuous variable $\tilde{Y}$, such that there are latent thresholds θ_k that partition the values into the $k+1$ observable ordered categories of Y (i.e. ordered pain score categories of zero, one, two, three… and so on to a pain score category of ten). If we use η to symbolise the predictor terms, such that $\eta = \beta X$, and F being a cumulative distribution function, then

$$Pr(Y = k|\eta) = F(\theta_k - \eta) - F(\theta_{k-1} - \eta)$$

Note this cumulative model assumes that the predictor term η is constant across the response categories. For our model, we will use the $logit$ link function for its ease of interpretation of the coefficients as odds ratios.

3.1 Model specifications

The data generating process for scoring a particular PS should be the same as for the OMED requirements. We will therefore use the same mixed effect structure for η with the same predictor variables and interaction terms.

For the priors, we will set a common prior for each intercept which assigns probability to scoring the 11 categories of pain score more evenly. This choice is examined by comparing different prior values as shown below. To find a prior that roughly assigns equal probability to each of the pain score, we will compare different prior values from $\sim N(0, 0.5)$ to $\sim N(0, 11)$ in sequential sequence by 0.5. One can see that as the prior σ increases from 0.5 to 11, the proportion of assignment of the pain score changes from assigning more on the extreme values to more in the middle. The bars are the data pain score distribution. The blue points/lines are the median of prior posterior draw predictions (without the influence of the data), and the error bars are 99% credible intervals for the prior prediction draws. A prior of $\sim N(0.5)$ seems to be reasonably spread out with coverage up to 50% proportion for each pain score.

Figure 3

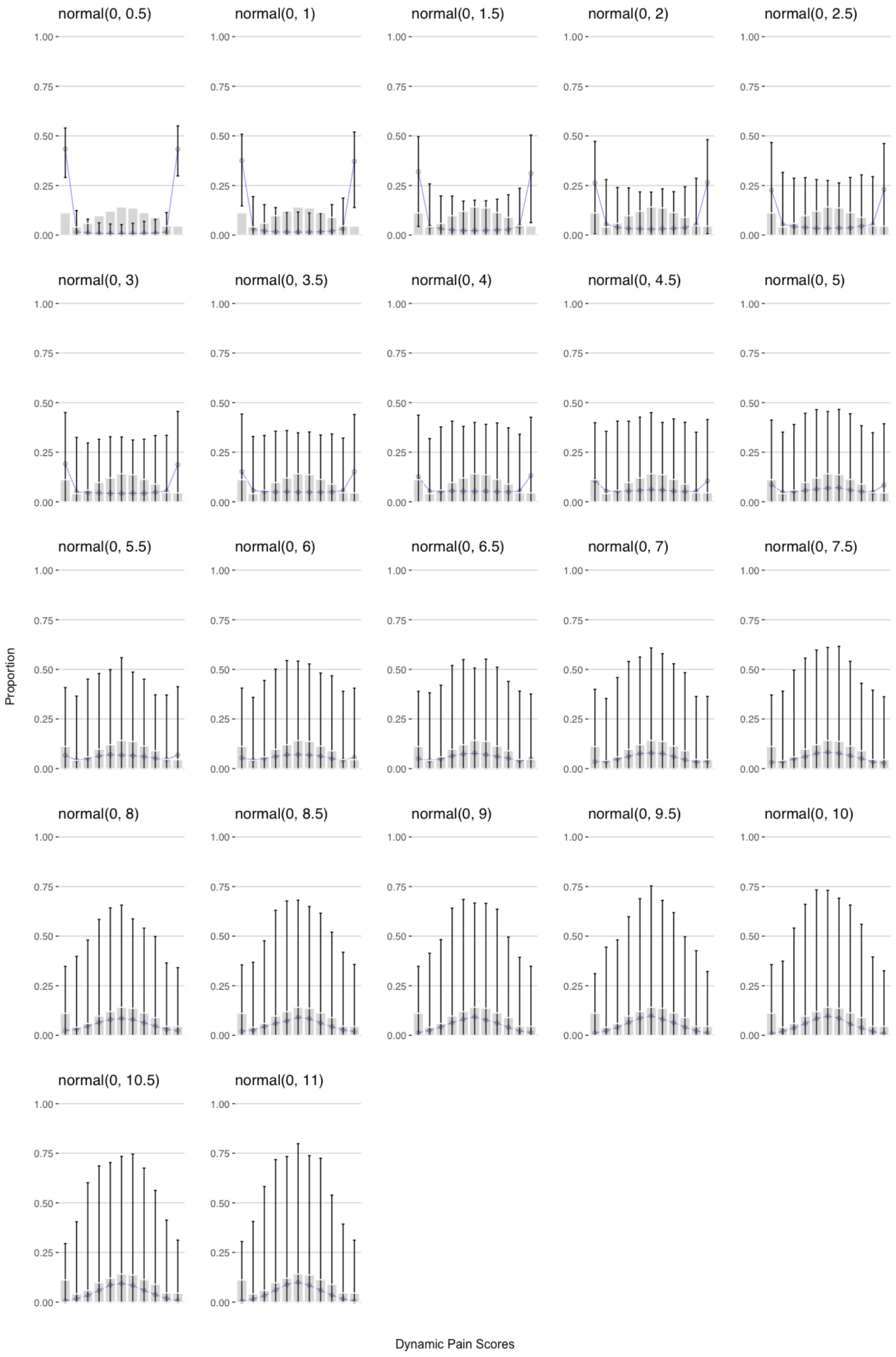

As for the β effect, we will assign the prior to $\sim Normal(0, 0.5)$, which assigns prior probability to be higher towards zero difference, but still encompass reasonable probability difference effects. These are weakly informative priors that consist of reasonable, non-extreme probability assignments.

$$
\begin{aligned}
PS_i &\sim Categorical(\mathbf{p}) \\
p_1 &= q_1 \\
p_k &= q_k - q_{k-1} && for\ K > k > 1 \\
logit(q_k) &= \theta_k - \eta_i \\
\eta_i &= \beta_{ID[i]} \\
\theta_k &\sim Normal(0, 5) \\
\beta_j &\sim Normal(0, 0.5)
\end{aligned}
$$

3.2 Prior predictive check

This is the same as the comparison plot above, zoomed in to the prior predictive distribution when we set the intercept prior to be $\sim Normal(0, 5)$.

It can be seen that the prior roughly evenly distributed the probability to each of the 11 pain scores and covers a reasonable probability of scoring a particular pain score. There is also no unreasonable values such as negative proportions.

Figure 4

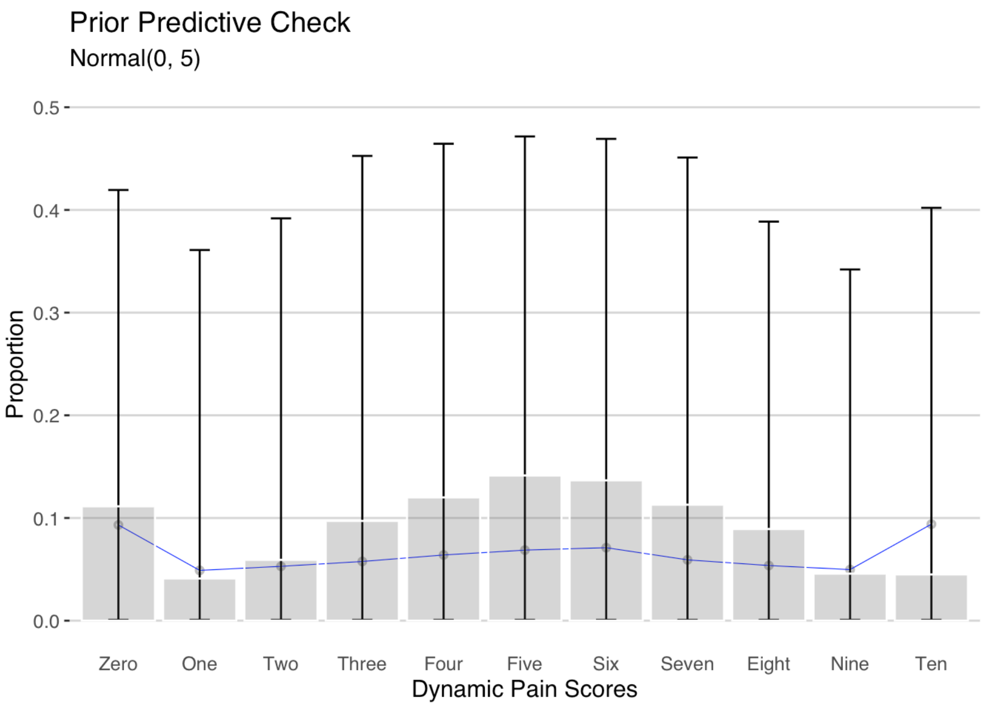

3.3 Posterior predictive check

It can be seen from the plot that the posteriors describes the data very well.

Figure 5

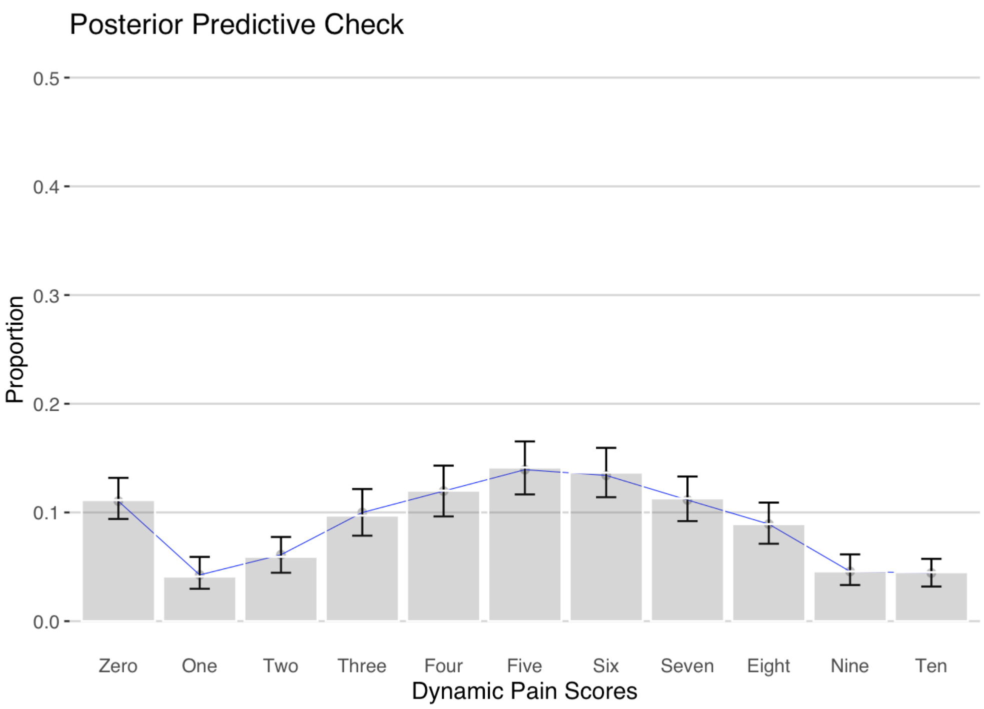

4 Posterior Distributions of Dynamic Pain Scores

4.1 Adjusted dynamic pain scores

4.1.1 Overal distribution of dynamic pain scores over the 4 post-operative time points and with different analgesic techniques

The plot shows the distribution of post-op dynamic pain scores for a typical patient who is

```
- 60 years of age
- Female
- ASA = 3
- Surgical Approach = Open
- Surgical type = Type 1
```

Figure 6

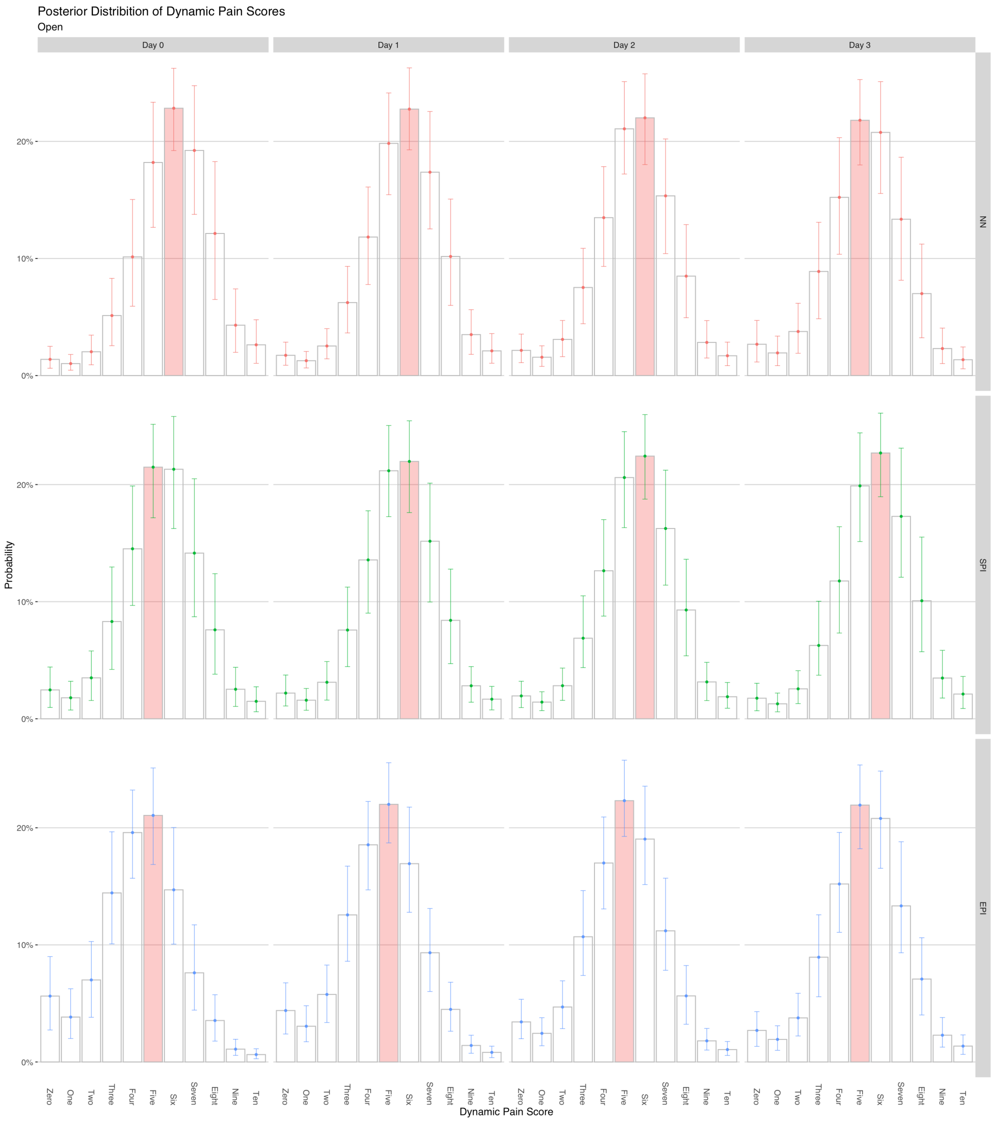

Table 2

Days	Group	PS	Probability	95%CrI.upper	95%CrI.lower	50%CrI.upper	50%CrI.low
0	NN	Zero	0.01	0.02	0.01	0.02	0
0	NN	One	0.01	0.02	0.00	0.01	0
0	NN	Two	0.02	0.03	0.01	0.02	0
0	NN	Three	0.05	0.08	0.03	0.06	0
0	NN	Four	0.10	0.15	0.06	0.11	0
0	NN	Five	0.18	0.23	0.13	0.20	0
0	NN	Six	0.23	0.26	0.19	0.24	0
0	NN	Seven	0.19	0.25	0.14	0.21	0
0	NN	Eight	0.12	0.18	0.07	0.14	0
0	NN	Nine	0.04	0.07	0.02	0.05	0
0	NN	Ten	0.03	0.05	0.01	0.03	0
0	SPI	Zero	0.02	0.04	0.01	0.03	0

0	SPI	One	0.02	0.03	0.01	0.02	0
0	SPI	Two	0.03	0.06	0.02	0.04	0
0	SPI	Three	0.08	0.13	0.04	0.09	0
0	SPI	Four	0.15	0.20	0.10	0.17	0
0	SPI	Five	0.21	0.25	0.17	0.23	0
0	SPI	Six	0.21	0.26	0.16	0.23	0
0	SPI	Seven	0.14	0.20	0.09	0.16	0
0	SPI	Eight	0.08	0.12	0.04	0.09	0
0	SPI	Nine	0.03	0.04	0.01	0.03	0
0	SPI	Ten	0.01	0.03	0.01	0.02	0
0	EPI	Zero	0.06	0.09	0.03	0.07	0
0	EPI	One	0.04	0.06	0.02	0.04	0
0	EPI	Two	0.07	0.10	0.04	0.08	0
0	EPI	Three	0.14	0.20	0.10	0.16	0
0	EPI	Four	0.20	0.23	0.16	0.21	0
0	EPI	Five	0.21	0.25	0.17	0.22	0
0	EPI	Six	0.15	0.20	0.10	0.16	0
0	EPI	Seven	0.08	0.12	0.04	0.09	0
0	EPI	Eight	0.04	0.06	0.02	0.04	0
0	EPI	Nine	0.01	0.02	0.01	0.01	0
0	EPI	Ten	0.01	0.01	0.00	0.01	0
1	NN	Zero	0.02	0.03	0.01	0.02	0
1	NN	One	0.01	0.02	0.01	0.01	0
1	NN	Two	0.03	0.04	0.01	0.03	0
1	NN	Three	0.06	0.09	0.04	0.07	0
1	NN	Four	0.12	0.16	0.08	0.13	0
1	NN	Five	0.20	0.24	0.15	0.21	0
1	NN	Six	0.23	0.26	0.19	0.24	0
1	NN	Seven	0.17	0.23	0.13	0.19	0
1	NN	Eight	0.10	0.15	0.06	0.12	0
1	NN	Nine	0.03	0.06	0.02	0.04	0
1	NN	Ten	0.02	0.04	0.01	0.02	0
1	SPI	Zero	0.02	0.04	0.01	0.02	0
1	SPI	One	0.02	0.03	0.01	0.02	0
1	SPI	Two	0.03	0.05	0.02	0.04	0
1	SPI	Three	0.08	0.11	0.04	0.09	0
1	SPI	Four	0.14	0.18	0.09	0.15	0
1	SPI	Five	0.21	0.25	0.17	0.23	0

1	SPI	Six	0.22	0.25	0.18	0.24	0
1	SPI	Seven	0.15	0.20	0.10	0.17	0
1	SPI	Eight	0.08	0.13	0.05	0.10	0
1	SPI	Nine	0.03	0.04	0.01	0.03	0
1	SPI	Ten	0.02	0.03	0.01	0.02	0
1	EPI	Zero	0.04	0.07	0.02	0.05	0
1	EPI	One	0.03	0.05	0.02	0.03	0
1	EPI	Two	0.06	0.08	0.03	0.06	0
1	EPI	Three	0.13	0.17	0.09	0.14	0
1	EPI	Four	0.19	0.22	0.15	0.20	0
1	EPI	Five	0.22	0.26	0.19	0.23	0
1	EPI	Six	0.17	0.22	0.13	0.18	0
1	EPI	Seven	0.09	0.13	0.06	0.10	0
1	EPI	Eight	0.04	0.07	0.03	0.05	0
1	EPI	Nine	0.01	0.02	0.01	0.02	0
1	EPI	Ten	0.01	0.01	0.00	0.01	0
2	NN	Zero	0.02	0.04	0.01	0.02	0
2	NN	One	0.02	0.03	0.01	0.02	0
2	NN	Two	0.03	0.05	0.02	0.03	0
2	NN	Three	0.08	0.11	0.04	0.08	0
2	NN	Four	0.13	0.18	0.09	0.15	0
2	NN	Five	0.21	0.25	0.17	0.22	0
2	NN	Six	0.22	0.26	0.18	0.24	0
2	NN	Seven	0.15	0.20	0.10	0.17	0
2	NN	Eight	0.08	0.13	0.05	0.09	0
2	NN	Nine	0.03	0.05	0.01	0.03	0
2	NN	Ten	0.02	0.03	0.01	0.02	0
2	SPI	Zero	0.02	0.03	0.01	0.02	0
2	SPI	One	0.01	0.02	0.01	0.02	0
2	SPI	Two	0.03	0.04	0.02	0.03	0
2	SPI	Three	0.07	0.10	0.04	0.08	0
2	SPI	Four	0.13	0.17	0.09	0.14	0
2	SPI	Five	0.21	0.25	0.16	0.22	0
2	SPI	Six	0.22	0.26	0.19	0.24	0
2	SPI	Seven	0.16	0.21	0.11	0.18	0
2	SPI	Eight	0.09	0.14	0.05	0.10	0
2	SPI	Nine	0.03	0.05	0.02	0.04	0
2	SPI	Ten	0.02	0.03	0.01	0.02	0

2	EPI	Zero	0.03	0.05	0.02	0.04	0
2	EPI	One	0.02	0.04	0.01	0.03	0
2	EPI	Two	0.05	0.07	0.03	0.05	0
2	EPI	Three	0.11	0.15	0.07	0.12	0
2	EPI	Four	0.17	0.21	0.13	0.19	0
2	EPI	Five	0.22	0.26	0.19	0.23	0
2	EPI	Six	0.19	0.24	0.15	0.21	0
2	EPI	Seven	0.11	0.16	0.08	0.12	0
2	EPI	Eight	0.06	0.08	0.03	0.06	0
2	EPI	Nine	0.02	0.03	0.01	0.02	0
2	EPI	Ten	0.01	0.02	0.01	0.01	0
3	NN	Zero	0.03	0.05	0.01	0.03	0
3	NN	One	0.02	0.03	0.01	0.02	0
3	NN	Two	0.04	0.06	0.02	0.04	0
3	NN	Three	0.09	0.13	0.05	0.10	0
3	NN	Four	0.15	0.20	0.10	0.16	0
3	NN	Five	0.22	0.25	0.18	0.23	0
3	NN	Six	0.21	0.25	0.16	0.23	0
3	NN	Seven	0.13	0.19	0.08	0.15	0
3	NN	Eight	0.07	0.11	0.03	0.08	0
3	NN	Nine	0.02	0.04	0.01	0.03	0
3	NN	Ten	0.01	0.02	0.01	0.02	0
3	SPI	Zero	0.02	0.03	0.01	0.02	0
3	SPI	One	0.01	0.02	0.01	0.01	0
3	SPI	Two	0.03	0.04	0.01	0.03	0
3	SPI	Three	0.06	0.10	0.04	0.07	0
3	SPI	Four	0.12	0.16	0.07	0.13	0
3	SPI	Five	0.20	0.24	0.15	0.22	0
3	SPI	Six	0.23	0.26	0.19	0.24	0
3	SPI	Seven	0.17	0.23	0.12	0.19	0
3	SPI	Eight	0.10	0.16	0.06	0.11	0
3	SPI	Nine	0.03	0.06	0.02	0.04	0
3	SPI	Ten	0.02	0.04	0.01	0.02	0
3	EPI	Zero	0.03	0.04	0.01	0.03	0
3	EPI	One	0.02	0.03	0.01	0.02	0
3	EPI	Two	0.04	0.06	0.02	0.04	0
3	EPI	Three	0.09	0.13	0.06	0.10	0
3	EPI	Four	0.15	0.20	0.11	0.17	0

3	EPI	Five	0.22	0.25	0.18	0.23	0
3	EPI	Six	0.21	0.25	0.17	0.22	0
3	EPI	Seven	0.13	0.19	0.09	0.15	0
3	EPI	Eight	0.07	0.11	0.04	0.08	0
3	EPI	Nine	0.02	0.04	0.01	0.03	0
3	EPI	Ten	0.01	0.02	0.01	0.02	0

In comparison, we will plot the probability distribution of dynamic pain scores across the 4 day post-op period a typical patient who has **laparoscopic surgery** instead:

```
- 60 years of age
- Female
- ASA = 3
- Surgical Approach = Laparoscopic
- Surgical type = Type 1
```

Figure 7

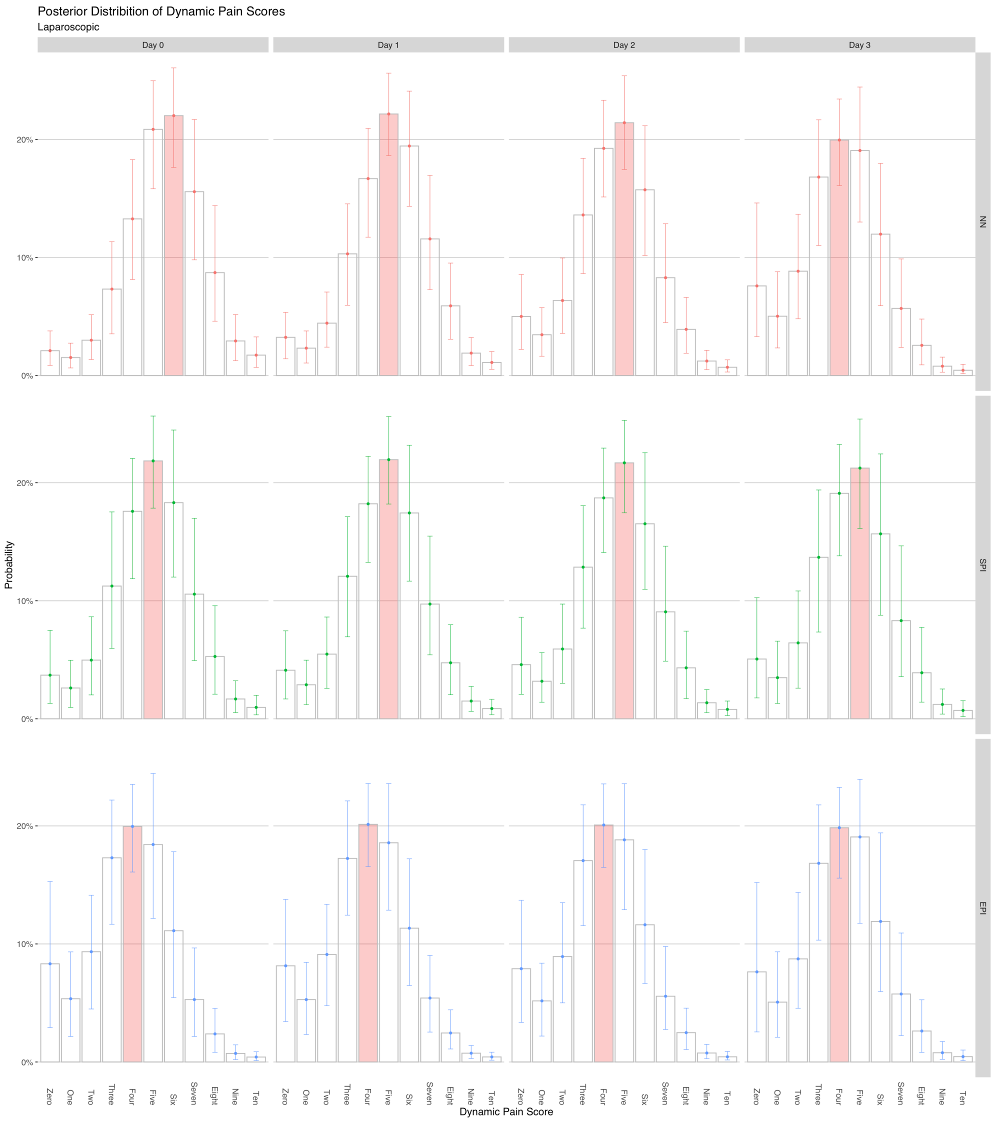

Table 3

Days	Group	PS	Probability	95%CrI.upper	95%CrI.lower	50%CrI.upper	50%CrI.low
0	NN	Zero	0.02	0.04	0.01	0.02	0
0	NN	One	0.02	0.03	0.01	0.02	0
0	NN	Two	0.03	0.05	0.01	0.03	0
0	NN	Three	0.07	0.11	0.04	0.08	0
0	NN	Four	0.13	0.18	0.08	0.15	0
0	NN	Five	0.21	0.25	0.16	0.22	0
0	NN	Six	0.22	0.26	0.18	0.23	0
0	NN	Seven	0.16	0.22	0.10	0.17	0
0	NN	Eight	0.09	0.14	0.05	0.10	0
0	NN	Nine	0.03	0.05	0.01	0.03	0
0	NN	Ten	0.02	0.03	0.01	0.02	0
0	SPI	Zero	0.04	0.07	0.01	0.04	0

0	SPI	One	0.03	0.05	0.01	0.03	0
0	SPI	Two	0.05	0.09	0.02	0.06	0
0	SPI	Three	0.11	0.18	0.06	0.13	0
0	SPI	Four	0.18	0.22	0.12	0.20	0
0	SPI	Five	0.22	0.26	0.18	0.23	0
0	SPI	Six	0.18	0.24	0.12	0.21	0
0	SPI	Seven	0.11	0.17	0.05	0.12	0
0	SPI	Eight	0.05	0.10	0.02	0.06	0
0	SPI	Nine	0.02	0.03	0.01	0.02	0
0	SPI	Ten	0.01	0.02	0.00	0.01	0
0	EPI	Zero	0.08	0.15	0.03	0.09	0
0	EPI	One	0.05	0.09	0.02	0.06	0
0	EPI	Two	0.09	0.14	0.04	0.11	0
0	EPI	Three	0.17	0.22	0.12	0.20	0
0	EPI	Four	0.20	0.24	0.16	0.21	0
0	EPI	Five	0.18	0.24	0.12	0.21	0
0	EPI	Six	0.11	0.18	0.05	0.13	0
0	EPI	Seven	0.05	0.10	0.02	0.06	0
0	EPI	Eight	0.02	0.05	0.01	0.03	0
0	EPI	Nine	0.01	0.01	0.00	0.01	0
0	EPI	Ten	0.00	0.01	0.00	0.00	0
1	NN	Zero	0.03	0.05	0.01	0.04	0
1	NN	One	0.02	0.04	0.01	0.03	0
1	NN	Two	0.04	0.07	0.02	0.05	0
1	NN	Three	0.10	0.15	0.06	0.12	0
1	NN	Four	0.17	0.21	0.12	0.18	0
1	NN	Five	0.22	0.26	0.19	0.23	0
1	NN	Six	0.19	0.24	0.14	0.21	0
1	NN	Seven	0.12	0.17	0.07	0.13	0
1	NN	Eight	0.06	0.10	0.03	0.07	0
1	NN	Nine	0.02	0.03	0.01	0.02	0
1	NN	Ten	0.01	0.02	0.01	0.01	0
1	SPI	Zero	0.04	0.07	0.02	0.05	0
1	SPI	One	0.03	0.05	0.01	0.03	0
1	SPI	Two	0.05	0.09	0.03	0.06	0
1	SPI	Three	0.12	0.17	0.07	0.14	0
1	SPI	Four	0.18	0.22	0.13	0.20	0
1	SPI	Five	0.22	0.26	0.18	0.23	0

1	SPI	Six	0.17	0.23	0.12	0.19	0
1	SPI	Seven	0.10	0.15	0.05	0.11	0
1	SPI	Eight	0.05	0.08	0.02	0.05	0
1	SPI	Nine	0.02	0.03	0.01	0.02	0
1	SPI	Ten	0.01	0.02	0.00	0.01	0
1	EPI	Zero	0.08	0.14	0.03	0.09	0
1	EPI	One	0.05	0.08	0.02	0.06	0
1	EPI	Two	0.09	0.13	0.05	0.11	0
1	EPI	Three	0.17	0.22	0.12	0.19	0
1	EPI	Four	0.20	0.24	0.17	0.21	0
1	EPI	Five	0.19	0.24	0.13	0.20	0
1	EPI	Six	0.11	0.17	0.06	0.12	0
1	EPI	Seven	0.05	0.09	0.03	0.06	0
1	EPI	Eight	0.02	0.04	0.01	0.03	0
1	EPI	Nine	0.01	0.01	0.00	0.01	0
1	EPI	Ten	0.00	0.01	0.00	0.00	0
2	NN	Zero	0.05	0.09	0.02	0.06	0
2	NN	One	0.03	0.06	0.02	0.04	0
2	NN	Two	0.06	0.10	0.04	0.07	0
2	NN	Three	0.14	0.18	0.09	0.15	0
2	NN	Four	0.19	0.23	0.15	0.21	0
2	NN	Five	0.21	0.25	0.17	0.23	0
2	NN	Six	0.16	0.21	0.10	0.17	0
2	NN	Seven	0.08	0.13	0.04	0.10	0
2	NN	Eight	0.04	0.07	0.02	0.04	0
2	NN	Nine	0.01	0.02	0.01	0.01	0
2	NN	Ten	0.01	0.01	0.00	0.01	0
2	SPI	Zero	0.05	0.09	0.02	0.05	0
2	SPI	One	0.03	0.06	0.01	0.04	0
2	SPI	Two	0.06	0.10	0.03	0.07	0
2	SPI	Three	0.13	0.18	0.08	0.14	0
2	SPI	Four	0.19	0.23	0.14	0.20	0
2	SPI	Five	0.22	0.25	0.17	0.23	0
2	SPI	Six	0.17	0.23	0.11	0.18	0
2	SPI	Seven	0.09	0.15	0.05	0.11	0
2	SPI	Eight	0.04	0.07	0.02	0.05	0
2	SPI	Nine	0.01	0.02	0.01	0.02	0
2	SPI	Ten	0.01	0.02	0.00	0.01	0

2	EPI	Zero	0.08	0.14	0.03	0.09	0
2	EPI	One	0.05	0.08	0.02	0.06	0
2	EPI	Two	0.09	0.13	0.05	0.10	0
2	EPI	Three	0.17	0.22	0.12	0.19	0
2	EPI	Four	0.20	0.24	0.16	0.21	0
2	EPI	Five	0.19	0.24	0.13	0.21	0
2	EPI	Six	0.12	0.18	0.07	0.13	0
2	EPI	Seven	0.06	0.10	0.03	0.06	0
2	EPI	Eight	0.02	0.05	0.01	0.03	0
2	EPI	Nine	0.01	0.01	0.00	0.01	0
2	EPI	Ten	0.00	0.01	0.00	0.01	0
3	NN	Zero	0.08	0.15	0.03	0.09	0
3	NN	One	0.05	0.09	0.02	0.06	0
3	NN	Two	0.09	0.14	0.05	0.10	0
3	NN	Three	0.17	0.22	0.11	0.19	0
3	NN	Four	0.20	0.23	0.16	0.21	0
3	NN	Five	0.19	0.24	0.13	0.21	0
3	NN	Six	0.12	0.18	0.06	0.13	0
3	NN	Seven	0.06	0.10	0.02	0.06	0
3	NN	Eight	0.03	0.05	0.01	0.03	0
3	NN	Nine	0.01	0.02	0.00	0.01	0
3	NN	Ten	0.00	0.01	0.00	0.01	0
3	SPI	Zero	0.05	0.10	0.02	0.06	0
3	SPI	One	0.03	0.07	0.01	0.04	0
3	SPI	Two	0.06	0.11	0.03	0.08	0
3	SPI	Three	0.14	0.19	0.07	0.16	0
3	SPI	Four	0.19	0.23	0.14	0.21	0
3	SPI	Five	0.21	0.25	0.16	0.23	0
3	SPI	Six	0.16	0.22	0.09	0.18	0
3	SPI	Seven	0.08	0.15	0.04	0.09	0
3	SPI	Eight	0.04	0.08	0.01	0.05	0
3	SPI	Nine	0.01	0.03	0.00	0.01	0
3	SPI	Ten	0.01	0.02	0.00	0.01	0
3	EPI	Zero	0.08	0.15	0.03	0.09	0
3	EPI	One	0.05	0.09	0.02	0.06	0
3	EPI	Two	0.09	0.14	0.05	0.10	0
3	EPI	Three	0.17	0.22	0.10	0.20	0
3	EPI	Four	0.20	0.23	0.16	0.21	0

3	EPI	Five	0.19	0.24	0.12	0.22	0
3	EPI	Six	0.12	0.19	0.06	0.14	0
3	EPI	Seven	0.06	0.11	0.02	0.06	0
3	EPI	Eight	0.03	0.05	0.01	0.03	0
3	EPI	Nine	0.01	0.02	0.00	0.01	0
3	EPI	Ten	0.00	0.01	0.00	0.01	0

4.2 Probability of adequate pain relief

We define adequate pain relief as a pain score of 4 or less.
Hence, PS of 5 or more are considered poor dynamic pain control.

4.2.1 Probability of poor dynamic pain control

We determine the probability of poor dynamic pain control if one were to have a different analgesic technique.
The plot shows the posterior predictive distribution of the probability of poor pain control of the original analgesic technique, the posterior predictive distribution of the probability of poor pain control if one were to have a different analgesic technique, and the distribution of the differences in the probability of poor pain control (grey). The black error bars reflect the 95% credible intervals.

Figure 8

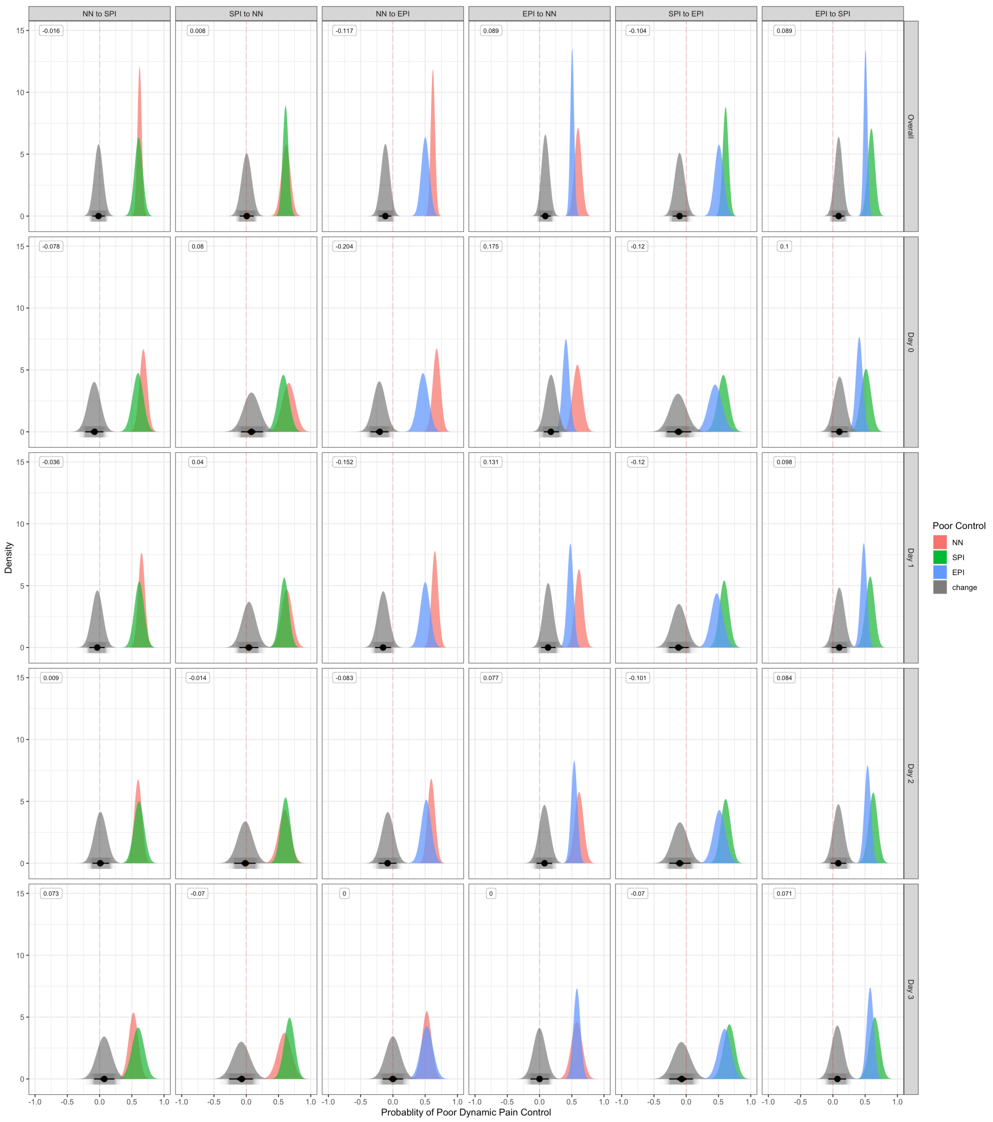

Table 4

AnalgesicTechniques	Day	Analg	ProbPoorControl.median	CI95low	CI95high
NN to SPI	Overall	NN	0.62	0.58	0.66
NN to SPI	Overall	SPI	0.61	0.51	0.68
NN to SPI	Overall	change	-0.02	-0.11	0.08
NN to SPI	Day 0	NN	0.68	0.59	0.76
NN to SPI	Day 0	SPI	0.59	0.48	0.71
NN to SPI	Day 0	change	-0.08	-0.22	0.05
NN to SPI	Day 1	NN	0.65	0.58	0.72
NN to SPI	Day 1	SPI	0.62	0.51	0.71
NN to SPI	Day 1	change	-0.04	-0.15	0.08
NN to SPI	Day 2	NN	0.60	0.50	0.67
NN to SPI	Day 2	SPI	0.62	0.49	0.71
NN to SPI	Day 2	change	0.01	-0.11	0.15

NN to SPI	Day 3	NN	0.52	0.42	0.62
NN to SPI	Day 3	SPI	0.60	0.46	0.72
NN to SPI	Day 3	change	0.07	-0.10	0.22
SPI to NN	Overall	NN	0.62	0.51	0.70
SPI to NN	Overall	SPI	0.61	0.55	0.67
SPI to NN	Overall	change	0.01	-0.10	0.11
SPI to NN	Day 0	NN	0.66	0.52	0.78
SPI to NN	Day 0	SPI	0.58	0.46	0.70
SPI to NN	Day 0	change	0.08	-0.08	0.26
SPI to NN	Day 1	NN	0.63	0.49	0.73
SPI to NN	Day 1	SPI	0.59	0.49	0.68
SPI to NN	Day 1	change	0.04	-0.12	0.17
SPI to NN	Day 2	NN	0.59	0.46	0.71
SPI to NN	Day 2	SPI	0.61	0.49	0.70
SPI to NN	Day 2	change	-0.01	-0.19	0.13
SPI to NN	Day 3	NN	0.60	0.46	0.74
SPI to NN	Day 3	SPI	0.67	0.54	0.77
SPI to NN	Day 3	change	-0.07	-0.26	0.10
NN to EPI	Overall	NN	0.62	0.57	0.66
NN to EPI	Overall	EPI	0.50	0.42	0.59
NN to EPI	Overall	change	-0.12	-0.21	-0.02
NN to EPI	Day 0	NN	0.68	0.58	0.75
NN to EPI	Day 0	EPI	0.47	0.35	0.58
NN to EPI	Day 0	change	-0.20	-0.35	-0.08
NN to EPI	Day 1	NN	0.65	0.58	0.72
NN to EPI	Day 1	EPI	0.50	0.39	0.59
NN to EPI	Day 1	change	-0.15	-0.28	-0.04
NN to EPI	Day 2	NN	0.60	0.51	0.67
NN to EPI	Day 2	EPI	0.51	0.40	0.62
NN to EPI	Day 2	change	-0.08	-0.22	0.06
NN to EPI	Day 3	NN	0.52	0.43	0.62
NN to EPI	Day 3	EPI	0.52	0.39	0.65
NN to EPI	Day 3	change	0.00	-0.17	0.15
EPI to NN	Overall	NN	0.60	0.52	0.68
EPI to NN	Overall	EPI	0.51	0.47	0.54
EPI to NN	Overall	change	0.09	0.01	0.17
EPI to NN	Day 0	NN	0.58	0.49	0.68
EPI to NN	Day 0	EPI	0.41	0.33	0.48

EPI to NN	Day 0	change	0.17	0.06	0.29
EPI to NN	Day 1	NN	0.61	0.52	0.69
EPI to NN	Day 1	EPI	0.48	0.41	0.54
EPI to NN	Day 1	change	0.13	0.03	0.25
EPI to NN	Day 2	NN	0.61	0.52	0.72
EPI to NN	Day 2	EPI	0.54	0.47	0.60
EPI to NN	Day 2	change	0.08	-0.04	0.19
EPI to NN	Day 3	NN	0.58	0.47	0.70
EPI to NN	Day 3	EPI	0.58	0.50	0.65
EPI to NN	Day 3	change	0.00	-0.13	0.14
SPI to EPI	Overall	SPI	0.61	0.54	0.67
SPI to EPI	Overall	EPI	0.51	0.41	0.60
SPI to EPI	Overall	change	-0.10	-0.22	0.00
SPI to EPI	Day 0	SPI	0.58	0.46	0.70
SPI to EPI	Day 0	EPI	0.44	0.30	0.58
SPI to EPI	Day 0	change	-0.12	-0.32	0.04
SPI to EPI	Day 1	SPI	0.59	0.51	0.71
SPI to EPI	Day 1	EPI	0.48	0.35	0.60
SPI to EPI	Day 1	change	-0.12	-0.27	0.04
SPI to EPI	Day 2	SPI	0.61	0.51	0.71
SPI to EPI	Day 2	EPI	0.51	0.38	0.64
SPI to EPI	Day 2	change	-0.10	-0.28	0.06
SPI to EPI	Day 3	SPI	0.67	0.54	0.77
SPI to EPI	Day 3	EPI	0.60	0.47	0.74
SPI to EPI	Day 3	change	-0.07	-0.25	0.10
EPI to SPI	Overall	SPI	0.60	0.51	0.67
EPI to SPI	Overall	EPI	0.51	0.47	0.55
EPI to SPI	Overall	change	0.09	0.01	0.19
EPI to SPI	Day 0	SPI	0.52	0.40	0.61
EPI to SPI	Day 0	EPI	0.41	0.34	0.48
EPI to SPI	Day 0	change	0.10	-0.02	0.22
EPI to SPI	Day 1	SPI	0.58	0.48	0.67
EPI to SPI	Day 1	EPI	0.48	0.41	0.54
EPI to SPI	Day 1	change	0.10	-0.01	0.21
EPI to SPI	Day 2	SPI	0.63	0.53	0.72
EPI to SPI	Day 2	EPI	0.54	0.47	0.61
EPI to SPI	Day 2	change	0.08	-0.03	0.21
EPI to SPI	Day 3	SPI	0.65	0.54	0.76

EPI to SPI	Day 3	EPI	0.58	0.50	0.64
EPI to SPI	Day 3	change	0.07	-0.08	0.19

4.2.2 by Surgical Approach

Figure 9

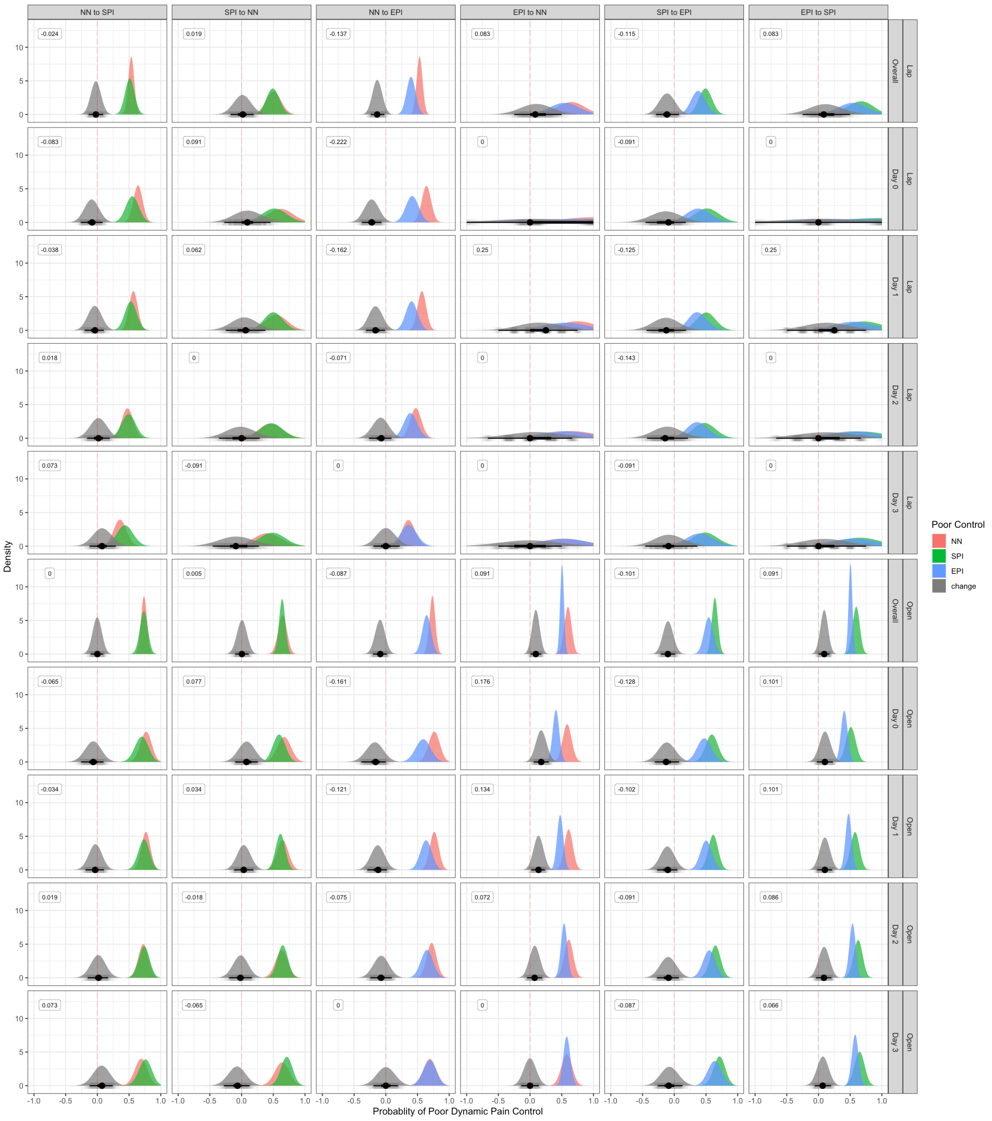

Table 5

SurgAppr	AnalgesicTechniques	Day	Analg	ProbPoorControl.median	CI95low	CI95
Lap	NN to SPI	Overall	NN	0.53	0.47	
Lap	NN to SPI	Overall	SPI	0.51	0.41	
Lap	NN to SPI	Overall	change	-0.02	-0.14	
Lap	NN to SPI	Day 0	NN	0.64	0.54	

Lap	NN to SPI	Day 0	SPI	0.56	0.42
Lap	NN to SPI	Day 0	change	-0.08	-0.26
Lap	NN to SPI	Day 1	NN	0.56	0.46
Lap	NN to SPI	Day 1	SPI	0.52	0.40
Lap	NN to SPI	Day 1	change	-0.04	-0.20
Lap	NN to SPI	Day 2	NN	0.48	0.38
Lap	NN to SPI	Day 2	SPI	0.50	0.32
Lap	NN to SPI	Day 2	change	0.02	-0.16
Lap	NN to SPI	Day 3	NN	0.37	0.22
Lap	NN to SPI	Day 3	SPI	0.44	0.27
Lap	NN to SPI	Day 3	change	0.07	-0.12
Lap	SPI to NN	Overall	NN	0.50	0.35
Lap	SPI to NN	Overall	SPI	0.50	0.36
Lap	SPI to NN	Overall	change	0.02	-0.19
Lap	SPI to NN	Day 0	NN	0.64	0.36
Lap	SPI to NN	Day 0	SPI	0.54	0.27
Lap	SPI to NN	Day 0	change	0.09	-0.27
Lap	SPI to NN	Day 1	NN	0.56	0.31
Lap	SPI to NN	Day 1	SPI	0.50	0.25
Lap	SPI to NN	Day 1	change	0.06	-0.25
Lap	SPI to NN	Day 2	NN	0.43	0.14
Lap	SPI to NN	Day 2	SPI	0.50	0.21
Lap	SPI to NN	Day 2	change	0.00	-0.36
Lap	SPI to NN	Day 3	NN	0.36	0.09
Lap	SPI to NN	Day 3	SPI	0.46	0.18
Lap	SPI to NN	Day 3	change	-0.09	-0.46
Lap	NN to EPI	Overall	NN	0.53	0.47
Lap	NN to EPI	Overall	EPI	0.40	0.30
Lap	NN to EPI	Overall	change	-0.14	-0.24
Lap	NN to EPI	Day 0	NN	0.64	0.53
Lap	NN to EPI	Day 0	EPI	0.42	0.28
Lap	NN to EPI	Day 0	change	-0.22	-0.38
Lap	NN to EPI	Day 1	NN	0.58	0.46
Lap	NN to EPI	Day 1	EPI	0.41	0.28
Lap	NN to EPI	Day 1	change	-0.16	-0.31
Lap	NN to EPI	Day 2	NN	0.48	0.36
Lap	NN to EPI	Day 2	EPI	0.39	0.25
Lap	NN to EPI	Day 2	change	-0.07	-0.27

Lap	NN to EPI	Day 3	NN	0.37	0.22
Lap	NN to EPI	Day 3	EPI	0.37	0.17
Lap	NN to EPI	Day 3	change	0.00	-0.20
Lap	EPI to NN	Overall	NN	0.67	0.33
Lap	EPI to NN	Overall	EPI	0.50	0.25
Lap	EPI to NN	Overall	change	0.08	-0.25
Lap	EPI to NN	Day 0	NN	1.00	0.00
Lap	EPI to NN	Day 0	EPI	1.00	0.00
Lap	EPI to NN	Day 0	change	0.00	-1.00
Lap	EPI to NN	Day 1	NN	0.75	0.25
Lap	EPI to NN	Day 1	EPI	0.50	0.25
Lap	EPI to NN	Day 1	change	0.25	-0.25
Lap	EPI to NN	Day 2	NN	0.67	0.00
Lap	EPI to NN	Day 2	EPI	0.67	0.00
Lap	EPI to NN	Day 2	change	0.00	-0.67
Lap	EPI to NN	Day 3	NN	0.50	0.00
Lap	EPI to NN	Day 3	EPI	0.50	0.00
Lap	EPI to NN	Day 3	change	0.00	-0.50
Lap	SPI to EPI	Overall	SPI	0.50	0.36
Lap	SPI to EPI	Overall	EPI	0.38	0.23
Lap	SPI to EPI	Overall	change	-0.12	-0.29
Lap	SPI to EPI	Day 0	SPI	0.54	0.27
Lap	SPI to EPI	Day 0	EPI	0.36	0.18
Lap	SPI to EPI	Day 0	change	-0.09	-0.46
Lap	SPI to EPI	Day 1	SPI	0.50	0.25
Lap	SPI to EPI	Day 1	EPI	0.38	0.12
Lap	SPI to EPI	Day 1	change	-0.12	-0.44
Lap	SPI to EPI	Day 2	SPI	0.50	0.29
Lap	SPI to EPI	Day 2	EPI	0.36	0.14
Lap	SPI to EPI	Day 2	change	-0.14	-0.43
Lap	SPI to EPI	Day 3	SPI	0.46	0.18
Lap	SPI to EPI	Day 3	EPI	0.36	0.09
Lap	SPI to EPI	Day 3	change	-0.09	-0.46
Lap	EPI to SPI	Overall	SPI	0.67	0.33
Lap	EPI to SPI	Overall	EPI	0.58	0.25
Lap	EPI to SPI	Overall	change	0.08	-0.25
Lap	EPI to SPI	Day 0	SPI	1.00	0.00
Lap	EPI to SPI	Day 0	EPI	1.00	0.00

Lap	EPI to SPI	Day 0	change	0.00	-1.00
Lap	EPI to SPI	Day 1	SPI	0.75	0.25
Lap	EPI to SPI	Day 1	EPI	0.50	0.25
Lap	EPI to SPI	Day 1	change	0.25	-0.50
Lap	EPI to SPI	Day 2	SPI	0.67	0.33
Lap	EPI to SPI	Day 2	EPI	0.67	0.00
Lap	EPI to SPI	Day 2	change	0.00	-0.67
Lap	EPI to SPI	Day 3	SPI	0.75	0.25
Lap	EPI to SPI	Day 3	EPI	0.50	0.25
Lap	EPI to SPI	Day 3	change	0.00	-0.50
Open	NN to SPI	Overall	NN	0.74	0.67
Open	NN to SPI	Overall	SPI	0.73	0.64
Open	NN to SPI	Overall	change	0.00	-0.10
Open	NN to SPI	Day 0	NN	0.77	0.64
Open	NN to SPI	Day 0	SPI	0.71	0.52
Open	NN to SPI	Day 0	change	-0.06	-0.26
Open	NN to SPI	Day 1	NN	0.76	0.67
Open	NN to SPI	Day 1	SPI	0.74	0.62
Open	NN to SPI	Day 1	change	-0.03	-0.17
Open	NN to SPI	Day 2	NN	0.72	0.58
Open	NN to SPI	Day 2	SPI	0.74	0.58
Open	NN to SPI	Day 2	change	0.02	-0.17
Open	NN to SPI	Day 3	NN	0.68	0.56
Open	NN to SPI	Day 3	SPI	0.76	0.61
Open	NN to SPI	Day 3	change	0.07	-0.12
Open	SPI to NN	Overall	NN	0.65	0.55
Open	SPI to NN	Overall	SPI	0.64	0.57
Open	SPI to NN	Overall	change	0.00	-0.10
Open	SPI to NN	Day 0	NN	0.67	0.54
Open	SPI to NN	Day 0	SPI	0.59	0.46
Open	SPI to NN	Day 0	change	0.08	-0.10
Open	SPI to NN	Day 1	NN	0.64	0.52
Open	SPI to NN	Day 1	SPI	0.61	0.52
Open	SPI to NN	Day 1	change	0.03	-0.14
Open	SPI to NN	Day 2	NN	0.64	0.47
Open	SPI to NN	Day 2	SPI	0.66	0.53
Open	SPI to NN	Day 2	change	-0.02	-0.20
Open	SPI to NN	Day 3	NN	0.63	0.50

Open	SPI to NN	Day 3	SPI	0.72	0.59
Open	SPI to NN	Day 3	change	-0.06	-0.28
Open	NN to EPI	Overall	NN	0.74	0.67
Open	NN to EPI	Overall	EPI	0.64	0.55
Open	NN to EPI	Overall	change	-0.09	-0.20
Open	NN to EPI	Day 0	NN	0.77	0.64
Open	NN to EPI	Day 0	EPI	0.58	0.42
Open	NN to EPI	Day 0	change	-0.16	-0.39
Open	NN to EPI	Day 1	NN	0.76	0.67
Open	NN to EPI	Day 1	EPI	0.64	0.52
Open	NN to EPI	Day 1	change	-0.12	-0.29
Open	NN to EPI	Day 2	NN	0.72	0.60
Open	NN to EPI	Day 2	EPI	0.64	0.51
Open	NN to EPI	Day 2	change	-0.07	-0.26
Open	NN to EPI	Day 3	NN	0.68	0.56
Open	NN to EPI	Day 3	EPI	0.71	0.54
Open	NN to EPI	Day 3	change	0.00	-0.20
Open	EPI to NN	Overall	NN	0.60	0.51
Open	EPI to NN	Overall	EPI	0.50	0.47
Open	EPI to NN	Overall	change	0.09	0.01
Open	EPI to NN	Day 0	NN	0.59	0.50
Open	EPI to NN	Day 0	EPI	0.41	0.34
Open	EPI to NN	Day 0	change	0.18	0.06
Open	EPI to NN	Day 1	NN	0.61	0.51
Open	EPI to NN	Day 1	EPI	0.48	0.41
Open	EPI to NN	Day 1	change	0.13	0.03
Open	EPI to NN	Day 2	NN	0.61	0.51
Open	EPI to NN	Day 2	EPI	0.54	0.47
Open	EPI to NN	Day 2	change	0.07	-0.04
Open	EPI to NN	Day 3	NN	0.58	0.46
Open	EPI to NN	Day 3	EPI	0.58	0.50
Open	EPI to NN	Day 3	change	0.00	-0.14
Open	SPI to EPI	Overall	SPI	0.64	0.57
Open	SPI to EPI	Overall	EPI	0.54	0.44
Open	SPI to EPI	Overall	change	-0.10	-0.22
Open	SPI to EPI	Day 0	SPI	0.59	0.46
Open	SPI to EPI	Day 0	EPI	0.46	0.31

Open	SPI to EPI	Day 0	change	-0.13	-0.31
Open	SPI to EPI	Day 1	SPI	0.61	0.51
Open	SPI to EPI	Day 1	EPI	0.51	0.39
Open	SPI to EPI	Day 1	change	-0.10	-0.27
Open	SPI to EPI	Day 2	SPI	0.66	0.53
Open	SPI to EPI	Day 2	EPI	0.54	0.40
Open	SPI to EPI	Day 2	change	-0.09	-0.29
Open	SPI to EPI	Day 3	SPI	0.72	0.59
Open	SPI to EPI	Day 3	EPI	0.63	0.50
Open	SPI to EPI	Day 3	change	-0.09	-0.28
Open	EPI to SPI	Overall	SPI	0.60	0.51
Open	EPI to SPI	Overall	EPI	0.50	0.46
Open	EPI to SPI	Overall	change	0.09	0.00
Open	EPI to SPI	Day 0	SPI	0.51	0.42
Open	EPI to SPI	Day 0	EPI	0.40	0.33
Open	EPI to SPI	Day 0	change	0.10	-0.02
Open	EPI to SPI	Day 1	SPI	0.58	0.48
Open	EPI to SPI	Day 1	EPI	0.48	0.41
Open	EPI to SPI	Day 1	change	0.10	-0.03
Open	EPI to SPI	Day 2	SPI	0.62	0.52
Open	EPI to SPI	Day 2	EPI	0.54	0.47
Open	EPI to SPI	Day 2	change	0.09	-0.04
Open	EPI to SPI	Day 3	SPI	0.65	0.52
Open	EPI to SPI	Day 3	EPI	0.58	0.50
Open	EPI to SPI	Day 3	change	0.07	-0.07

4.2.3 by Surgical Type

Figure 10

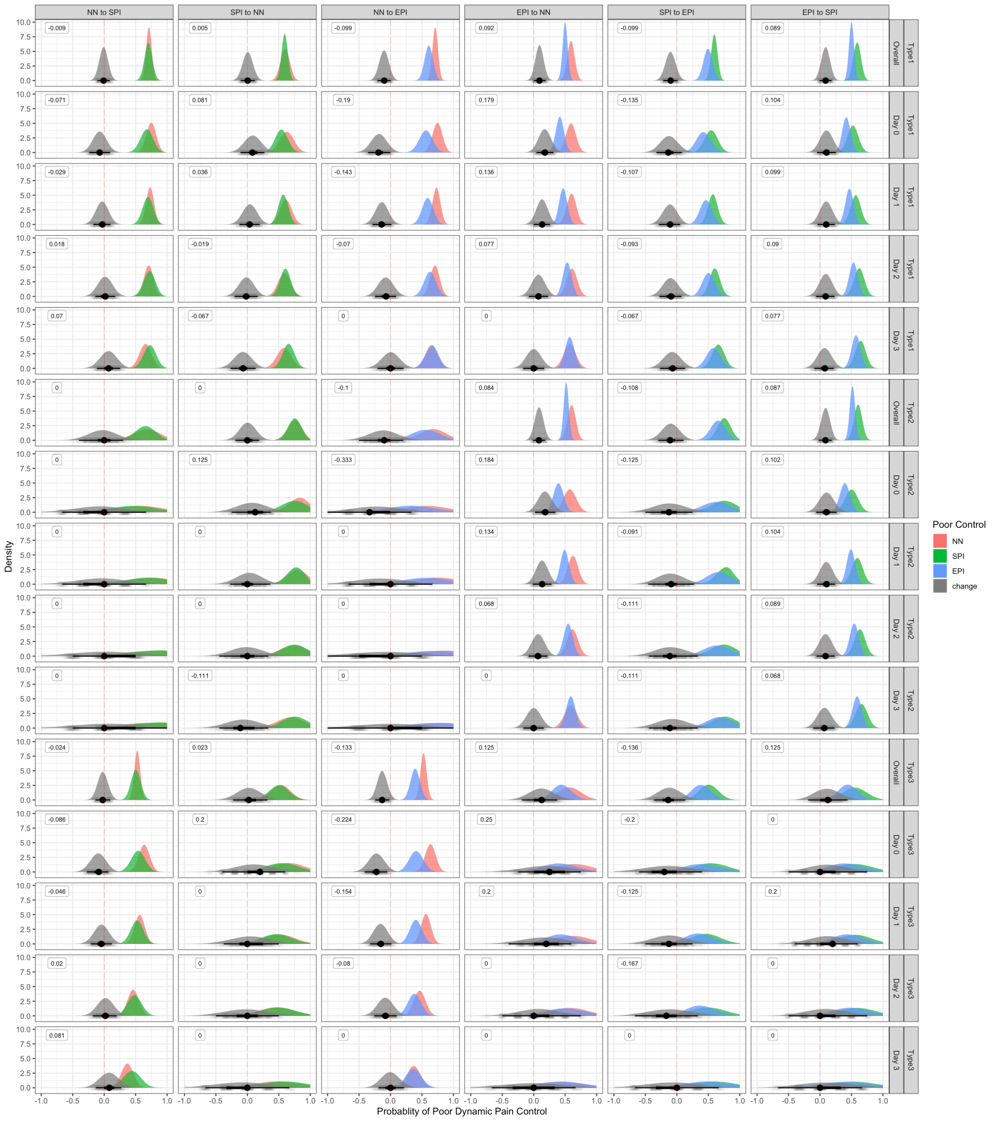

Table 6

SurgType	AnalgesicTechniques	Day	Analg	ProbPoorControl.median	CI95low	CI95
Type1	NN to SPI	Overall	NN	0.71	0.65	
Type1	NN to SPI	Overall	SPI	0.70	0.62	
Type1	NN to SPI	Overall	change	-0.01	-0.11	
Type1	NN to SPI	Day 0	NN	0.74	0.64	
Type1	NN to SPI	Day 0	SPI	0.69	0.52	
Type1	NN to SPI	Day 0	change	-0.07	-0.24	
Type1	NN to SPI	Day 1	NN	0.73	0.64	
Type1	NN to SPI	Day 1	SPI	0.70	0.59	
Type1	NN to SPI	Day 1	change	-0.03	-0.16	
Type1	NN to SPI	Day 2	NN	0.70	0.58	
Type1	NN to SPI	Day 2	SPI	0.72	0.60	
Type1	NN to SPI	Day 2	change	0.02	-0.14	

Type1	NN to SPI	Day 3	NN	0.65	0.51
Type1	NN to SPI	Day 3	SPI	0.72	0.58
Type1	NN to SPI	Day 3	change	0.07	-0.12
Type1	SPI to NN	Overall	NN	0.60	0.50
Type1	SPI to NN	Overall	SPI	0.59	0.53
Type1	SPI to NN	Overall	change	0.00	-0.10
Type1	SPI to NN	Day 0	NN	0.62	0.49
Type1	SPI to NN	Day 0	SPI	0.54	0.41
Type1	SPI to NN	Day 0	change	0.08	-0.11
Type1	SPI to NN	Day 1	NN	0.61	0.48
Type1	SPI to NN	Day 1	SPI	0.57	0.45
Type1	SPI to NN	Day 1	change	0.04	-0.12
Type1	SPI to NN	Day 2	NN	0.59	0.46
Type1	SPI to NN	Day 2	SPI	0.61	0.50
Type1	SPI to NN	Day 2	change	-0.02	-0.20
Type1	SPI to NN	Day 3	NN	0.58	0.42
Type1	SPI to NN	Day 3	SPI	0.64	0.53
Type1	SPI to NN	Day 3	change	-0.07	-0.27
Type1	NN to EPI	Overall	NN	0.71	0.65
Type1	NN to EPI	Overall	EPI	0.61	0.51
Type1	NN to EPI	Overall	change	-0.10	-0.20
Type1	NN to EPI	Day 0	NN	0.74	0.64
Type1	NN to EPI	Day 0	EPI	0.57	0.41
Type1	NN to EPI	Day 0	change	-0.19	-0.33
Type1	NN to EPI	Day 1	NN	0.73	0.63
Type1	NN to EPI	Day 1	EPI	0.59	0.47
Type1	NN to EPI	Day 1	change	-0.14	-0.29
Type1	NN to EPI	Day 2	NN	0.70	0.58
Type1	NN to EPI	Day 2	EPI	0.63	0.51
Type1	NN to EPI	Day 2	change	-0.07	-0.26
Type1	NN to EPI	Day 3	NN	0.65	0.51
Type1	NN to EPI	Day 3	EPI	0.65	0.49
Type1	NN to EPI	Day 3	change	0.00	-0.21
Type1	EPI to NN	Overall	NN	0.59	0.51
Type1	EPI to NN	Overall	EPI	0.50	0.44
Type1	EPI to NN	Overall	change	0.09	0.00
Type1	EPI to NN	Day 0	NN	0.60	0.49
Type1	EPI to NN	Day 0	EPI	0.42	0.33

Type1	EPI to NN	Day 0	change	0.18	0.03
Type1	EPI to NN	Day 1	NN	0.60	0.51
Type1	EPI to NN	Day 1	EPI	0.47	0.37
Type1	EPI to NN	Day 1	change	0.14	0.00
Type1	EPI to NN	Day 2	NN	0.60	0.50
Type1	EPI to NN	Day 2	EPI	0.54	0.45
Type1	EPI to NN	Day 2	change	0.08	-0.08
Type1	EPI to NN	Day 3	NN	0.58	0.42
Type1	EPI to NN	Day 3	EPI	0.58	0.46
Type1	EPI to NN	Day 3	change	0.00	-0.15
Type1	SPI to EPI	Overall	SPI	0.59	0.53
Type1	SPI to EPI	Overall	EPI	0.50	0.38
Type1	SPI to EPI	Overall	change	-0.10	-0.21
Type1	SPI to EPI	Day 0	SPI	0.54	0.41
Type1	SPI to EPI	Day 0	EPI	0.41	0.27
Type1	SPI to EPI	Day 0	change	-0.14	-0.35
Type1	SPI to EPI	Day 1	SPI	0.57	0.46
Type1	SPI to EPI	Day 1	EPI	0.46	0.34
Type1	SPI to EPI	Day 1	change	-0.11	-0.29
Type1	SPI to EPI	Day 2	SPI	0.61	0.50
Type1	SPI to EPI	Day 2	EPI	0.50	0.39
Type1	SPI to EPI	Day 2	change	-0.09	-0.28
Type1	SPI to EPI	Day 3	SPI	0.67	0.53
Type1	SPI to EPI	Day 3	EPI	0.58	0.42
Type1	SPI to EPI	Day 3	change	-0.07	-0.27
Type1	EPI to SPI	Overall	SPI	0.59	0.51
Type1	EPI to SPI	Overall	EPI	0.50	0.44
Type1	EPI to SPI	Overall	change	0.09	-0.01
Type1	EPI to SPI	Day 0	SPI	0.52	0.40
Type1	EPI to SPI	Day 0	EPI	0.42	0.33
Type1	EPI to SPI	Day 0	change	0.10	-0.04
Type1	EPI to SPI	Day 1	SPI	0.57	0.44
Type1	EPI to SPI	Day 1	EPI	0.47	0.37
Type1	EPI to SPI	Day 1	change	0.10	-0.03
Type1	EPI to SPI	Day 2	SPI	0.63	0.50
Type1	EPI to SPI	Day 2	EPI	0.53	0.44
Type1	EPI to SPI	Day 2	change	0.09	-0.06
Type1	EPI to SPI	Day 3	SPI	0.64	0.51

Type1	EPI to SPI	Day 3	EPI	0.58	0.46
Type1	EPI to SPI	Day 3	change	0.08	-0.09
Type2	NN to SPI	Overall	NN	0.70	0.40
Type2	NN to SPI	Overall	SPI	0.70	0.40
Type2	NN to SPI	Overall	change	0.00	-0.30
Type2	NN to SPI	Day 0	NN	0.67	0.00
Type2	NN to SPI	Day 0	SPI	0.33	0.00
Type2	NN to SPI	Day 0	change	0.00	-0.67
Type2	NN to SPI	Day 1	NN	0.67	0.33
Type2	NN to SPI	Day 1	SPI	0.67	0.33
Type2	NN to SPI	Day 1	change	0.00	-0.67
Type2	NN to SPI	Day 2	NN	1.00	0.50
Type2	NN to SPI	Day 2	SPI	1.00	0.50
Type2	NN to SPI	Day 2	change	0.00	-0.50
Type2	NN to SPI	Day 3	NN	0.50	0.00
Type2	NN to SPI	Day 3	SPI	1.00	0.50
Type2	NN to SPI	Day 3	change	0.00	-0.50
Type2	SPI to NN	Overall	NN	0.76	0.60
Type2	SPI to NN	Overall	SPI	0.76	0.62
Type2	SPI to NN	Overall	change	0.00	-0.19
Type2	SPI to NN	Day 0	NN	0.88	0.62
Type2	SPI to NN	Day 0	SPI	0.75	0.50
Type2	SPI to NN	Day 0	change	0.12	-0.25
Type2	SPI to NN	Day 1	NN	0.82	0.64
Type2	SPI to NN	Day 1	SPI	0.73	0.46
Type2	SPI to NN	Day 1	change	0.00	-0.27
Type2	SPI to NN	Day 2	NN	0.78	0.44
Type2	SPI to NN	Day 2	SPI	0.78	0.44
Type2	SPI to NN	Day 2	change	0.00	-0.44
Type2	SPI to NN	Day 3	NN	0.67	0.33
Type2	SPI to NN	Day 3	SPI	0.78	0.56
Type2	SPI to NN	Day 3	change	-0.11	-0.44
Type2	NN to EPI	Overall	NN	0.70	0.40
Type2	NN to EPI	Overall	EPI	0.60	0.30
Type2	NN to EPI	Overall	change	-0.10	-0.50
Type2	NN to EPI	Day 0	NN	0.67	0.00
Type2	NN to EPI	Day 0	EPI	0.33	0.00
Type2	NN to EPI	Day 0	change	-0.33	-0.67

Type2	NN to EPI	Day 1	NN	0.67	0.33
Type2	NN to EPI	Day 1	EPI	0.67	0.33
Type2	NN to EPI	Day 1	change	0.00	-0.67
Type2	NN to EPI	Day 2	NN	1.00	0.50
Type2	NN to EPI	Day 2	EPI	0.50	0.00
Type2	NN to EPI	Day 2	change	0.00	-1.00
Type2	NN to EPI	Day 3	NN	0.50	0.00
Type2	NN to EPI	Day 3	EPI	0.50	0.00
Type2	NN to EPI	Day 3	change	0.00	-1.00
Type2	EPI to NN	Overall	NN	0.60	0.52
Type2	EPI to NN	Overall	EPI	0.52	0.46
Type2	EPI to NN	Overall	change	0.08	-0.01
Type2	EPI to NN	Day 0	NN	0.57	0.43
Type2	EPI to NN	Day 0	EPI	0.39	0.29
Type2	EPI to NN	Day 0	change	0.18	0.02
Type2	EPI to NN	Day 1	NN	0.63	0.51
Type2	EPI to NN	Day 1	EPI	0.49	0.40
Type2	EPI to NN	Day 1	change	0.13	-0.03
Type2	EPI to NN	Day 2	NN	0.62	0.49
Type2	EPI to NN	Day 2	EPI	0.55	0.44
Type2	EPI to NN	Day 2	change	0.07	-0.08
Type2	EPI to NN	Day 3	NN	0.60	0.45
Type2	EPI to NN	Day 3	EPI	0.60	0.49
Type2	EPI to NN	Day 3	change	0.00	-0.18
Type2	SPI to EPI	Overall	SPI	0.76	0.62
Type2	SPI to EPI	Overall	EPI	0.65	0.49
Type2	SPI to EPI	Overall	change	-0.11	-0.27
Type2	SPI to EPI	Day 0	SPI	0.75	0.50
Type2	SPI to EPI	Day 0	EPI	0.62	0.38
Type2	SPI to EPI	Day 0	change	-0.12	-0.50
Type2	SPI to EPI	Day 1	SPI	0.82	0.46
Type2	SPI to EPI	Day 1	EPI	0.64	0.46
Type2	SPI to EPI	Day 1	change	-0.09	-0.46
Type2	SPI to EPI	Day 2	SPI	0.78	0.44
Type2	SPI to EPI	Day 2	EPI	0.67	0.33
Type2	SPI to EPI	Day 2	change	-0.11	-0.44
Type2	SPI to EPI	Day 3	SPI	0.78	0.56
Type2	SPI to EPI	Day 3	EPI	0.67	0.33

Type2	SPI to EPI	Day 3	change	-0.11	-0.44
Type2	EPI to SPI	Overall	SPI	0.60	0.51
Type2	EPI to SPI	Overall	EPI	0.52	0.46
Type2	EPI to SPI	Overall	change	0.09	-0.02
Type2	EPI to SPI	Day 0	SPI	0.51	0.37
Type2	EPI to SPI	Day 0	EPI	0.39	0.29
Type2	EPI to SPI	Day 0	change	0.10	-0.06
Type2	EPI to SPI	Day 1	SPI	0.60	0.48
Type2	EPI to SPI	Day 1	EPI	0.49	0.40
Type2	EPI to SPI	Day 1	change	0.10	-0.04
Type2	EPI to SPI	Day 2	SPI	0.63	0.49
Type2	EPI to SPI	Day 2	EPI	0.55	0.44
Type2	EPI to SPI	Day 2	change	0.09	-0.07
Type2	EPI to SPI	Day 3	SPI	0.66	0.53
Type2	EPI to SPI	Day 3	EPI	0.60	0.47
Type2	EPI to SPI	Day 3	change	0.07	-0.11
Type3	NN to SPI	Overall	NN	0.52	0.46
Type3	NN to SPI	Overall	SPI	0.50	0.39
Type3	NN to SPI	Overall	change	-0.02	-0.14
Type3	NN to SPI	Day 0	NN	0.64	0.52
Type3	NN to SPI	Day 0	SPI	0.53	0.40
Type3	NN to SPI	Day 0	change	-0.09	-0.29
Type3	NN to SPI	Day 1	NN	0.57	0.46
Type3	NN to SPI	Day 1	SPI	0.52	0.38
Type3	NN to SPI	Day 1	change	-0.05	-0.22
Type3	NN to SPI	Day 2	NN	0.46	0.32
Type3	NN to SPI	Day 2	SPI	0.48	0.32
Type3	NN to SPI	Day 2	change	0.02	-0.20
Type3	NN to SPI	Day 3	NN	0.38	0.22
Type3	NN to SPI	Day 3	SPI	0.43	0.27
Type3	NN to SPI	Day 3	change	0.08	-0.14
Type3	SPI to NN	Overall	NN	0.54	0.32
Type3	SPI to NN	Overall	SPI	0.50	0.27
Type3	SPI to NN	Overall	change	0.02	-0.27
Type3	SPI to NN	Day 0	NN	0.60	0.40
Type3	SPI to NN	Day 0	SPI	0.60	0.00
Type3	SPI to NN	Day 0	change	0.20	-0.40

Type3	SPI to NN	Day 1	NN	0.50	0.12
Type3	SPI to NN	Day 1	SPI	0.50	0.12
Type3	SPI to NN	Day 1	change	0.00	-0.38
Type3	SPI to NN	Day 2	NN	0.50	0.17
Type3	SPI to NN	Day 2	SPI	0.50	0.17
Type3	SPI to NN	Day 2	change	0.00	-0.67
Type3	SPI to NN	Day 3	NN	0.33	0.00
Type3	SPI to NN	Day 3	SPI	0.67	0.33
Type3	SPI to NN	Day 3	change	0.00	-1.00
Type3	NN to EPI	Overall	NN	0.52	0.46
Type3	NN to EPI	Overall	EPI	0.40	0.29
Type3	NN to EPI	Overall	change	-0.13	-0.24
Type3	NN to EPI	Day 0	NN	0.64	0.52
Type3	NN to EPI	Day 0	EPI	0.41	0.26
Type3	NN to EPI	Day 0	change	-0.22	-0.41
Type3	NN to EPI	Day 1	NN	0.57	0.45
Type3	NN to EPI	Day 1	EPI	0.40	0.28
Type3	NN to EPI	Day 1	change	-0.15	-0.32
Type3	NN to EPI	Day 2	NN	0.46	0.34
Type3	NN to EPI	Day 2	EPI	0.38	0.22
Type3	NN to EPI	Day 2	change	-0.08	-0.28
Type3	NN to EPI	Day 3	NN	0.38	0.24
Type3	NN to EPI	Day 3	EPI	0.38	0.22
Type3	NN to EPI	Day 3	change	0.00	-0.19
Type3	EPI to NN	Overall	NN	0.56	0.31
Type3	EPI to NN	Overall	EPI	0.44	0.25
Type3	EPI to NN	Overall	change	0.12	-0.19
Type3	EPI to NN	Day 0	NN	0.75	0.25
Type3	EPI to NN	Day 0	EPI	0.50	0.25
Type3	EPI to NN	Day 0	change	0.25	-0.25
Type3	EPI to NN	Day 1	NN	0.60	0.20
Type3	EPI to NN	Day 1	EPI	0.40	0.20
Type3	EPI to NN	Day 1	change	0.20	-0.20
Type3	EPI to NN	Day 2	NN	0.50	0.25
Type3	EPI to NN	Day 2	EPI	0.50	0.00
Type3	EPI to NN	Day 2	change	0.00	-0.50
Type3	EPI to NN	Day 3	NN	0.33	0.00
Type3	EPI to NN	Day 3	EPI	0.33	0.00

Type3	EPI to NN	Day 3	change	0.00	-0.67
Type3	SPI to EPI	Overall	SPI	0.50	0.27
Type3	SPI to EPI	Overall	EPI	0.36	0.18
Type3	SPI to EPI	Overall	change	-0.14	-0.36
Type3	SPI to EPI	Day 0	SPI	0.60	0.00
Type3	SPI to EPI	Day 0	EPI	0.40	0.00
Type3	SPI to EPI	Day 0	change	-0.20	-0.80
Type3	SPI to EPI	Day 1	SPI	0.50	0.12
Type3	SPI to EPI	Day 1	EPI	0.38	0.12
Type3	SPI to EPI	Day 1	change	-0.12	-0.50
Type3	SPI to EPI	Day 2	SPI	0.50	0.17
Type3	SPI to EPI	Day 2	EPI	0.33	0.00
Type3	SPI to EPI	Day 2	change	-0.17	-0.50
Type3	SPI to EPI	Day 3	SPI	0.67	0.00
Type3	SPI to EPI	Day 3	EPI	0.33	0.00
Type3	SPI to EPI	Day 3	change	0.00	-1.00
Type3	EPI to SPI	Overall	SPI	0.56	0.25
Type3	EPI to SPI	Overall	EPI	0.44	0.19
Type3	EPI to SPI	Overall	change	0.12	-0.19
Type3	EPI to SPI	Day 0	SPI	0.50	0.25
Type3	EPI to SPI	Day 0	EPI	0.50	0.25
Type3	EPI to SPI	Day 0	change	0.00	-0.50
Type3	EPI to SPI	Day 1	SPI	0.60	0.20
Type3	EPI to SPI	Day 1	EPI	0.40	0.20
Type3	EPI to SPI	Day 1	change	0.20	-0.40
Type3	EPI to SPI	Day 2	SPI	0.50	0.25
Type3	EPI to SPI	Day 2	EPI	0.50	0.00
Type3	EPI to SPI	Day 2	change	0.00	-0.50
Type3	EPI to SPI	Day 3	SPI	0.67	0.00
Type3	EPI to SPI	Day 3	EPI	0.33	0.00
Type3	EPI to SPI	Day 3	change	0.00	-0.67

5 Software and packages used

The model was implemented with the brms package in R which provides an interface to fit Bayesian generalized multivariate multilevel models using the probabilistic programming language Stan. cmdstanr package was used to interface with Stan from R. Packages used and version details are provided below.

```
## [1] "Fri Jul 22 15:07:48 2022"
```

```
## R version 4.2.1 (2022-06-23)
## Platform: x86_64-apple-darwin17.0 (64-bit)
## Running under: macOS Big Sur ... 10.16
##
## Matrix products: default
## BLAS:   /Library/Frameworks/R.framework/Versions/4.2/Resources/lib
/libRblas.0.dylib
## LAPACK: /Library/Frameworks/R.framework/Versions/4.2/Resources/lib
/libRlapack.dylib
##
## locale:
## [1] en_AU.UTF-8/en_AU.UTF-8/en_AU.UTF-8/C/en_AU.UTF-8/en_AU.UTF-8
##
## attached base packages:
## [1] stats     graphics  grDevices utils     datasets  methods   ba
se
##
## other attached packages:
##  [1] magrittr_2.0.3     lubridate_1.8.0    readxl_1.4.0       skim
r_2.1.4
##  [5] tictoc_1.0.1       bayesplot_1.9.0    bayestestR_0.12.1  quan
treg_5.93
##  [9] SparseM_1.81       brms_2.17.0        Rcpp_1.0.9         DT_0
.23
## [13] summarytools_1.0.1 plotrix_3.8-2      ggthemes_4.2.4     forc
ats_0.5.1
## [17] stringr_1.4.0      dplyr_1.0.9        purrr_0.3.4        read
r_2.1.2
## [21] tidyr_1.2.0        tibble_3.1.7       ggplot2_3.3.6      tidy
verse_1.3.2
##
## loaded via a namespace (and not attached):
##   [1] backports_1.4.1      plyr_1.8.7           igraph_1.3.3
##   [4] repr_1.1.4           splines_4.2.1        crosstalk_1.2.0
##   [7] rstantools_2.2.0     inline_0.3.19        pryr_0.1.5
##  [10] digest_0.6.29        htmltools_0.5.3      magick_2.7.3
##  [13] fansi_1.0.3          checkmate_2.1.0      googlesheets4_1.0.
0
##  [16] tzdb_0.3.0           modelr_0.1.8         RcppParallel_5.1.5
##  [19] matrixStats_0.62.0   xts_0.12.1           prettyunits_1.1.1
##  [22] colorspace_2.0-3     rvest_1.0.2          ggdist_3.1.1
##  [25] haven_2.5.0          xfun_0.31            tcltk_4.2.1
##  [28] callr_3.7.1          crayon_1.5.1         jsonlite_1.8.0
##  [31] survival_3.3-1       zoo_1.8-10           glue_1.6.2
##  [34] gtable_0.3.0         gargle_1.2.0         MatrixModels_0.5-0
##  [37] distributional_0.3.0 car_3.1-0            pkgbuild_1.3.1
##  [40] rstan_2.21.5         abind_1.4-5          rapportools_1.1
##  [43] scales_1.2.0         mvtnorm_1.1-3        DBI_1.1.3
##  [46] ggeffects_1.1.2      rstatix_0.7.0        miniUI_0.1.1.1
##  [49] xtable_1.8-4         HDInterval_0.2.2     stats4_4.2.1
##  [52] StanHeaders_2.21.0-7 datawizard_0.4.1     htmlwidgets_1.5.4
##  [55] httr_1.4.3           threejs_0.3.3        posterior_1.2.2
##  [58] ellipsis_0.3.2       pkgconfig_2.0.3      loo_2.5.1
##  [61] farver_2.1.1         sass_0.4.2           dbplyr_2.2.1
##  [64] utf8_1.2.2           labeling_0.4.2       tidyselect_1.1.2
##  [67] rlang_1.0.4          reshape2_1.4.4       later_1.3.0
##  [70] munsell_0.5.0        cellranger_1.1.0     tools_4.2.1
##  [73] cachem_1.0.6         cli_3.3.0            generics_0.1.3
##  [76] broom_1.0.0          ggridges_0.5.3       evaluate_0.15
##  [79] fastmap_1.1.0        yaml_2.3.5           processx_3.7.0
##  [82] knitr_1.39           fs_1.5.2             pander_0.6.5
##  [85] nlme_3.1-158         mime_0.12            xml2_1.3.3
```

```
##  [88] compiler_4.2.1       shinythemes_1.2.0    rstudioapi_0.13
##  [91] ggsignif_0.6.3       reprex_2.0.1         bslib_0.4.0
##  [94] stringi_1.7.8        highr_0.9            ps_1.7.1
##  [97] Brobdingnag_1.2-7    lattice_0.20-45      Matrix_1.4-1
## [100] markdown_1.1         shinyjs_2.1.0        tensorA_0.36.2
## [103] vctrs_0.4.1          pillar_1.8.0         lifecycle_1.0.1
## [106] jquerylib_0.1.4      bridgesampling_1.1-2 cowplot_1.1.1
## [109] data.table_1.14.2    insight_0.18.0       httpuv_1.6.5
## [112] R6_2.5.1             promises_1.2.0.1     gridExtra_2.3
## [115] codetools_0.2-18     colourpicker_1.1.1   MASS_7.3-58
## [118] gtools_3.9.3         assertthat_0.2.1     withr_2.5.0
## [121] shinystan_2.6.0      parallel_4.2.1       hms_1.1.1
## [124] grid_4.2.1           coda_0.19-4          cmdstanr_0.5.2
## [127] rmarkdown_2.14       carData_3.0-5        googledrive_2.0.0
## [130] ggpubr_0.4.0         shiny_1.7.2          base64enc_0.1-3
## [133] dygraphs_1.1.1.6
```

```
## 1559.029 sec elapsed
```
